# Diastereo- and enantioselective additions of α-nitro esters to imines for *anti*-α,β-diamino acid synthesis with α-alkyl-substitution†
†Electronic supplementary information (ESI) available. See DOI: 10.1039/c7sc05176j


**DOI:** 10.1039/c7sc05176j

**Published:** 2018-01-31

**Authors:** Daniel J. Sprague, Anand Singh, Jeffrey N. Johnston

**Affiliations:** a Department of Chemistry , Vanderbilt Institute of Chemical Biology , Vanderbilt University , Nashville , Tennessee 37235 , USA . Email: jeffrey.n.johnston@vanderbilt.edu

## Abstract

The discovery that a *C*_2_-symmetric bis(AMidine) [BAM] catalyst promotes an *anti*-selective addition of α-substituted α-nitro esters to imines is described, providing α-substituted α,β-diamino ester products with high diastereo- and enantioselectivity. When compared to the function of a BAM catalyst reported previously, the pair offer a rare example of diastereodivergence using a bifunctional Brønsted acid–base organocatalyst.

## Abstract 2

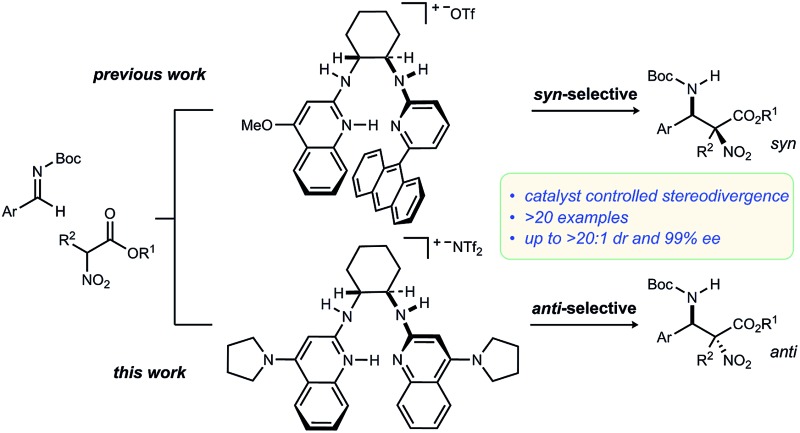
The discovery that a *C*_2_-symmetric bis(AMidine) [BAM] catalyst promotes an *anti*-selective addition of α-substituted α-nitro esters to imines is described, providing α-substituted α,β-diamino ester products with high diastereo- and enantioselectivity.

## 

Methods to prepare enantioenriched α-amino acids are in demand, and there are few direct solutions to those bearing α-alkyl substituents.^1^ Fewer still are methods that deliver α,β-diamino acids bearing α-alkyl substituents.^2^ These unnatural amino acids are desirable precursors to peptide sequences due to their effect on the conformation and activity of the peptide sequences into which they are incorporated.^3^–^5^ The enantioselective *aza*-Henry (nitro-Mannich) reaction^6^ serves as a convergent approach to α,β-diamino acid derivatives, but its adaptation to α-alkyl-α-nitroester substrates (Scheme 1) is more rare, owing to the congestion provided by the additional substituent. Within this realm are the highly diastereoselective examples of the *anti*-selective reaction by Jorgensen,^7^ Shibasaki,^8^ Wu,^9^ and Huang-Dong.^10^ In contrast, *syn*-selective reactions are the exception,^11^ with reports by us^12^ and Ooi.^13^ Diastereodivergence in enantioselective catalysis is a characteristic driving modern catalyst development,^14^–^16^ and it motivated us to develop an *anti*-selective variant using the same bifunctional Brønsted acid/base catalyst design (Scheme 1).^12^ We report the finding that a *C*_2_-symmetric ligand design, in combination with sterically hindered esters of α-nitro acids, can lead to highly *anti*-diastereoselective and enantioselective additions to *N*-Boc imine electrophiles. This creates a rare example in which a pair of organocatalyzed reactions with generally conserved design features, exhibit diastereodivergence and high selectivity.^14^,^17^,^18^

**Scheme 1:**
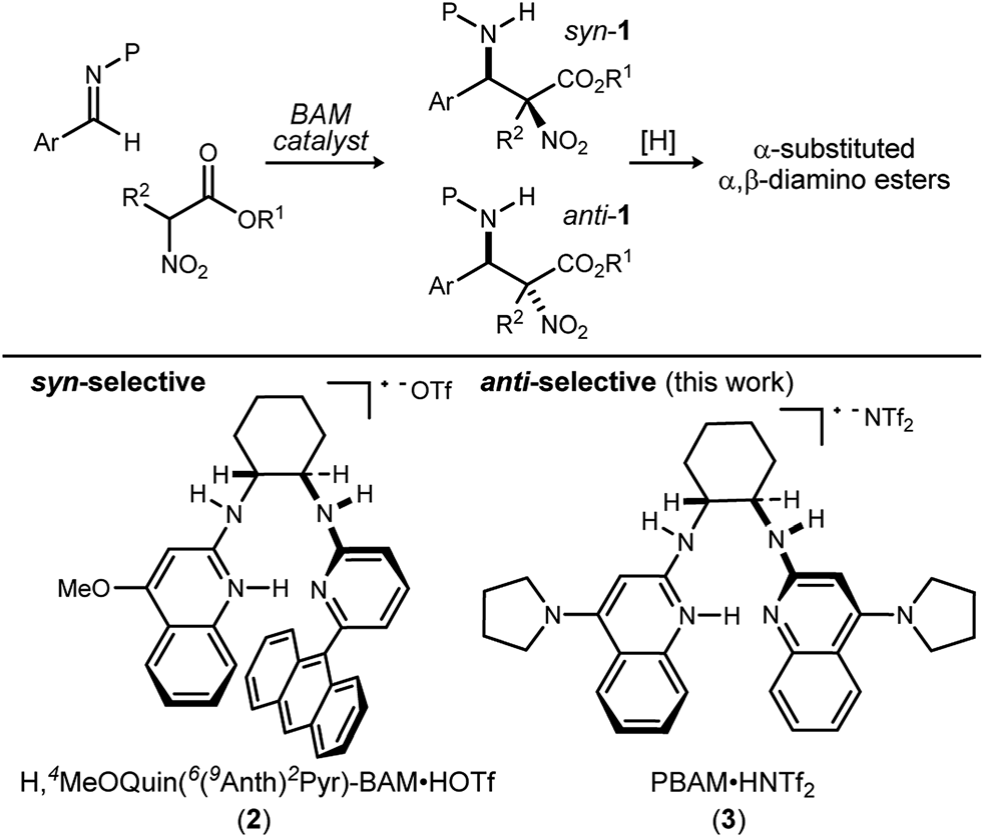
Development of a diastereodivergent *aza*-Henry reaction of α-alkyl α,β-nitroesters: *syn*-selective (prior work) and *anti*-selective (this work) catalysts using a common bifunctional design.

We previously reported the organocatalytic synthesis of α-substituted *syn*-α,β-diamino acid derivatives *syn*-**1**.^12^ Key to that success was the finding that unsymmetrical quinoline catalyst **2**^19^ was necessary to achieve adequate reactivity, wherein the methoxy substituent imparted a more Brønsted basic 2-aminoquinoline for efficient activation of the sterically demanding nitro ester pronucleophile.^20^ Additionally, hindered aryl esters found synergism with the crowded pocket of **2** to provide high *syn*-selectivity, good yield, and high enantioselection.^21^–^23^

A return to symmetrical catalyst **3**^24^ (Scheme 1) was made in order to examine the impact of a less congested binding pocket to selectivity. In doing so, retention of catalyst activation using a pyrrolidine at the quinoline 4-position was anticipated. In the event, the level of diastereoselection with a small alkyl ester was low, but again increased with ester size (as in Scheme 2) and with the distinction that the *anti*-diastereomer was favored. As before, the ester size works synergistically with the catalyst to achieve increasing levels of selectivity, particularly diastereoselectivity (**10d** → **11d**, 10 : 1 dr, 94% ee). Finally, changing the solvent to toluene and the counteranion to triflimide afforded a combination producing optimal stereoselectivity (Table 1, entry 1) overall.^25^

**Scheme 2:**
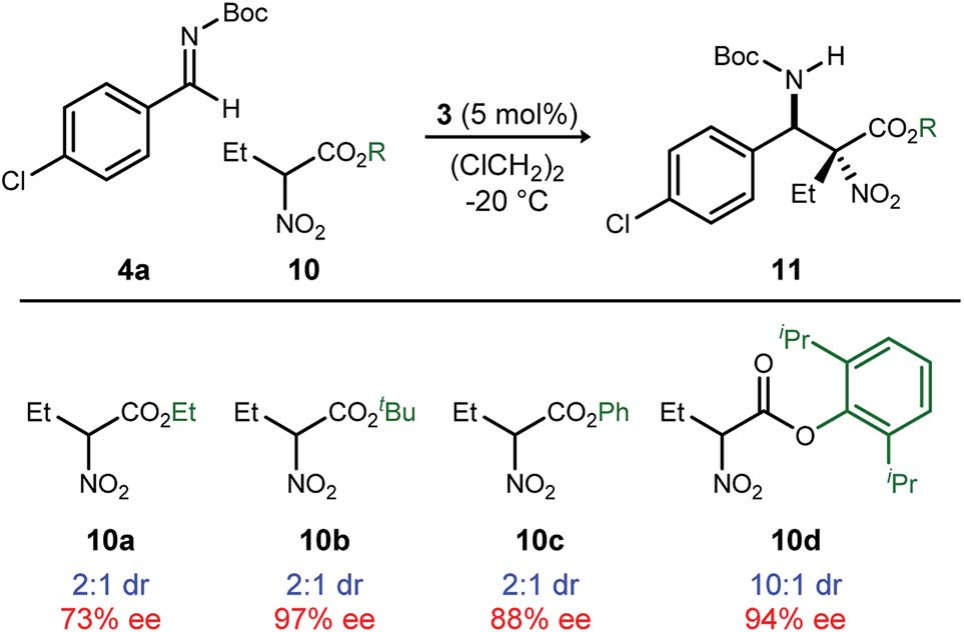
Determinants of diastereoselection: synergism between catalyst **3** and ester size.

**Table 1:** *anti*-Selective chiral proton-catalyzed additions of α-alkyl α-nitro esters to azomethines: nucleophile scope^*a*^

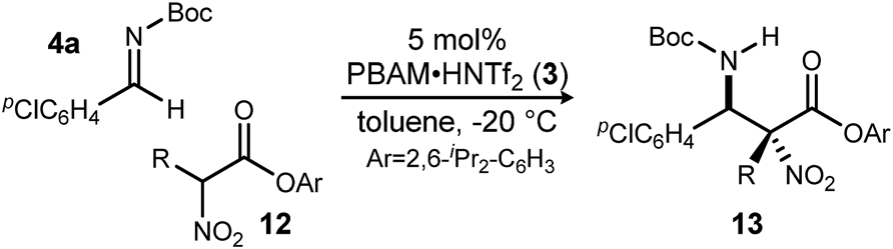					
Entry^*a*^	R	**13**	dr^*b*^	ee^*c*^	Yield^*d*^
1	Me	**a**	>20 : 1	99	70
2^*e*^	Et	**b**	>20 : 1	99	66
3	^*n*^Pr	**c**	>20 : 1	96	72
4	^*n*^Bu	**d**	11 : 1	97	64
5	Allyl	**e**	9 : 1	97	71
6	Bn	**f**	4 : 1	83	65
7	^*c*^Pr	**g**	15 : 1	98	68
8	^*i*^Pr	**h**	>20 : 1	93	66
9	^*c*^Hex	**i**	>20 : 1	87	46

^*a*^All reactions were 0.7 M in imine, used 1.1 equiv. of the α-nitro ester, and had a standard 48 h reaction time.

^*b*^Diastereomer ratios measured using ^1^H NMR.

^*c*^Enantiomeric ratios measured using HPLC and a chiral stationary phase.

^*d*^Yields are for isolated, analytically pure adduct.

^*e*^For comparison, use of the triflic acid salt of the catalyst provides this *anti*-product in 17 : 1 dr and 97% ee. *rac*-PBAM (free base) affords the adduct in 2 : 1 dr.

Having maximized the favored ester/catalyst combination to effect high *anti*-selectivity while maintaining high enantioselection, we turned to an evaluation of substrate scope. The effect of the size of the alkyl substituent presented by the hindered nitro ester was probed first by increasing chain length (Table 1, entries 1–4) using ^*p*^Cl-phenyl aldimine **4a** as a standard electrophile. α-Nitro propionoate (**12a**), butanoate (**10d**/**12b**), pentanoate (**12c**), and hexanoate (**12d**) each afforded product in good yield with excellent diastereoselection (11 : 1 → 20 : 1 dr) and uniformly high enantioselection (96–99% ee). As this substituent is changed further, only those with sp^2^-hybridization resulted in lower diastereoselection (down to 4 : 1) (Table 1, entries 5–6).‡
‡These diastereomers are separable using silica gel chromatography. Branching alkyl substituents, however, returned selectivity to >15 : 1 dr (Table 1, entries 7–9). α-Cyclopropyl nitroacetate **12g** afforded product in 68% isolated yield with 15 : 1 dr and 98% ee (Table 1, entry 7), and α-isopropyl nitroacetate **12h** afforded product in 66% isolated yield with >20 : 1 dr and 93% ee (Table 1, entry 8). α-Cyclohexyl nitroacetate **12i** gave the desired diamine derivative in >20 : 1 dr, and 87% ee, albeit in a lower isolated yield (46%, Table 1, entry 9). The lower conversion, and consequently lower isolated yield, reflect the steric bulk surrounding the nucleophilic carbon. Nevertheless, synthetically useful amounts of stereoenriched product **13i** can be obtained under the reaction conditions. An allyl group was incorporated at the α-position in good isolated yield, dr, and high ee (Table 1, entry 5). This installs a handle for further synthetic manipulations.

With these results in hand, α-nitro butanoate **10d**/**12b** was employed as a standard pronucleophile to evaluate an electronically and sterically diverse group of aldimines in the reaction (Table 2). Electronically neutral aldimines (Table 2, entries 1, 4, 9, 10, and 13) resulted in good isolated yield (54–76%), high diastereoselection (12 : 1 → >20 : 1 dr) and high enantioselection (96–99% ee). Notably, sterically demanding 1-naphthyl (Table 2, entry 9) and *para*-phenyl benzaldimine (Table 2, entry 13) were tolerated well, with high stereoselection. Electron deficient aldimines were also competent electrophiles. Trifluoromethylphenyl-substituted imine **4t** (Table 2, entry 12) afforded adduct **13t** in 74% isolated yield with 15 : 1 dr and 97% ee. Both chloro- and bromo-substituted imines (Table 2, entries 2 and 3) afforded the corresponding adducts in good yield with excellent diastereoselection (>20 : 1 dr) and enantioselection (99% ee). Thiophenyl and pyridyl aldimines were equally amenable to addition (Table 2, entries 8 and 11). Electron-rich rings (Table 2, entries 5–7) afforded the *aza*-Henry adducts in good yields with notably lower diastereoselectivity, though enantioselectivity was generally maintained. The erosion of diastereoselection may be attributed to a less electrophilic azomethine, leading to a longer electrophile-nucleophile distance in the bond-forming step, or a diminished secondary interaction between the nitro and azomethine. Unfortunately, *N*-Boc ketimines exhibited their typical unreactive nature in this system, likely due to the severe steric congestion in the adducts, despite stirring at room temperature for 7 days. And while some product could be obtained using aliphatic *N*-Boc aldimines in exploratory experiments, selectivities were low.

**Table 2:** *anti*-Selective chiral proton-catalyzed additions of α-alkyl α-nitro esters to azomethines: electrophile scope^*a*^

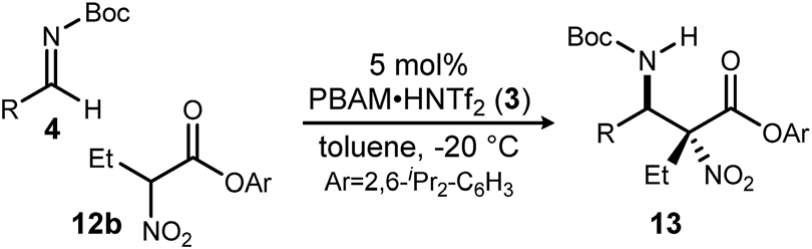					
Entry^*a*^	R	**13**	dr^*b*^	ee^*c*^	Yield^*d*^
1	C_6_H_5_	**j**	>20 : 1	96	76
2	^4^Cl–C_6_H_4_	**b**	>20 : 1	99	66
3	^4^Br–C_6_H_4_	**k**	>20 : 1	99	71
4	^3^Me–C_6_H_4_	**l**	12 : 1	97	71
5	^3^MeO–C_6_H_4_	**m**	5 : 1	96	71
6	^4^MeO–C_6_H_4_	**n**	5 : 1	78	68
7	^2^Furyl	**o**	4 : 1	91	63
8	^2^Thiophene	**p**	>20 : 1	97	63
9	^1^Naphthyl	**q**	15 : 1	99	54
10	^2^Naphthyl	**r**	>20 : 1	96	70
11	^3^Pyridyl	**s**	9 : 1	96	48
12	^4^CF_3_–C_6_H_4_	**t**	15 : 1	97	74
13	^4^Ph–C_6_H_4_	**u**	>20 : 1	99	73

^*a*^All reactions were 0.7 M in imine, used 1.1 equiv. of the α-nitro ester, and had a standard 48 h reaction time.

^*b*^Diastereomer ratios measured using ^1^H NMR.

^*c*^Enantiomeric ratios measured using HPLC and a chiral stationary phase.

^*d*^Yields are those of isolated, analytically pure adduct.

In addition to absolute and relative stereochemical assignment by X-ray for **11d**,^12^ the absolute stereochemistry of adduct **14**^26^ was assigned *via* chemical correlation to known compound **15**. (*S*,*S*)-**15** was reported to have a rotation of +44. Synthetic **15** using catalyst **3** exhibited a measured rotation of –39. Therefore, the adducts produced by (*R*,*R*)-PBAM·HNTf_2_ have the configuration of (*R*,*R*) as depicted in Scheme 3.^10^

**Scheme 3:**
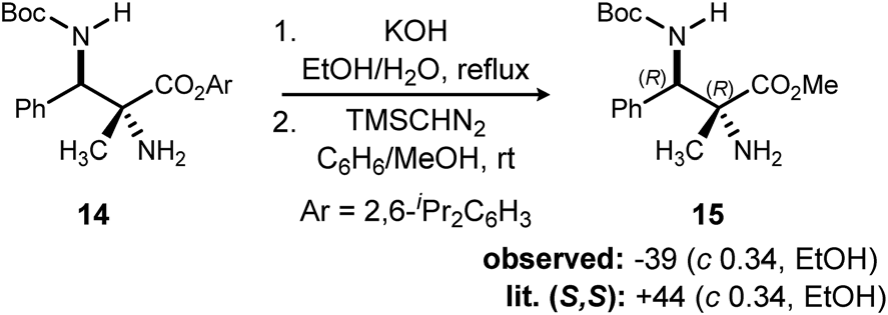
Determination of absolute and relative configuration by chemical correlation.

## Conclusions

In conclusion, we have developed the second enantioselective addition of α-alkyl α-nitro esters to imines using chiral proton catalysis, but with *anti*-diastereoselection. Taken together, these reactions are among the first highly selective hydrogen bond-catalyzed reactions exhibiting diastereodivergence. More remarkable is the use of a common catalyst design to reverse diastereoselection without compromise to enantioselection. We hypothesize that the key difference between **2** and **3** is the level of steric congestion in the binding pocket of the catalyst. Future studies will interrogate this hypothesis.

## Conflicts of interest

There are no conflicts to declare.

## Supplementary Material

Supplementary information

Supplementary information
