# Supplementary material for: Diastereo- and enantioselective additions of α-nitro esters to imines for anti-α,β-diamino acid synthesis with α-alkyl-substitution
Source: Chem Sci. 2018 Jan 31;9(8):2336–9. doi: 10.1039/c7sc05176j (PMC5903423; doi:10.1039/c7sc05176j)
Supplement: Supplementary file 1 [file SC-009-C7SC05176J-s001.pdf]

Electronic Supplementary Material (ESI) for Chemical Science.
This journal is © The Royal Society of Chemistry 2018

Diastereo- and Enantioselective Additions of α-Nitro Esters to Imines for *anti*-α,β-Diamino Acid Synthesis with α-Alkyl-Substitution

Daniel J. Sprague, Anand Singh, and Jeffrey N. Johnston*

Department of Chemistry & Vanderbilt Institute of Chemical Biology

Vanderbilt University

2301 Vanderbilt Place, Nashville, TN 37235-1822

S-II-X

Figure 1. ^{1}H NMR (400 MHz, $CDCl_3$) of 12e 4
Figure 2. ^{13}C NMR (100 MHz, $CDCl_3$) of 12e 5
Figure 3. ^{1}H NMR (500 MHz, $CDCl_3$) of 12f 6
Figure 4. ^{13}C NMR (125 MHz, $CDCl_3$) of 12f 7
Figure 5. ^{1}H NMR (500 MHz, $CDCl_3$) of 12g 8
Figure 6. ^{13}C NMR (125 MHz, $CDCl_3$) of 12g 9
Figure 7. ^{1}H NMR (400 MHz, $CDCl_3$) of 12i 10
Figure 8. ^{13}C NMR (100 MHz, $CDCl_3$) of 12i 11
Figure 9. ^{1}H NMR (400 MHz, $CDCl_3$) of 13a 12
Figure 10. ^{13}C NMR (100 MHz, $CDCl_3$) of 13a 13
Figure 11. ^{1}H NMR (400 MHz, $CDCl_3$) of 13b 14
Figure 12. ^{13}C NMR (100 MHz, $CDCl_3$) of 13b 15
Figure 13. ^{1}H NMR (400 MHz, $CDCl_3$) of 13c 16
Figure 14. ^{13}C NMR (100 MHz, $CDCl_3$) of 13c 17
Figure 15. ^{1}H NMR (400 MHz, $CDCl_3$) of 13d 18
Figure 16. ^{13}C NMR (100 MHz, $CDCl_3$) of 13d 19
Figure 17. ^{1}H NMR (400 MHz, $CDCl_3$) of 13e 20
Figure 18. ^{13}C NMR (100 MHz, $CDCl_3$) of 13e 21
Figure 19. ^{1}H NMR (400 MHz, $CDCl_3$) of 13f 22
Figure 20. ^{13}C NMR (100 MHz, $CDCl_3$) of 13f 23
Figure 21. ^{1}H NMR (400 MHz, $CDCl_3$) of 13g 24
Figure 22. ^{13}C NMR (100 MHz, $CDCl_3$) of 13g 25
Figure 23. ^{1}H NMR (400 MHz, $CDCl_3$) of 13h 26
Figure 24. ^{13}C NMR (100 MHz, $CDCl_3$) of 13h 27
Figure 25. ^{1}H NMR (400 MHz, $CDCl_3$) of 13i 28
Figure 26. ^{13}C NMR (100 MHz, $CDCl_3$) of 13i 29
Figure 27. ^{1}H NMR (500 MHz, DMSO-d_6) of 13j 30

Figure 28. ^{13}C NMR (125 MHz, DMSO-d_6) of 13j 31
Figure 29. ^{1}H NMR (400 MHz, $CDCl_3$) of 13k 32
Figure 30. ^{13}C NMR (100 MHz, $CDCl_3$) of 13k 33
Figure 31. ^{1}H NMR (400 MHz, $CDCl_3$) of 13l 34
Figure 32. ^{13}C NMR (100 MHz, $CDCl_3$) of 13l 35
Figure 33. ^{1}H NMR (400 MHz, $CDCl_3$) of 13m 36
Figure 34. ^{13}C NMR (100 MHz, $CDCl_3$) of 13m 37
Figure 35. ^{1}H NMR (400 MHz, $CDCl_3$) of 13o 38
Figure 36. ^{13}C NMR (100 MHz, $CDCl_3$) of 13o 39
Figure 37. ^{1}H NMR (400 MHz, $CDCl_3$) of 13p 40
Figure 38. ^{13}C NMR (100 MHz, $CDCl_3$) of 13p 41
Figure 39. ^{1}H NMR (400 MHz, $CDCl_3$) of 13r 42
Figure 40. ^{13}C NMR (100 MHz, $CDCl_3$) of 13r 43
Figure 41. ^{1}H NMR (500 MHz, $CDCl_3$) of 13s 44
Figure 42. ^{13}C NMR (125 MHz, $CDCl_3$) of 13s 45
Figure 43. ^{1}H NMR (500 MHz, $CDCl_3$) of 13t 46
Figure 44. ^{13}C NMR (125 MHz, $CDCl_3$) of 13t 47
Figure 45. ^{1}H NMR (400 MHz, $CDCl_3$) of 13u 48
Figure 46. ^{13}C NMR (100 MHz, $CDCl_3$) of 13u 49
Figure 49. ^{1}H NMR (400 MHz, $CDCl_3$) of 14 50
Figure 50. ^{13}C NMR (100 MHz, $CDCl_3$) of 14 51
Figure 47. ^{1}H NMR (500 MHz, DMSO-d_6) of SI-1 52
Figure 48. ^{13}C NMR (125 MHz, DMSO-d_6) of SI-1 53
Figure 59. HPLC trace of 13a 54
Figure 60. HPLC trace of 13b 55
Figure 61. HPLC trace of 13c 56
Figure 62. HPLC trace of 13d 57
Figure 63. HPLC trace of 13e 58
Figure 64. HPLC trace of 13f 59
Figure 65. HPLC trace of 13g 60
Figure 66. HPLC trace of 13h 61
Figure 67. HPLC trace of 13i 62
Figure 68. HPLC trace of 13j 63
Figure 69. HPLC trace of 13k 64
Figure 70. HPLC trace of 13l 65

Figure 71. HPLC trace of 13m 66
Figure 72. HPLC trace of 13o 67
Figure 73. HPLC trace of 13p 68
Figure 74. HPLC trace of 13r 69
Figure 75. HPLC trace of 13s 70
Figure 76. HPLC trace of 13t 71
Figure 77. HPLC trace of 13u 72
Figure 78. HPLC trace of SI-1 73

Figure 1. ^{1}H NMR (400 MHz, $CDCl_3$) of **12e**

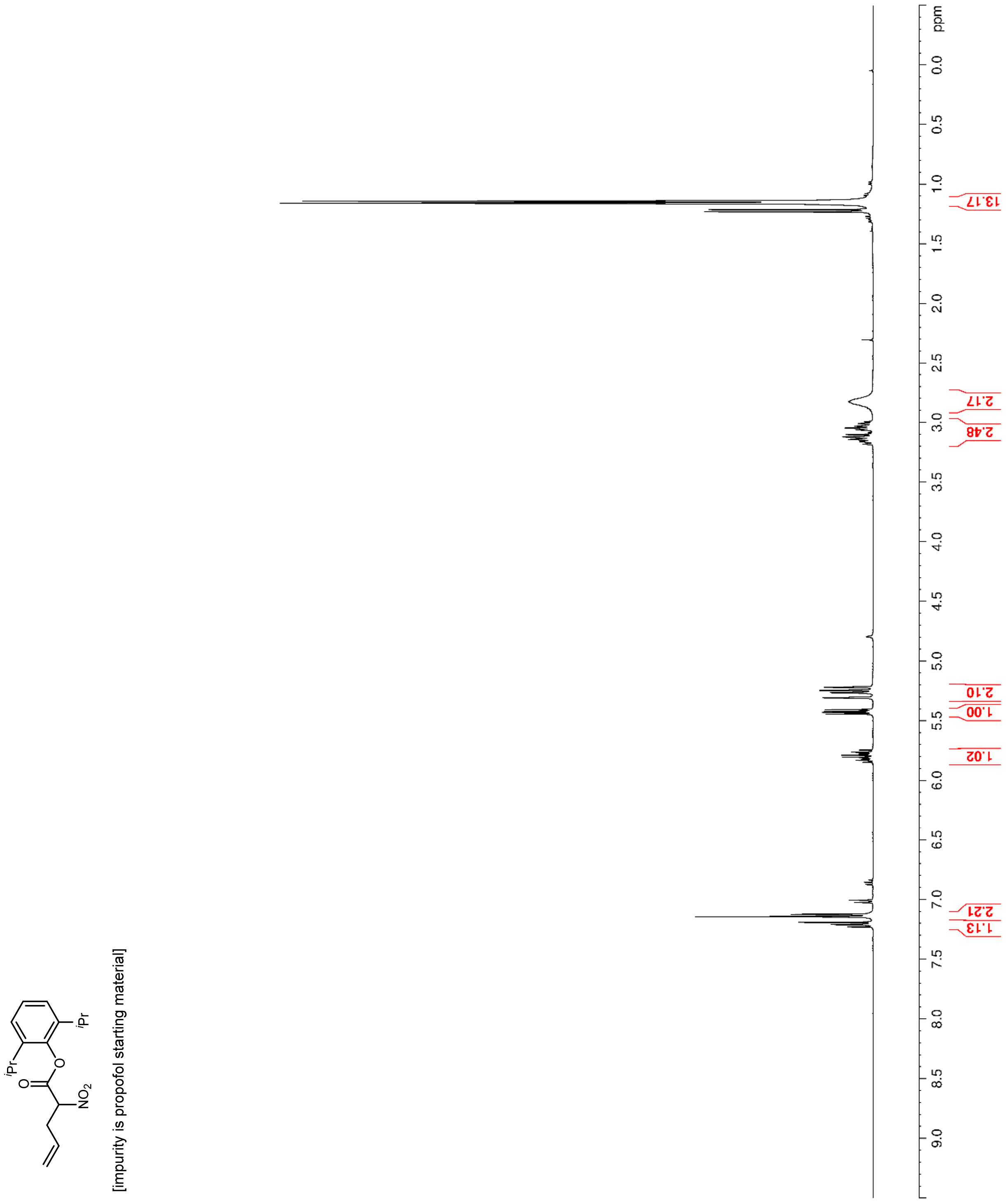

Figure 2. ^{13}C NMR (100 MHz, $CDCl_3$) of **12e**

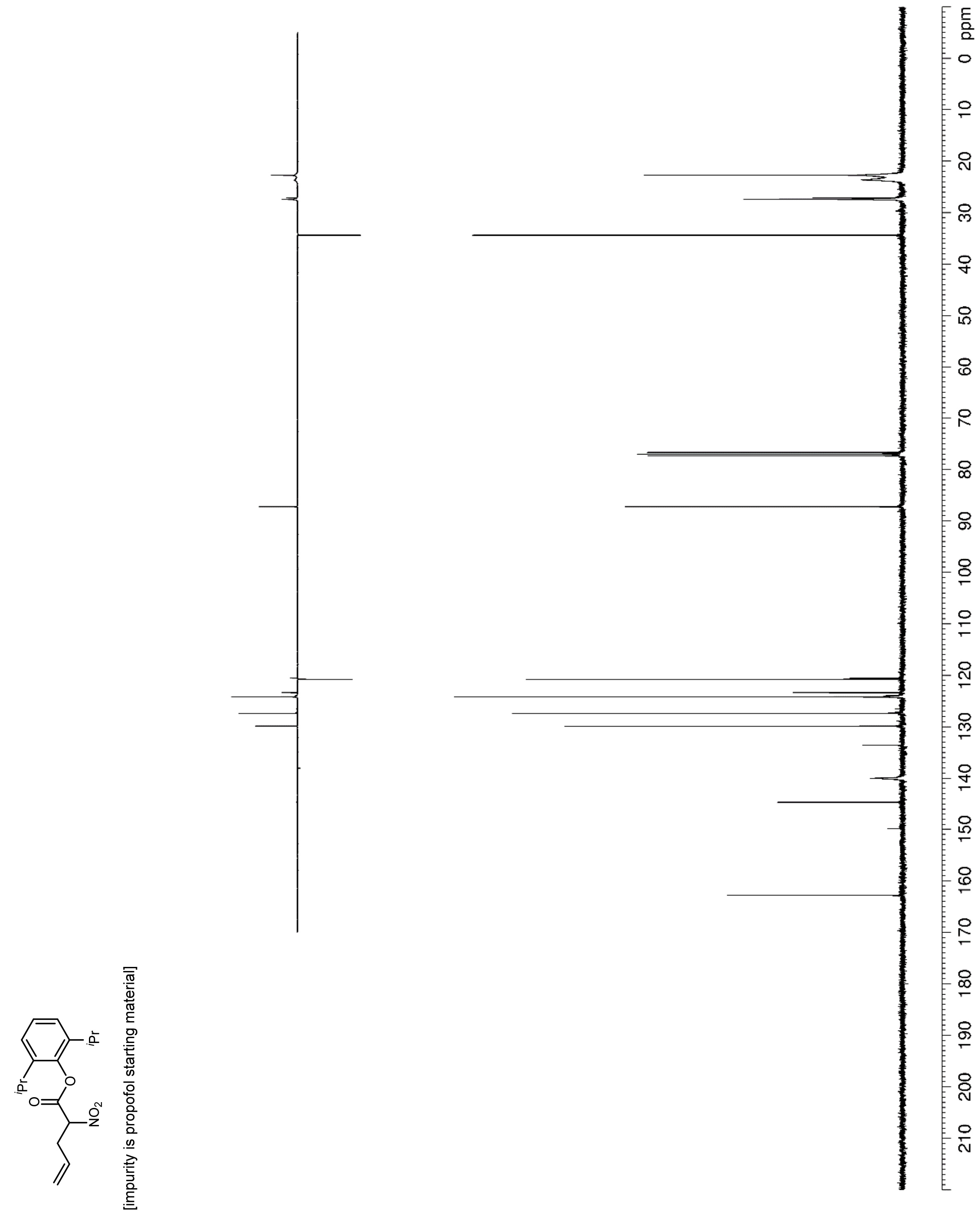

Figure 3. ^{1}H NMR (500 MHz, $CDCl_3$) of **12f**

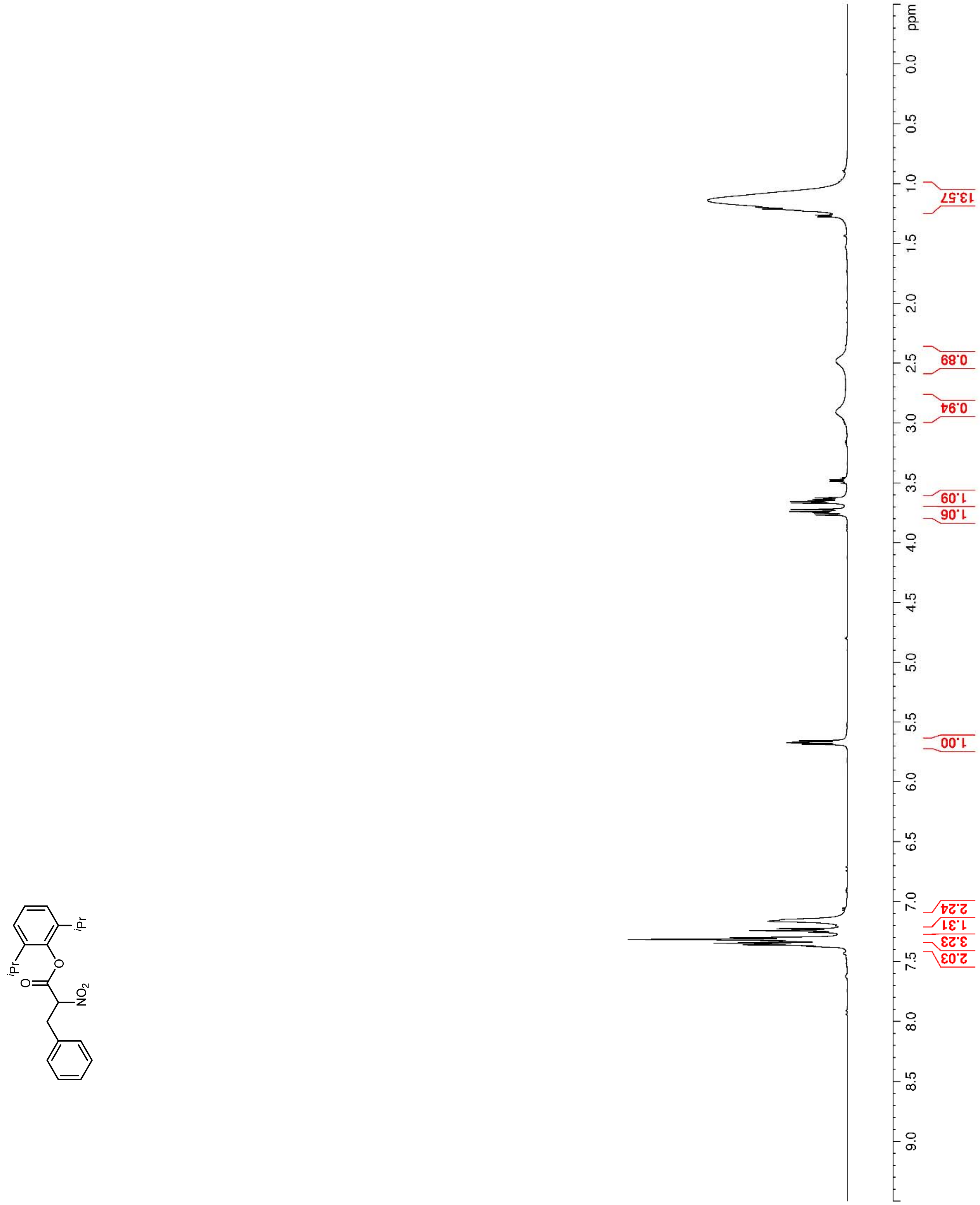

Figure 4. ^{13}C NMR (125 MHz, $CDCl_3$) of **12f**

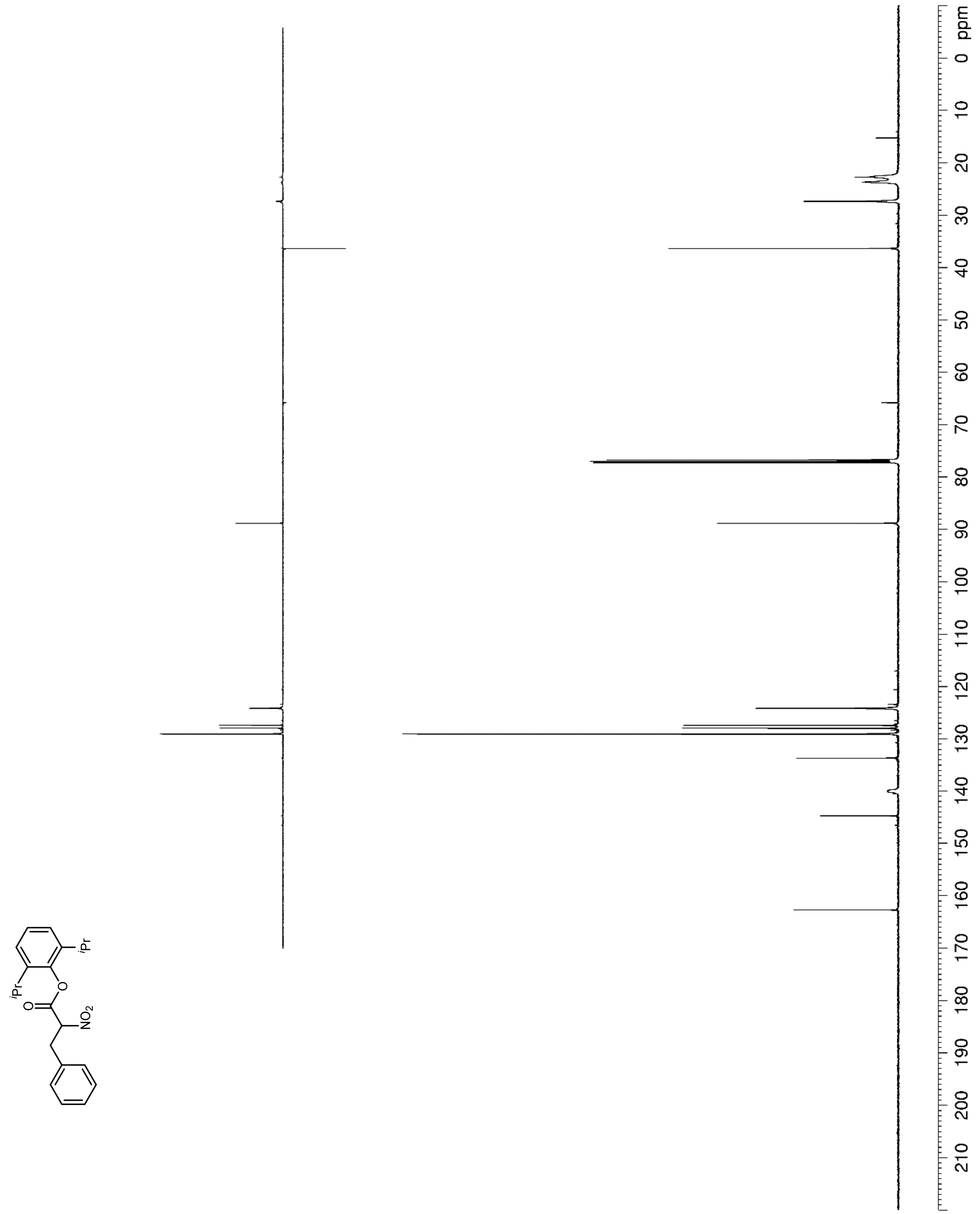

Figure 5. ^{1}H NMR (500 MHz, $CDCl_3$) of **12g**

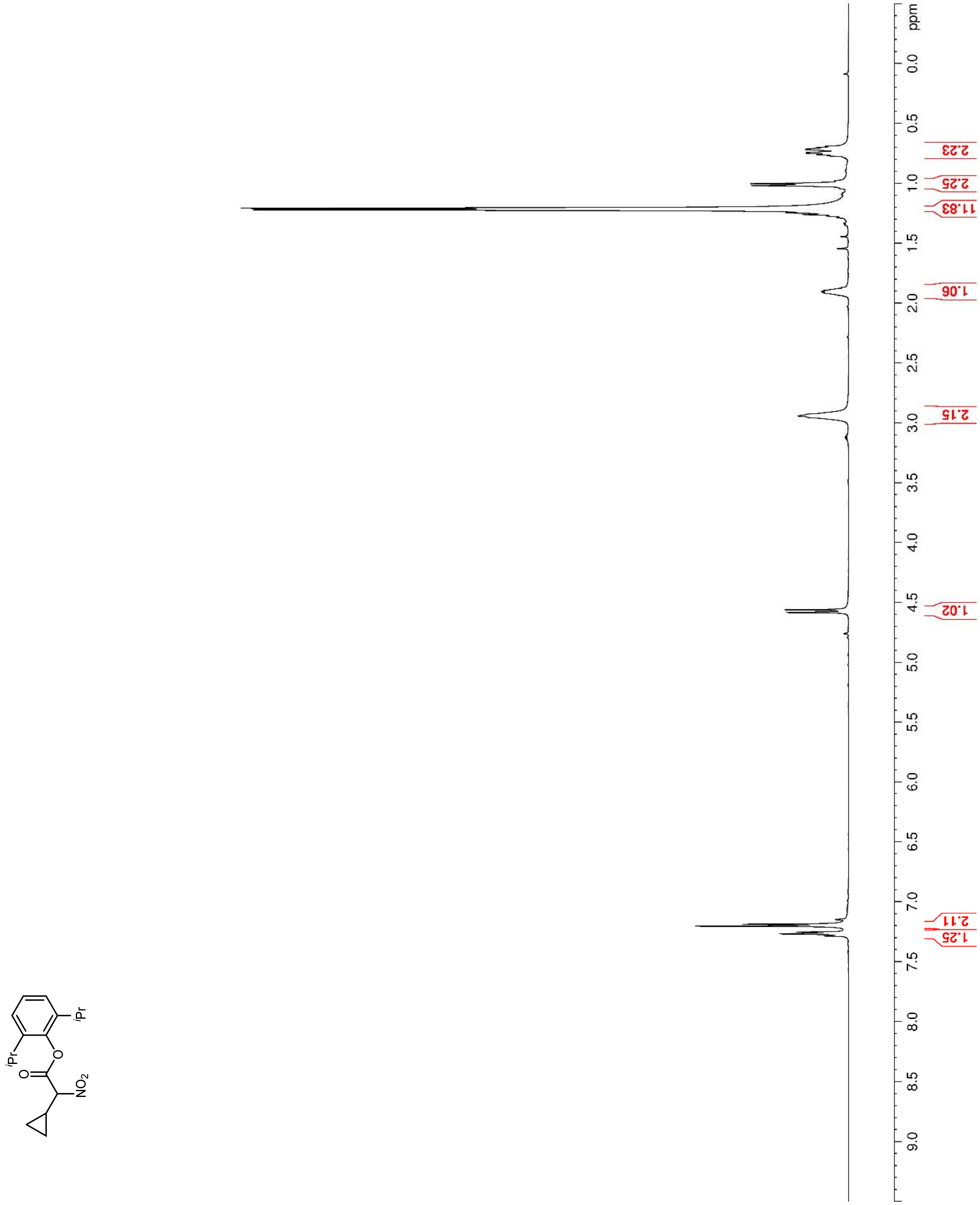

Figure 6. ^{13}C NMR (125 MHz, $CDCl_3$) of **12g**

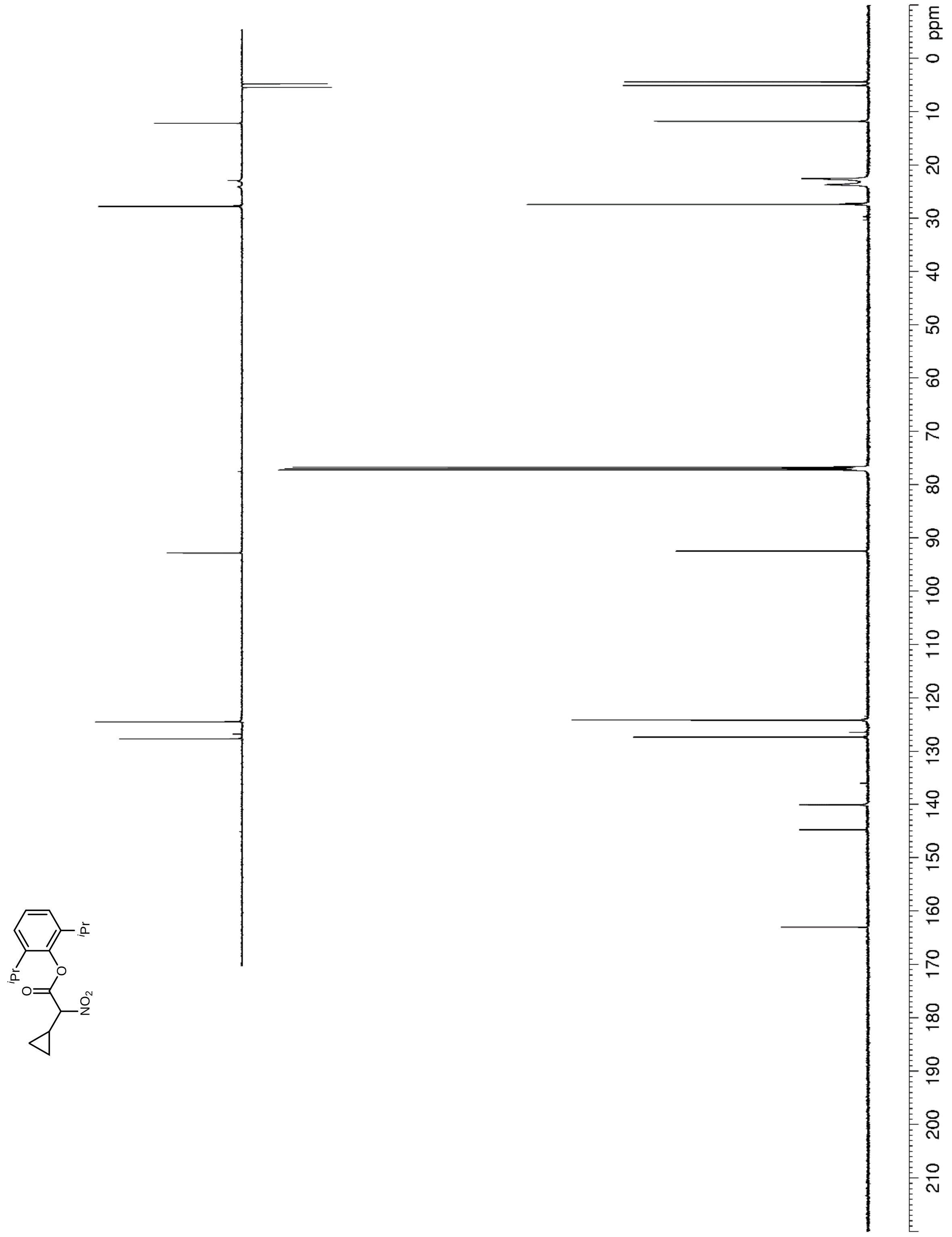

Figure 7. ^{1}H NMR (400 MHz, $CDCl_3$) of **12i**

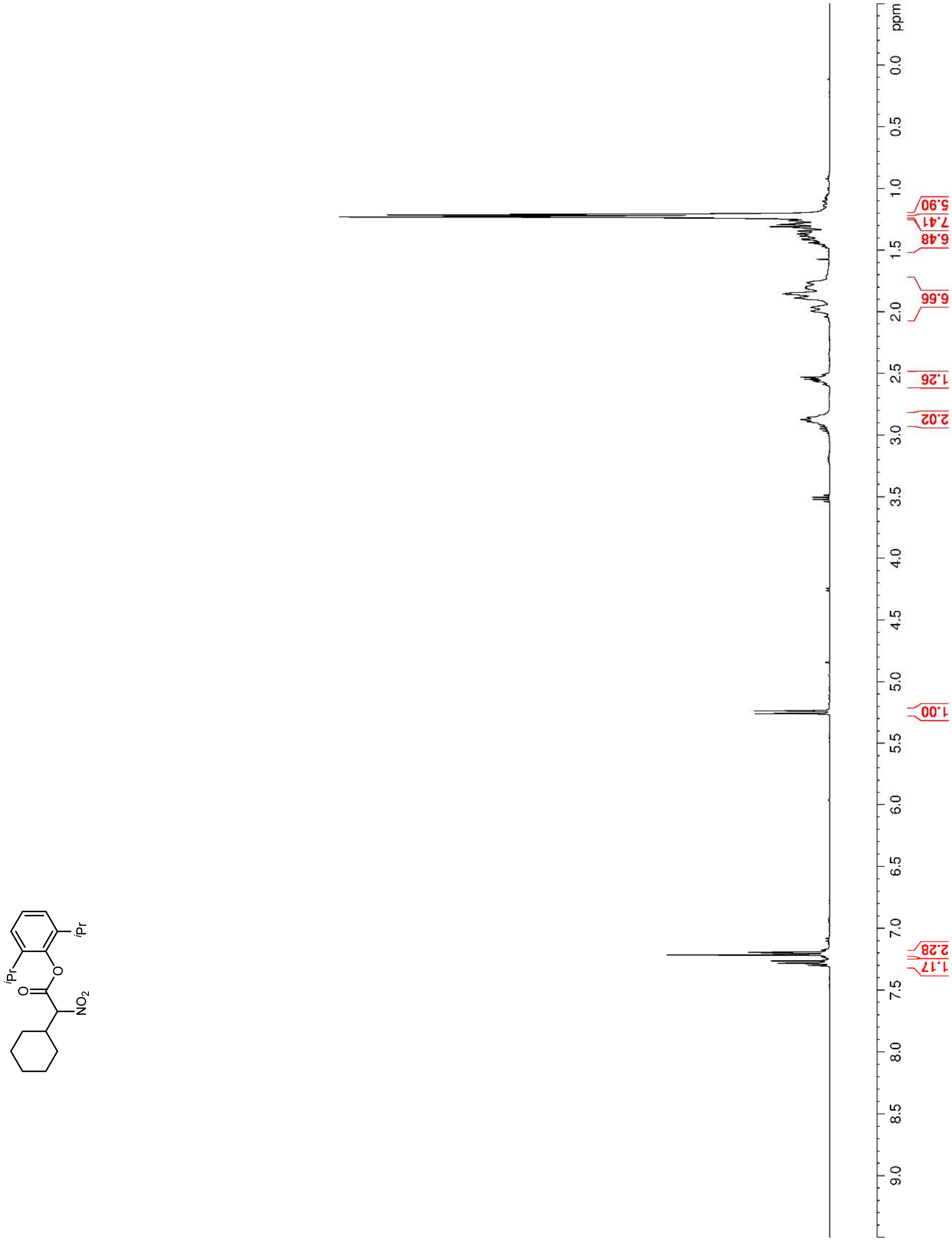

Figure 8. ^{13}C NMR (100 MHz, $CDCl_3$) of **12i**

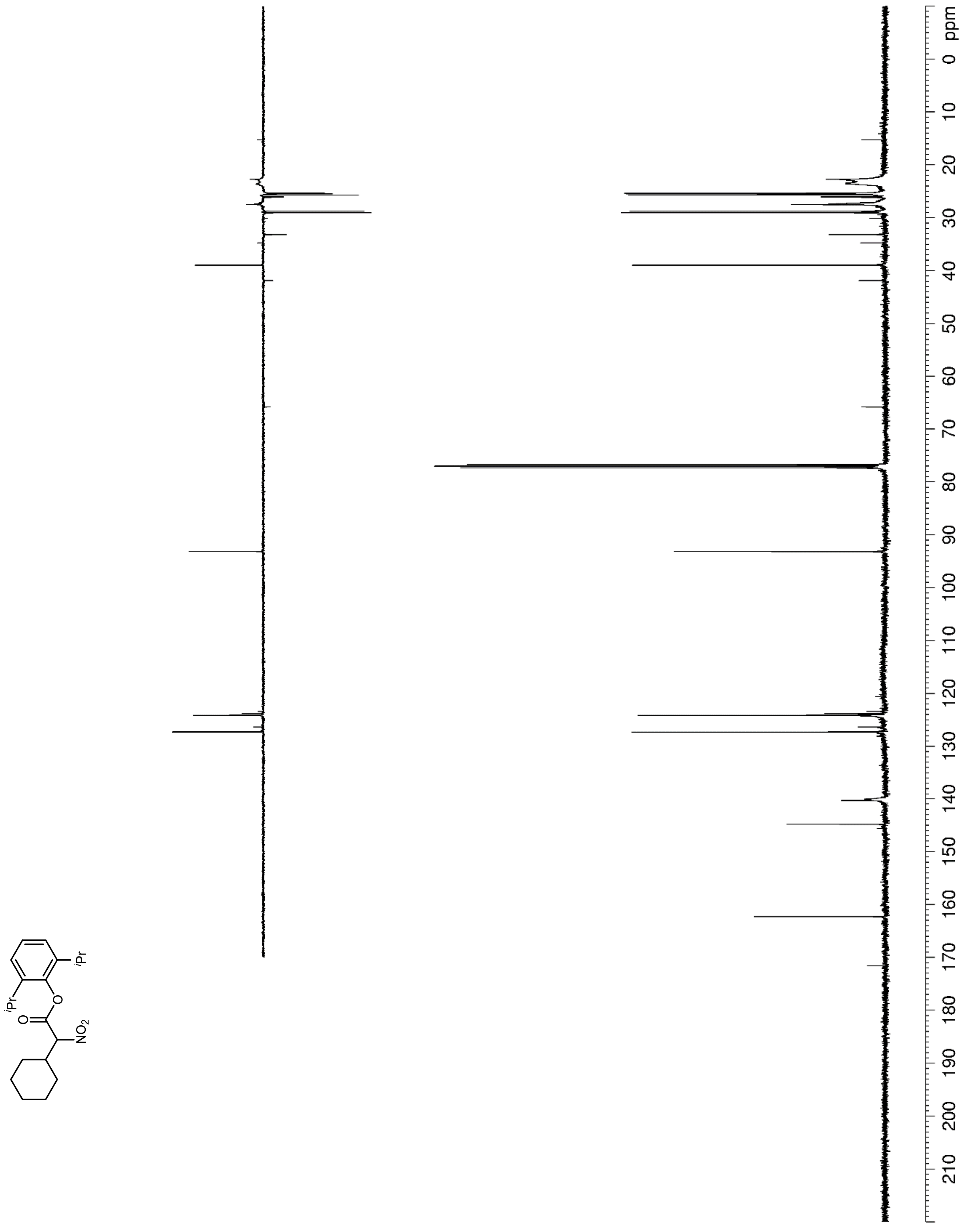

Figure 9. ^{1}H NMR (400 MHz, $CDCl_3$) of **13a**

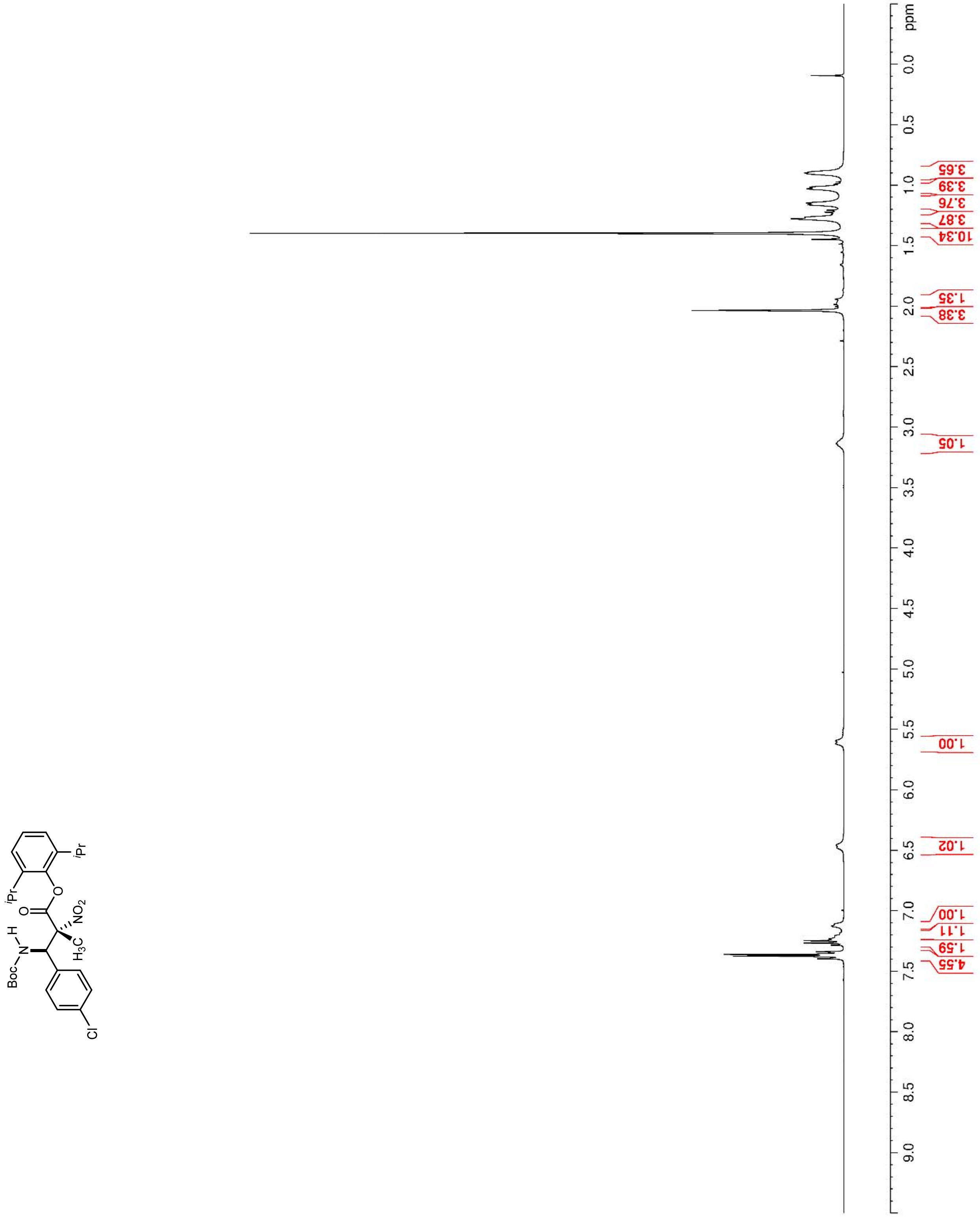

Figure 10. ^{13}C NMR (100 MHz, $CDCl_3$) of **13a**

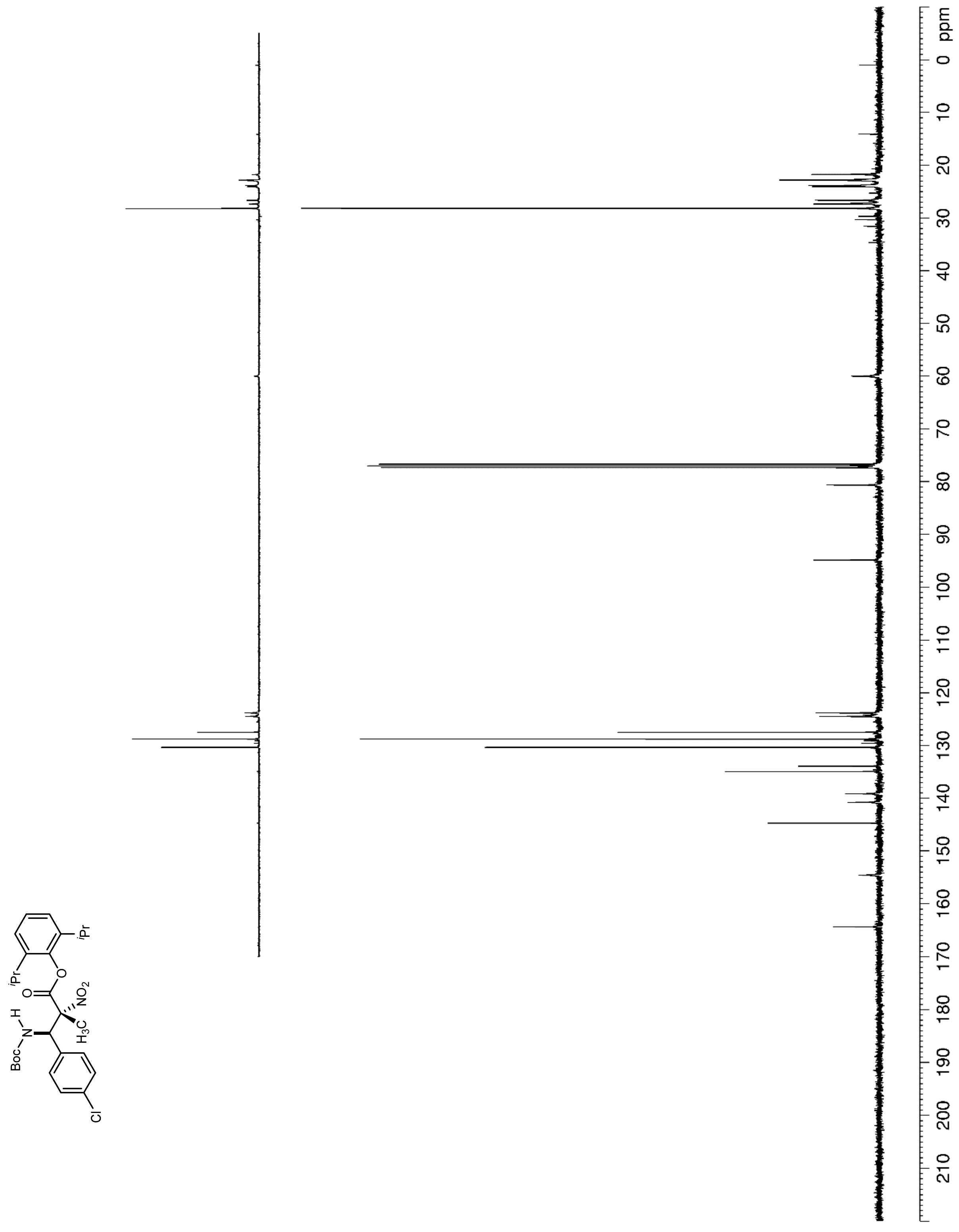

Figure 11. ^{1}H NMR (400 MHz, $CDCl_3$) of **13b**

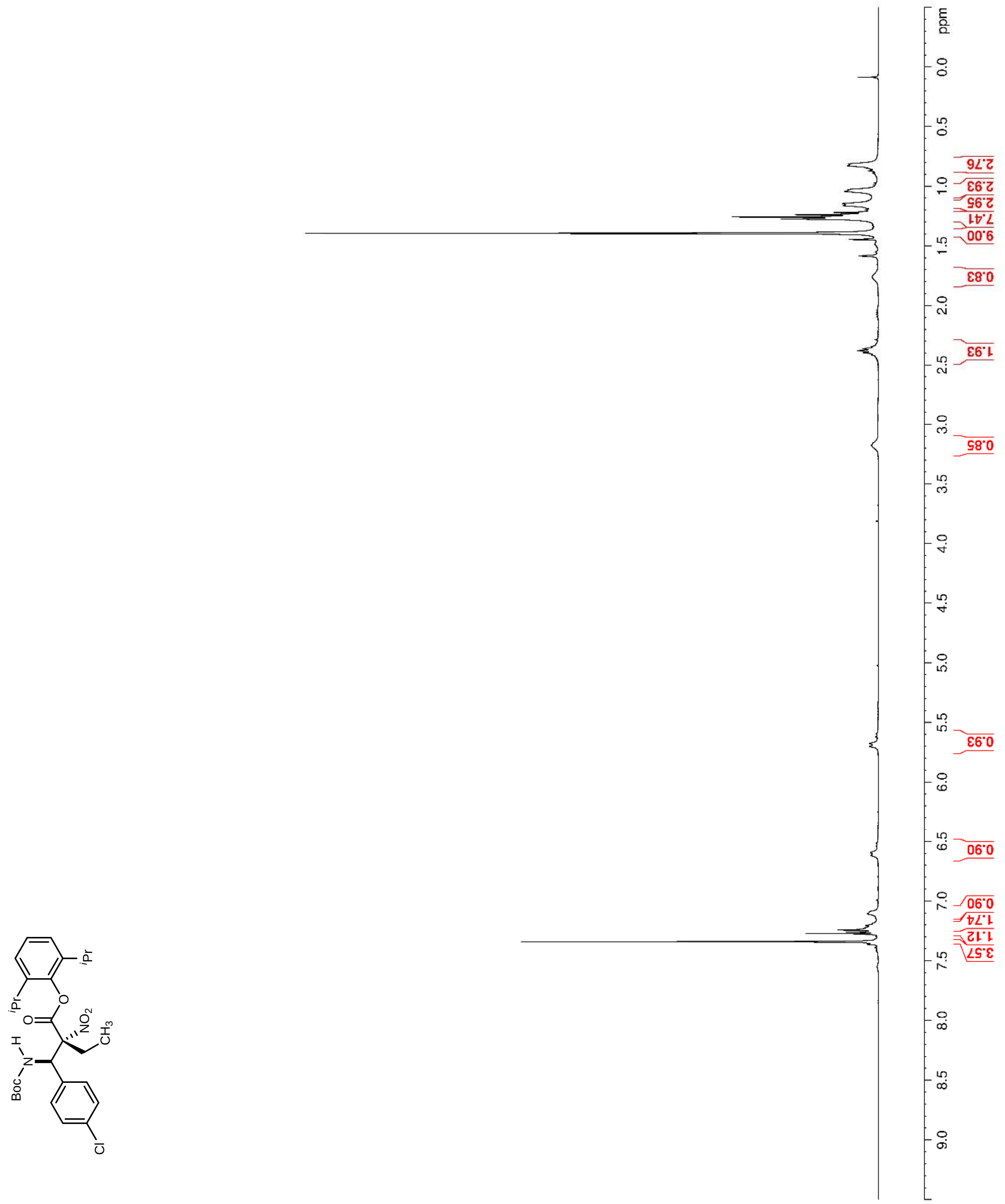

Figure 12. ^{13}C NMR (100 MHz, $CDCl_3$) of **13b**

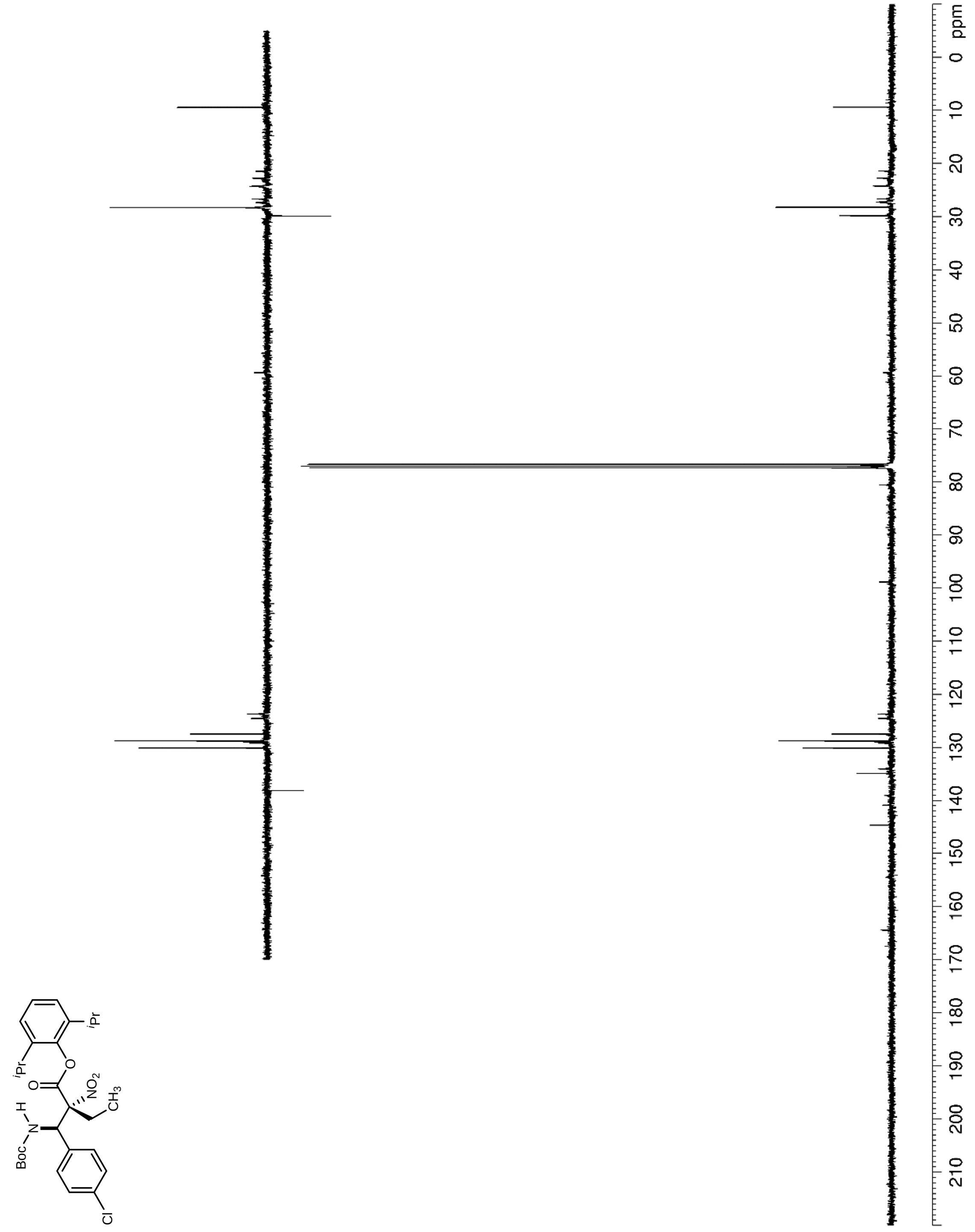

Figure 13. ^{1}H NMR (400 MHz, $CDCl_3$) of **13c**

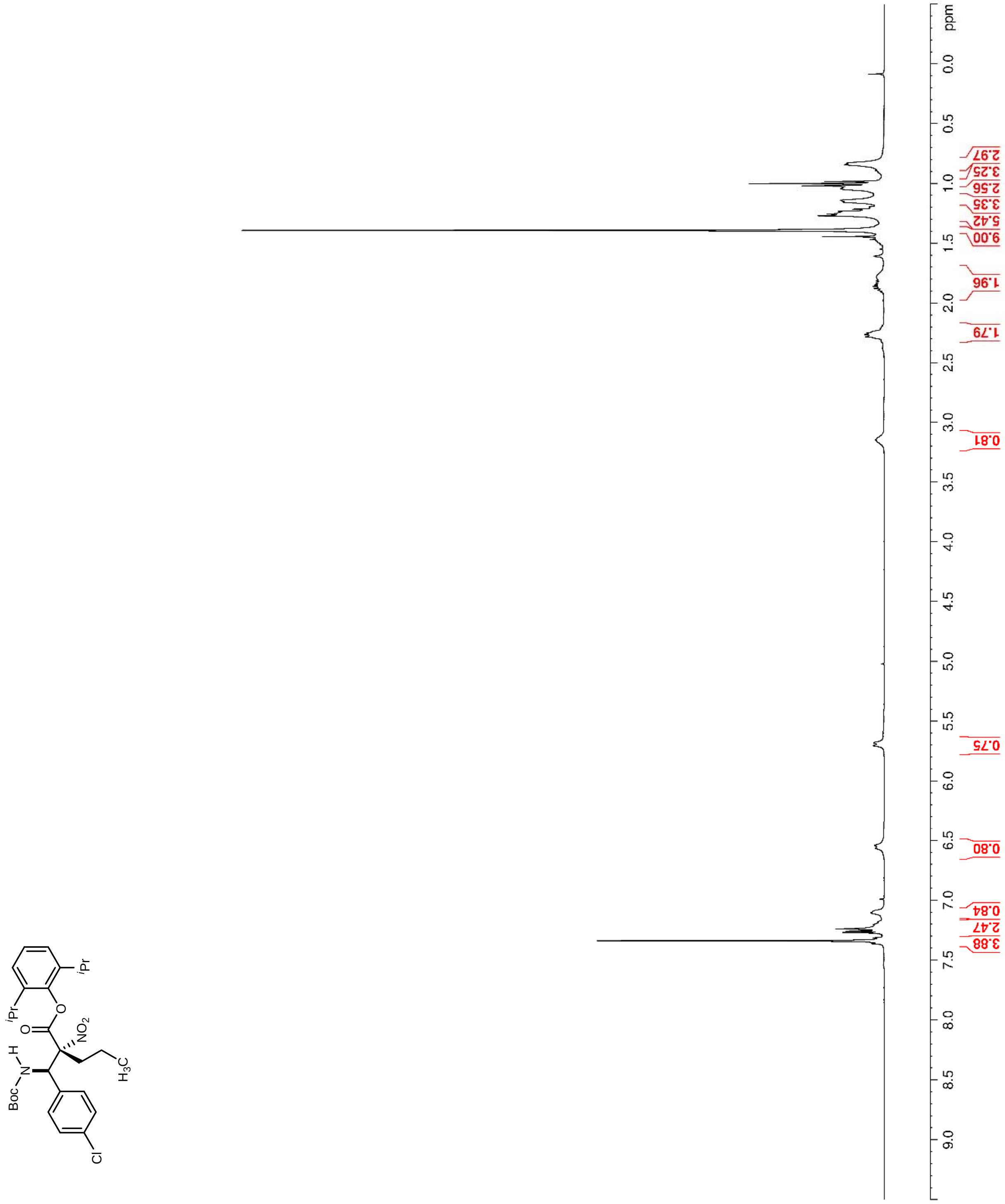

Figure 14. ^{13}C NMR (100 MHz, $CDCl_3$) of **13c**

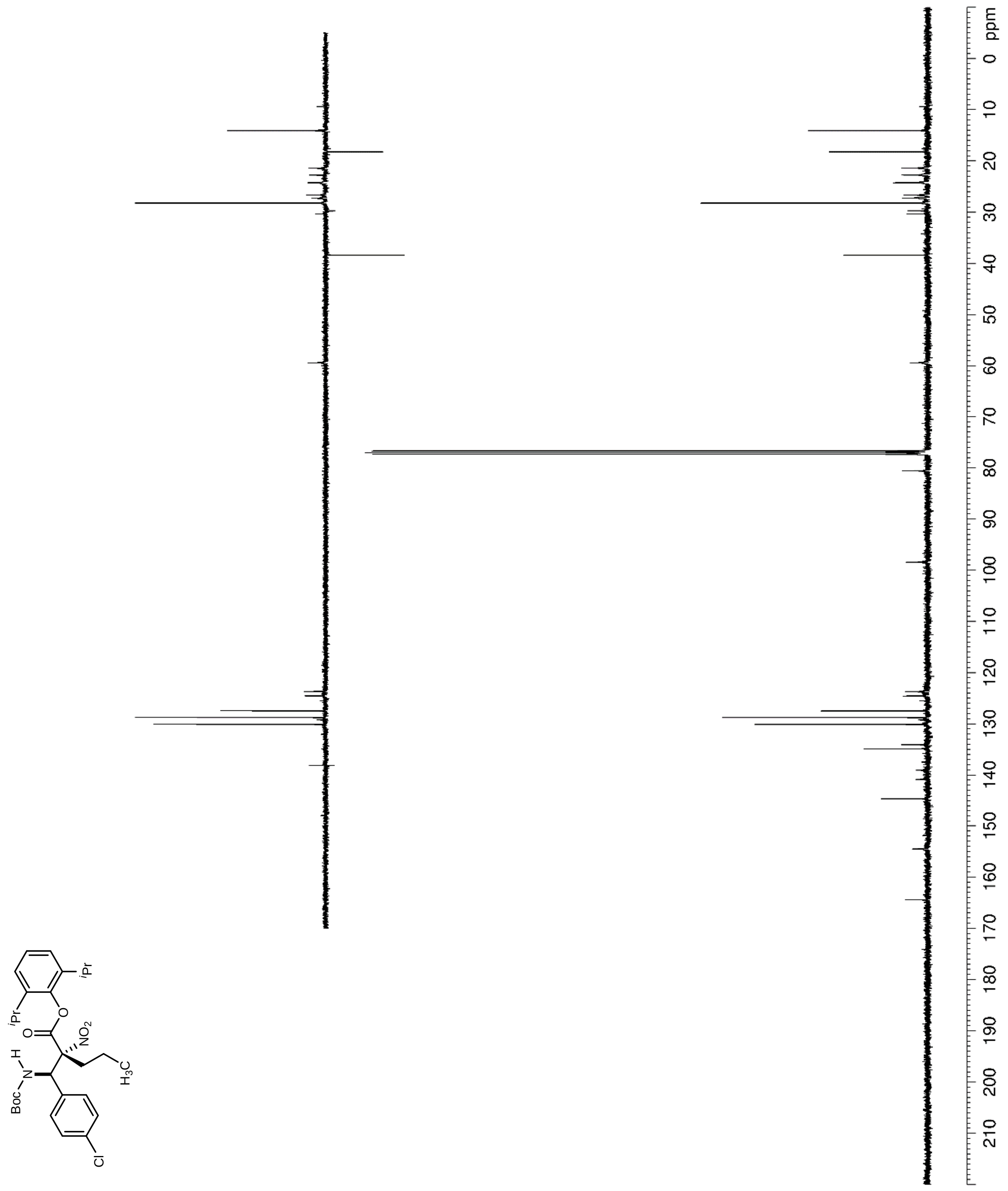

Figure 15. ^{1}H NMR (400 MHz, $CDCl_3$) of **13d**

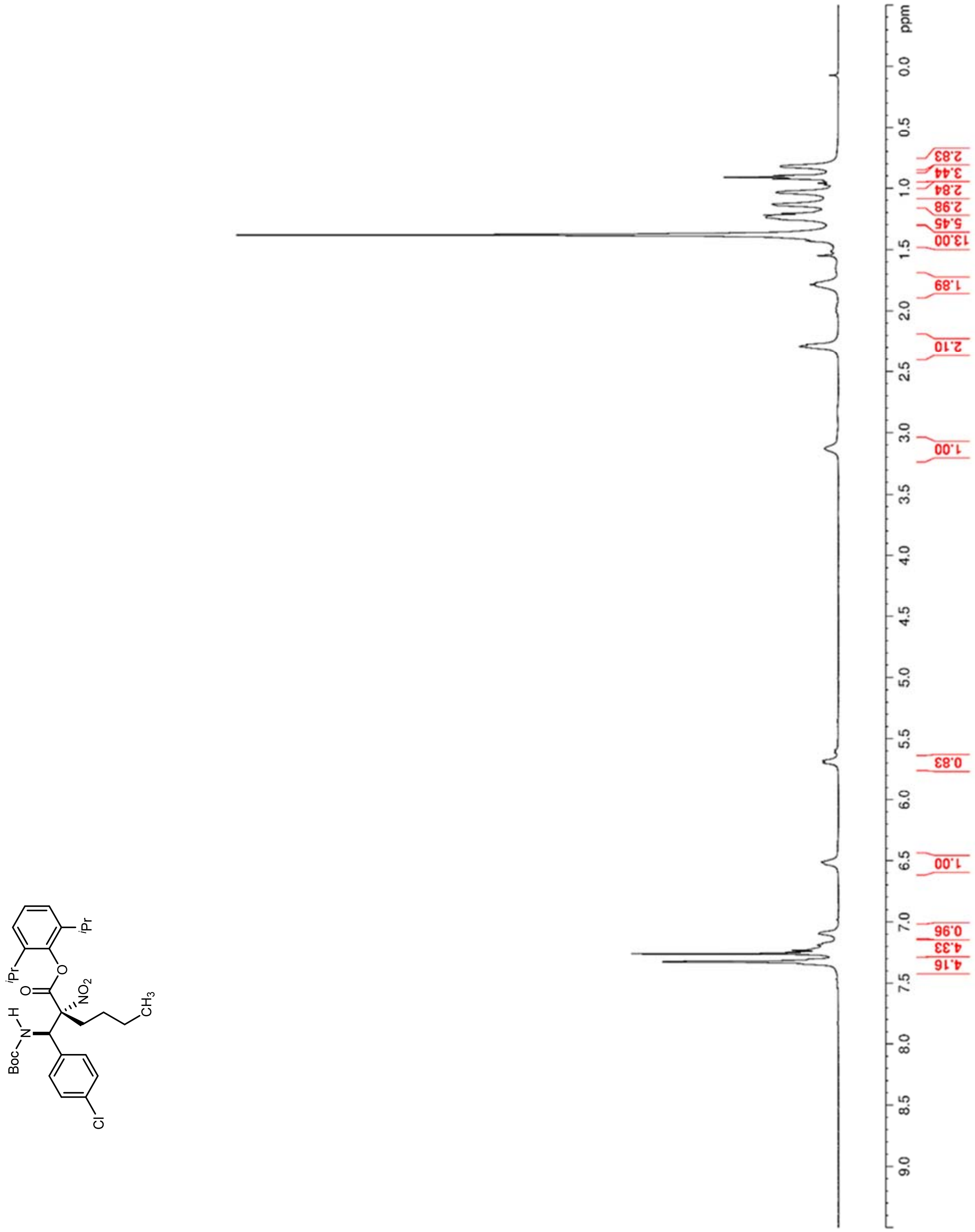

Figure 16. ^{13}C NMR (100 MHz, $CDCl_3$) of **13d**

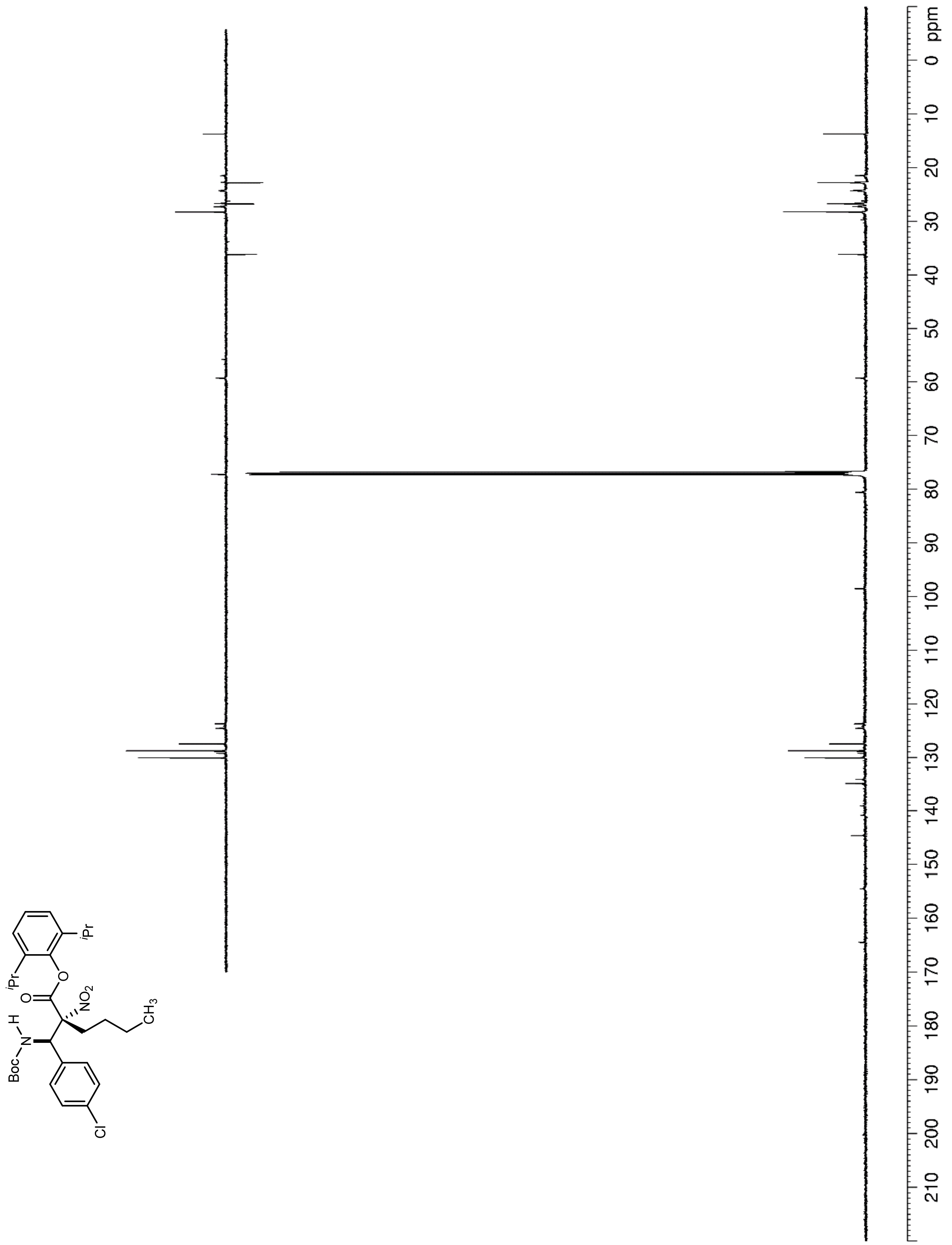

Figure 17. ^{1}H NMR (400 MHz, $CDCl_3$) of **13e**

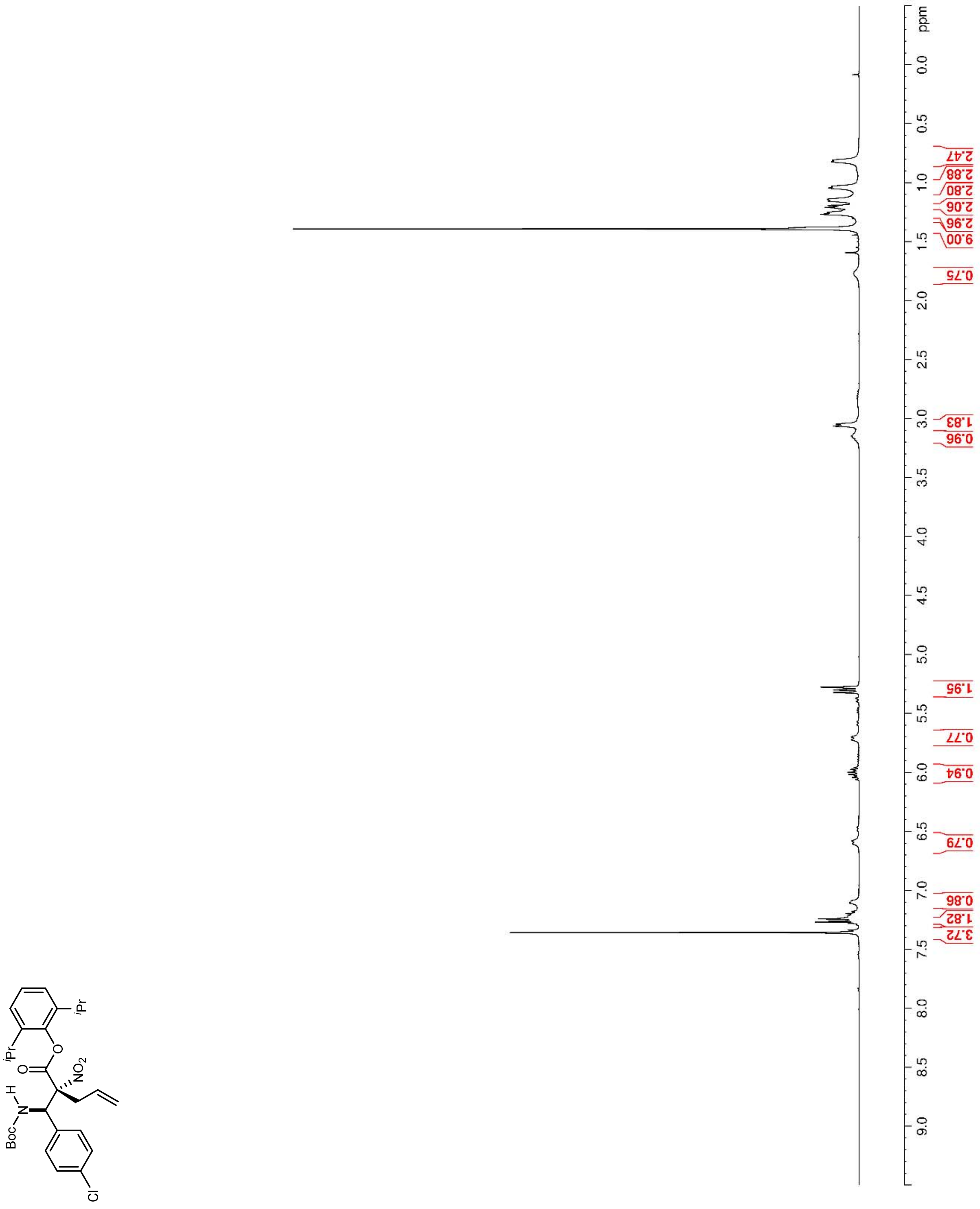

Figure 18. ^{13}C NMR (100 MHz, $CDCl_3$) of **13e**

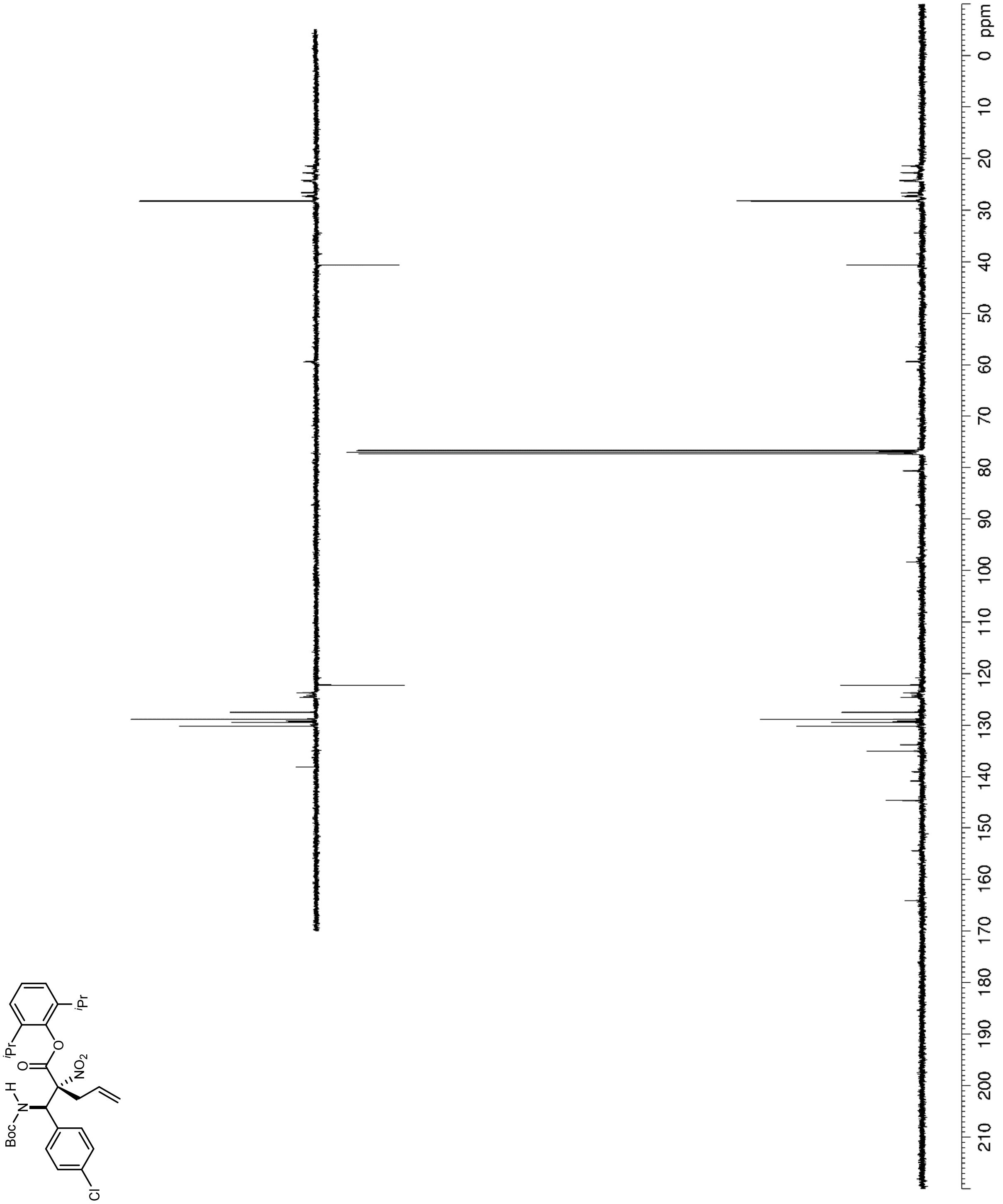

Figure 19. ^{1}H NMR (400 MHz, $CDCl_3$) of **13f**

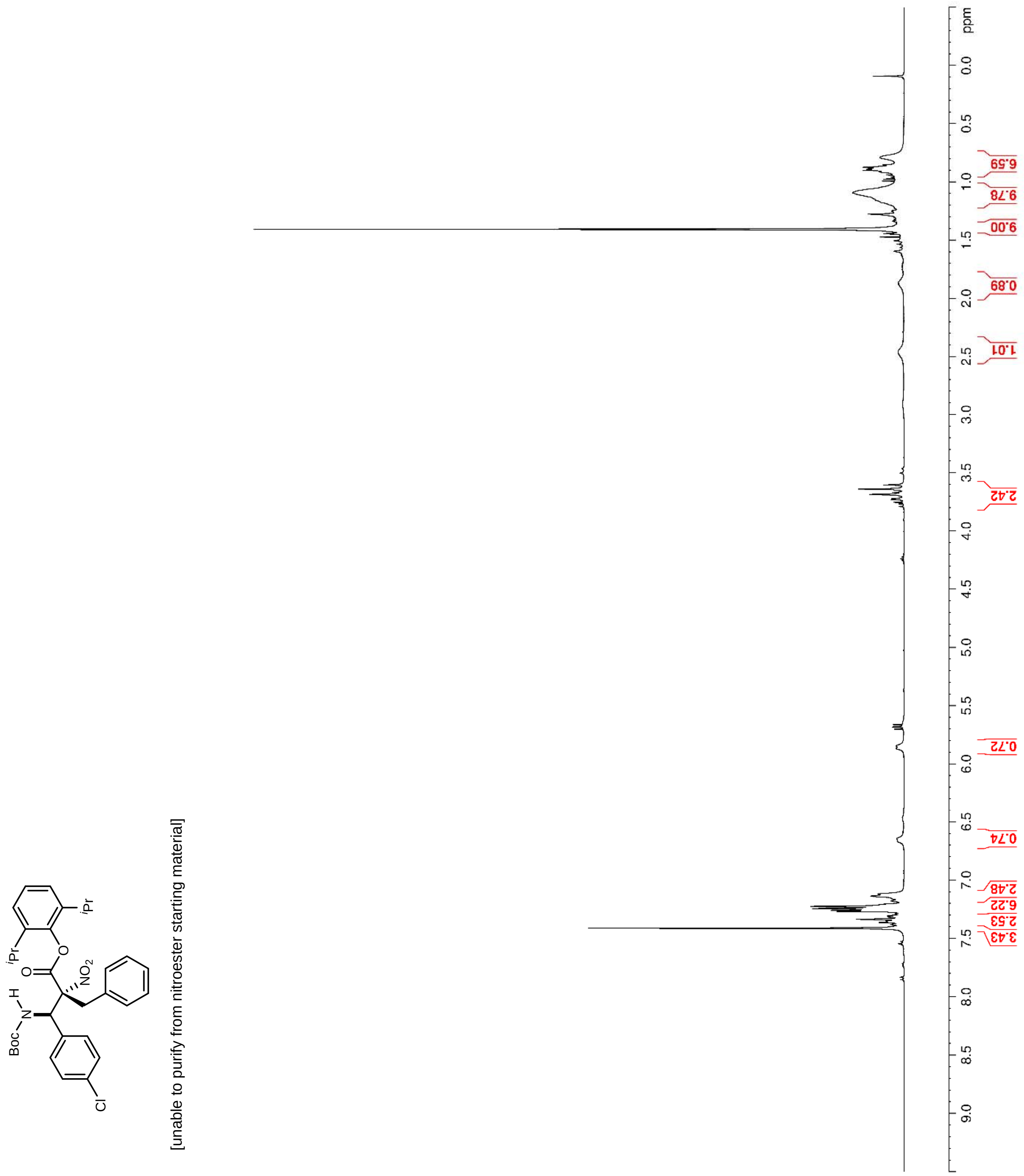

Figure 20. ^{13}C NMR (100 MHz, $CDCl_3$) of **13f**

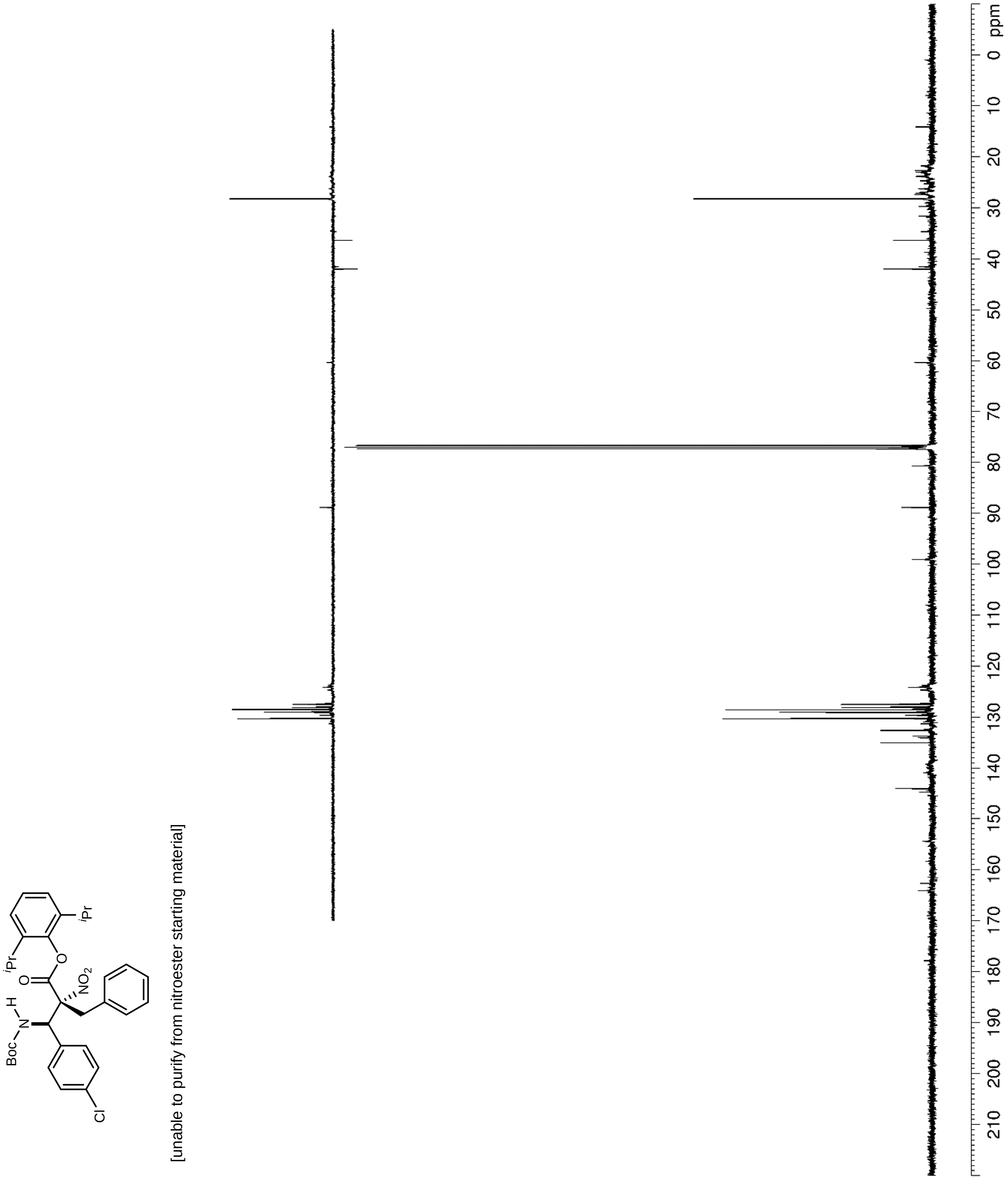

Figure 21. ^{1}H NMR (400 MHz, $CDCl_3$) of **13g**

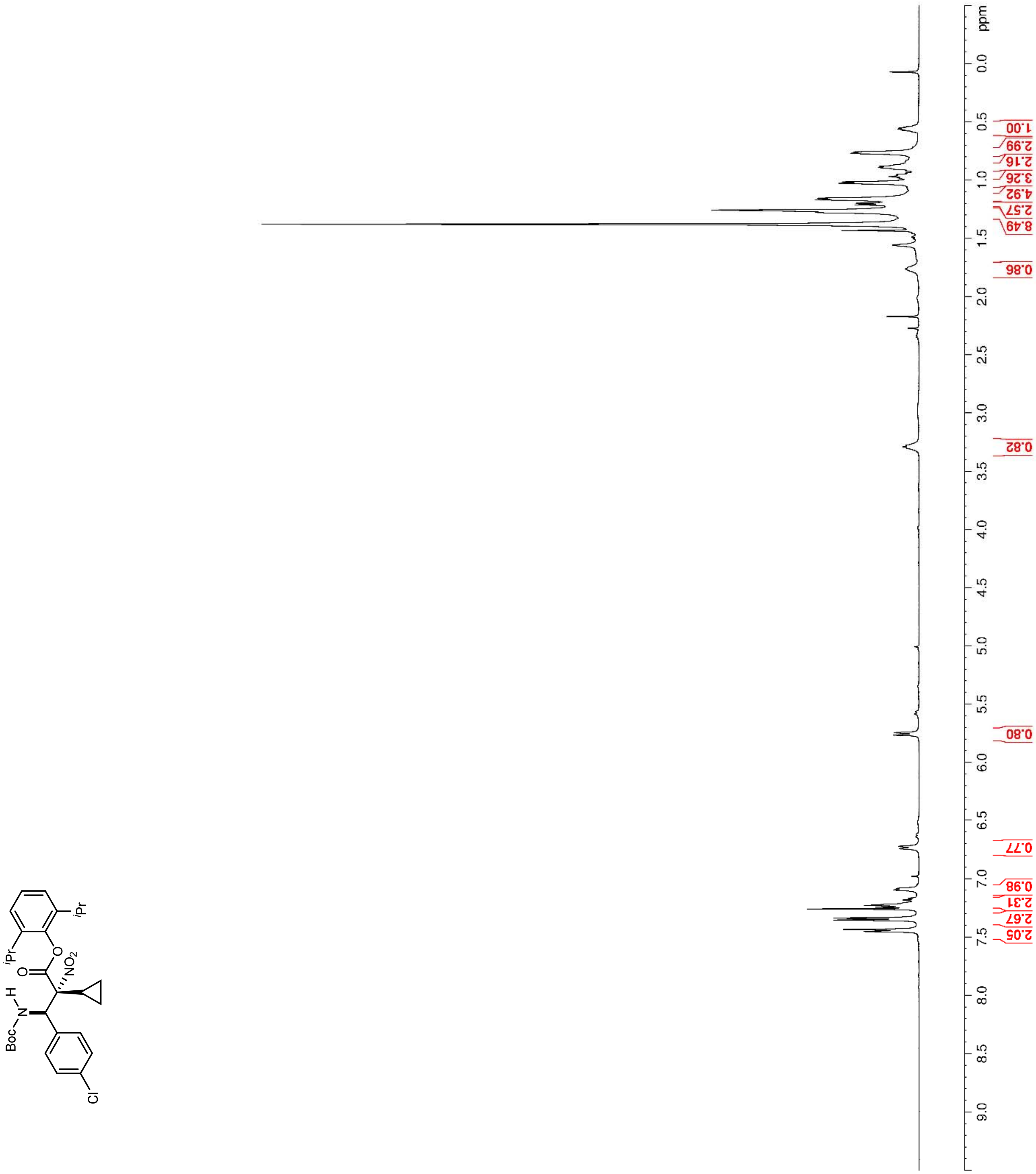

Figure 22. ^{13}C NMR (100 MHz, $CDCl_3$) of **13g**

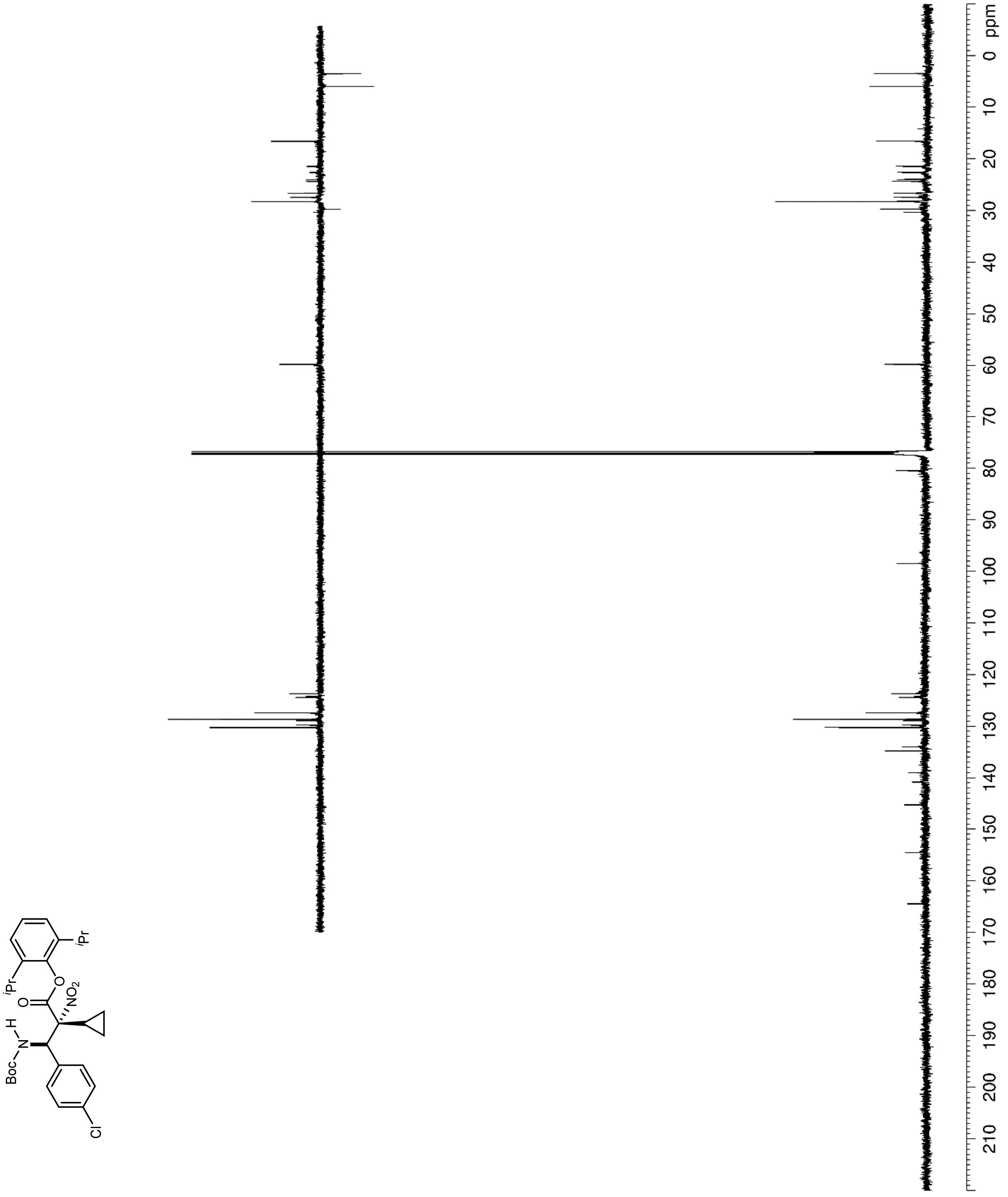

Figure 23. ^{1}H NMR (400 MHz, $CDCl_3$) of **13h**

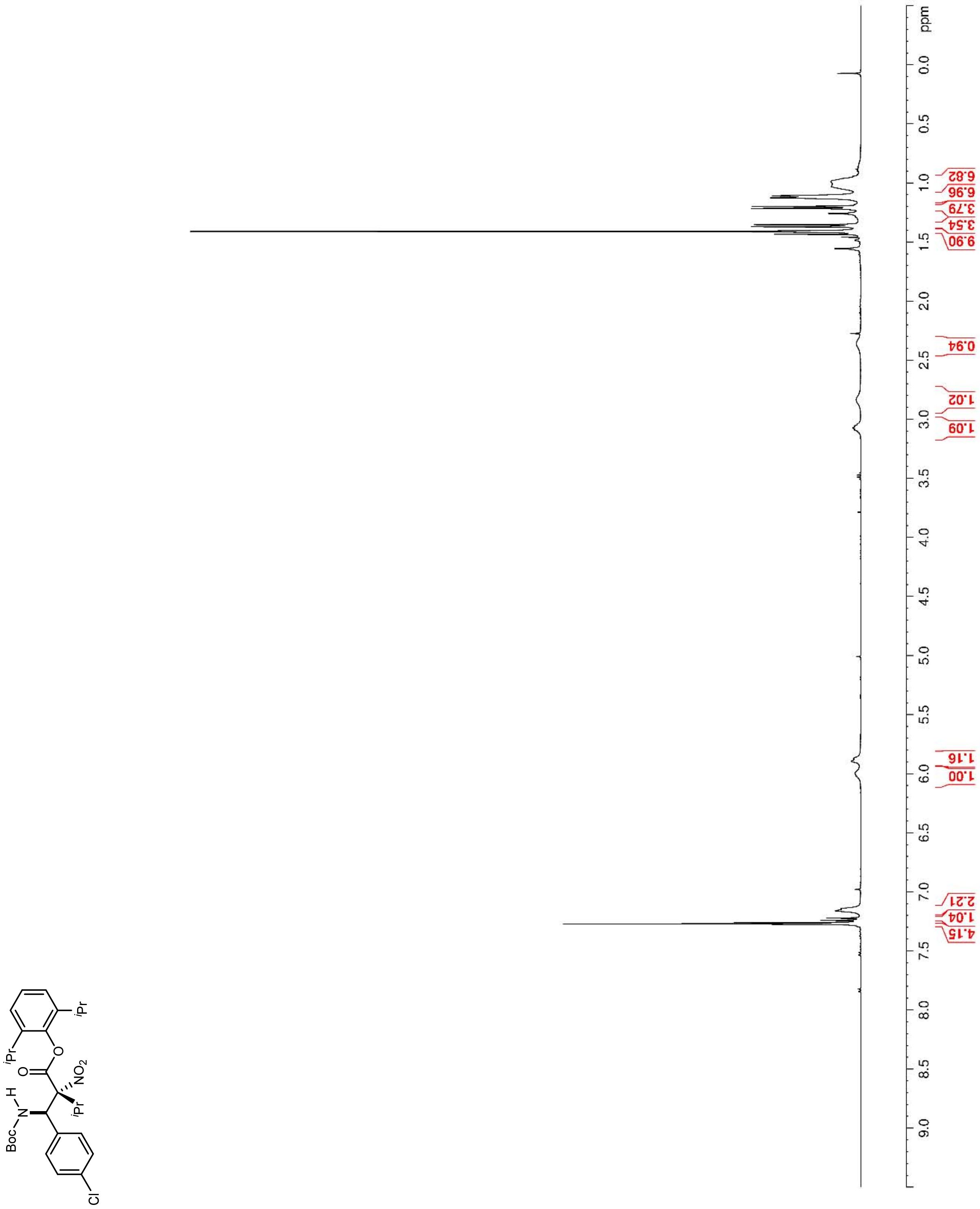

Figure 24. ^{13}C NMR (100 MHz, $CDCl_3$) of **13h**

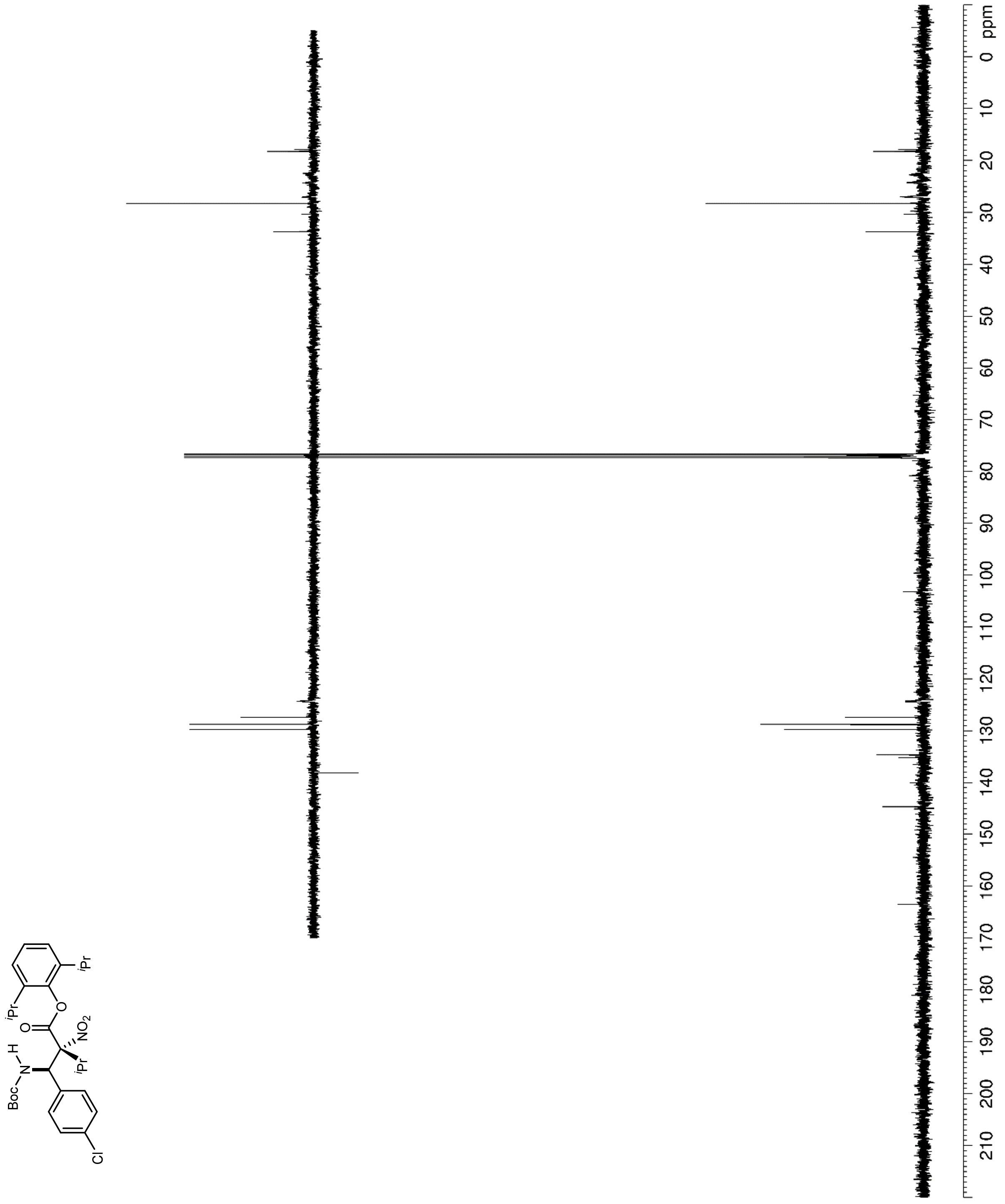

Figure 25. ^{1}H NMR (400 MHz, $CDCl_3$) of **13i**

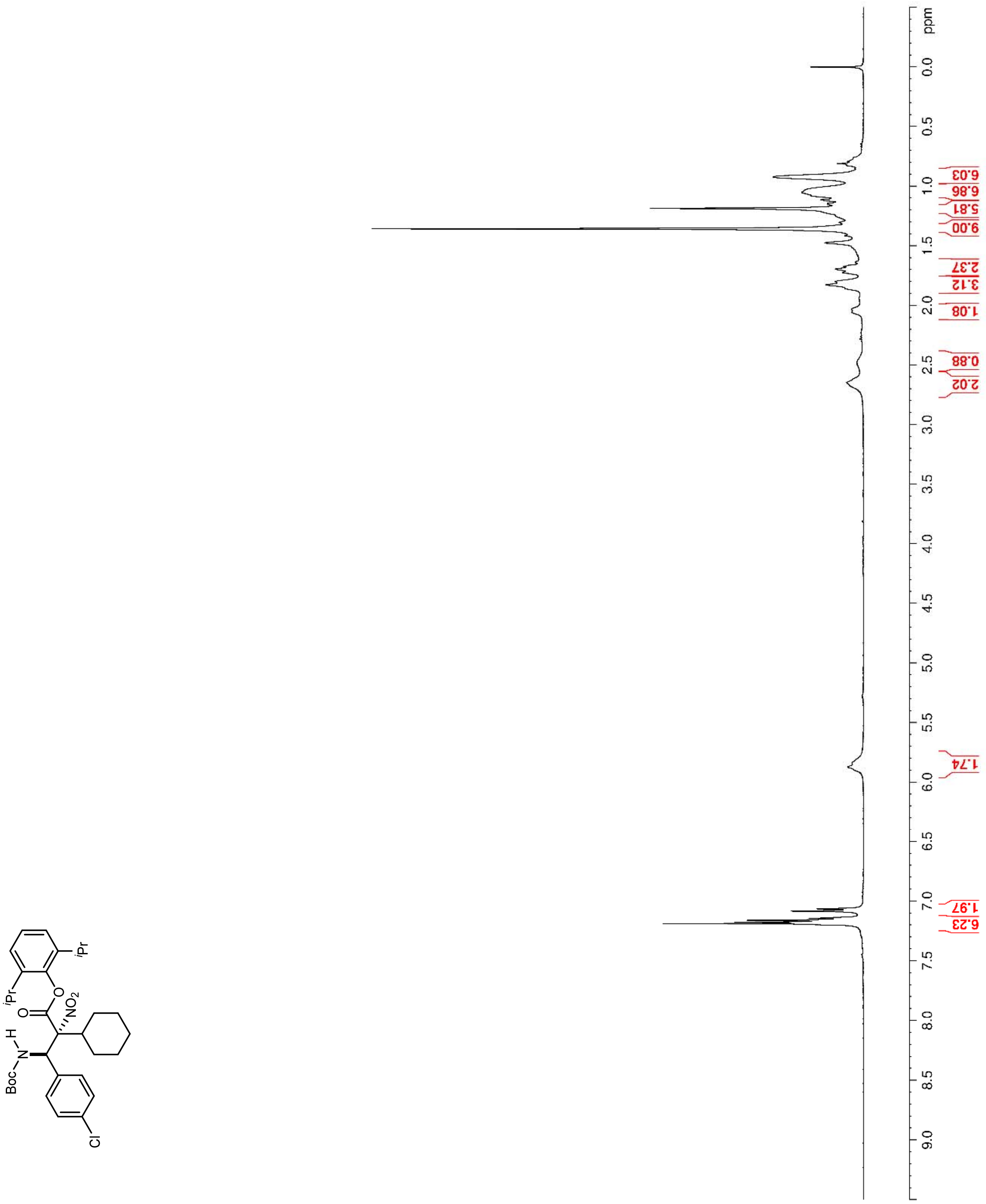

Figure 26. ^{13}C NMR (100 MHz, $CDCl_3$) of **13i**

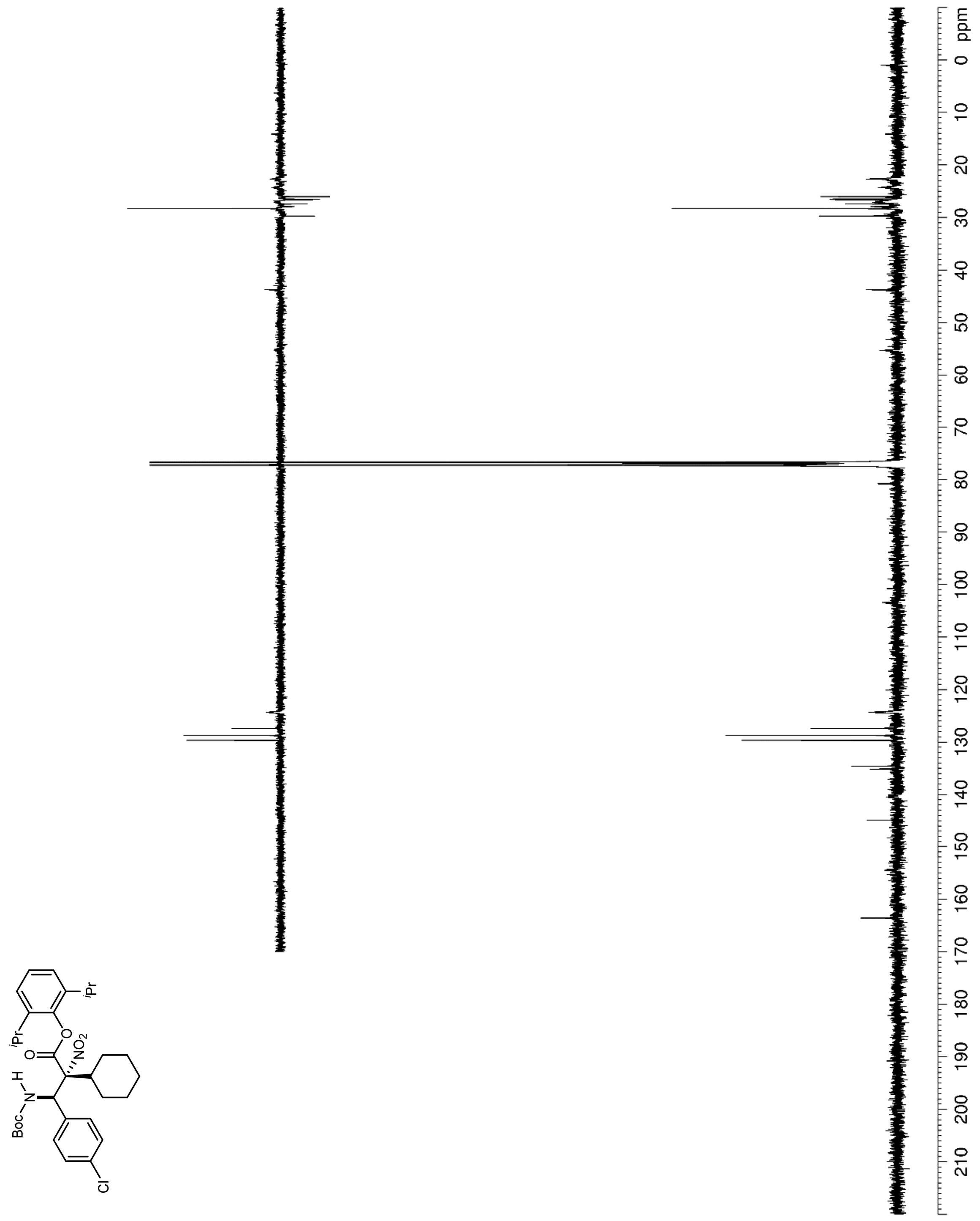

Figure 27. ^{1}H NMR (500 MHz, DMSO-d_6) of **13j**

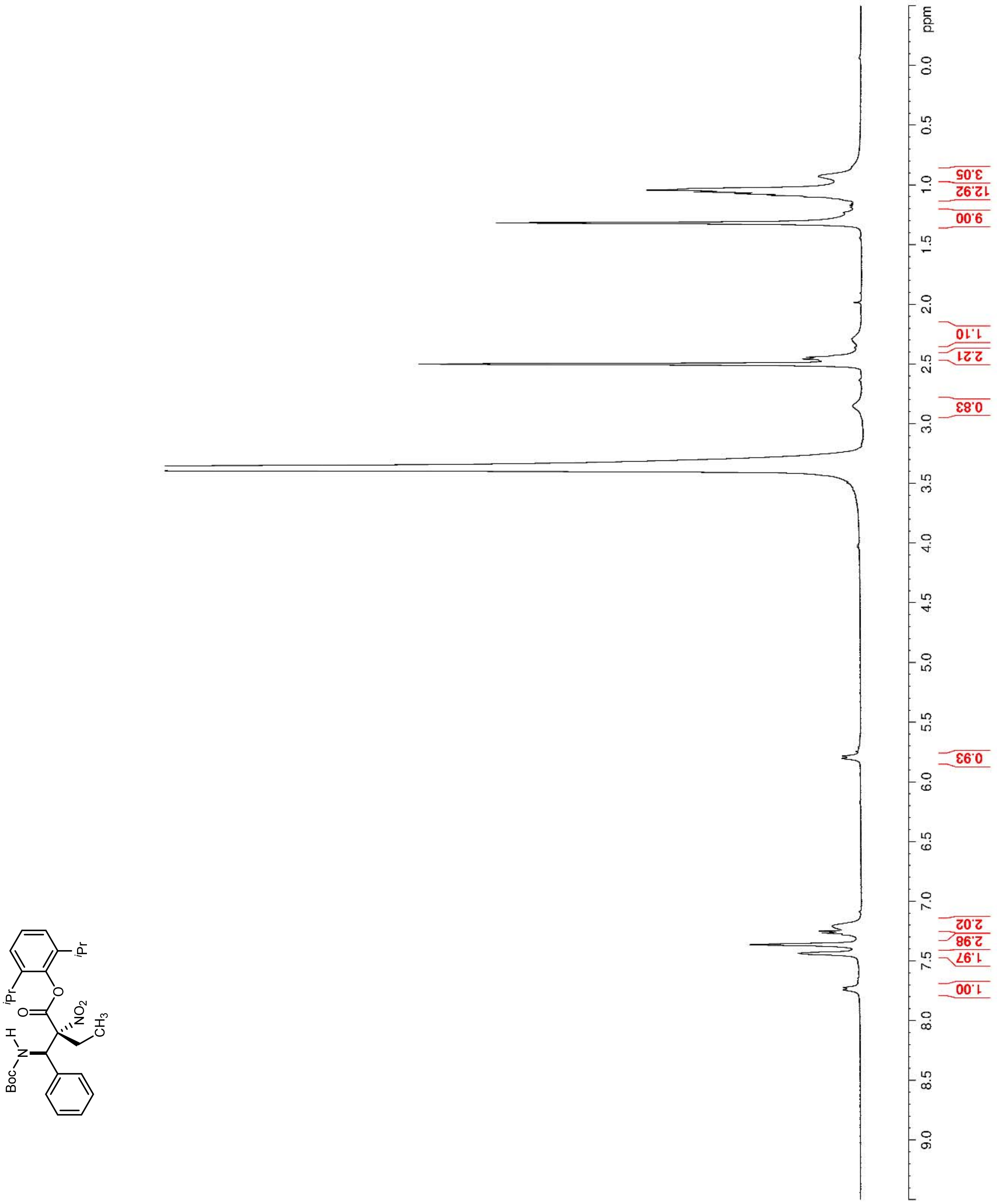

Figure 28. ^{13}C NMR (125 MHz, DMSO-d_6) of **13j**

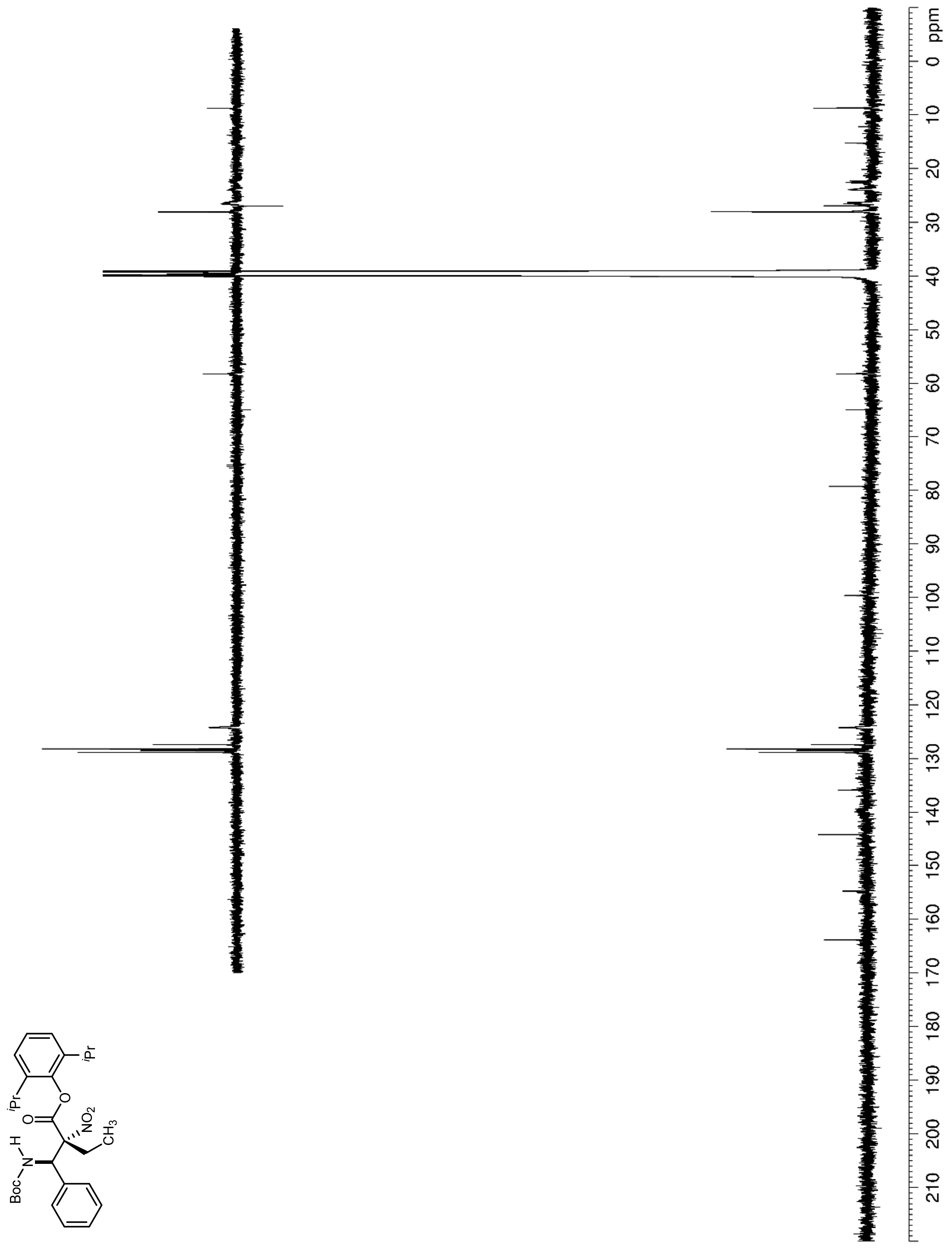

Figure 29. ^{1}H NMR (400 MHz, $CDCl_3$) of **13k**

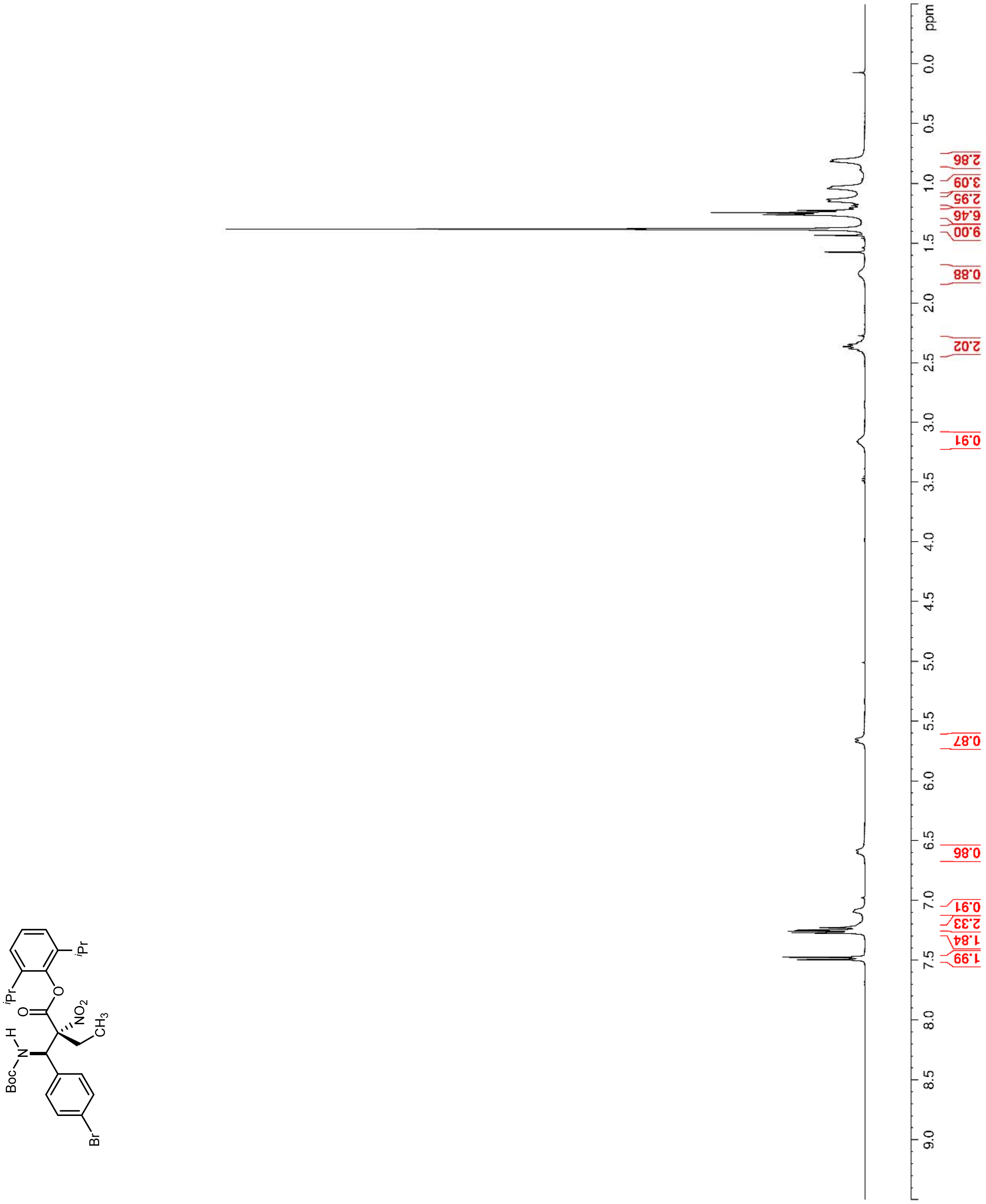

Figure 30. ^{13}C NMR (100 MHz, $CDCl_3$) of **13k**

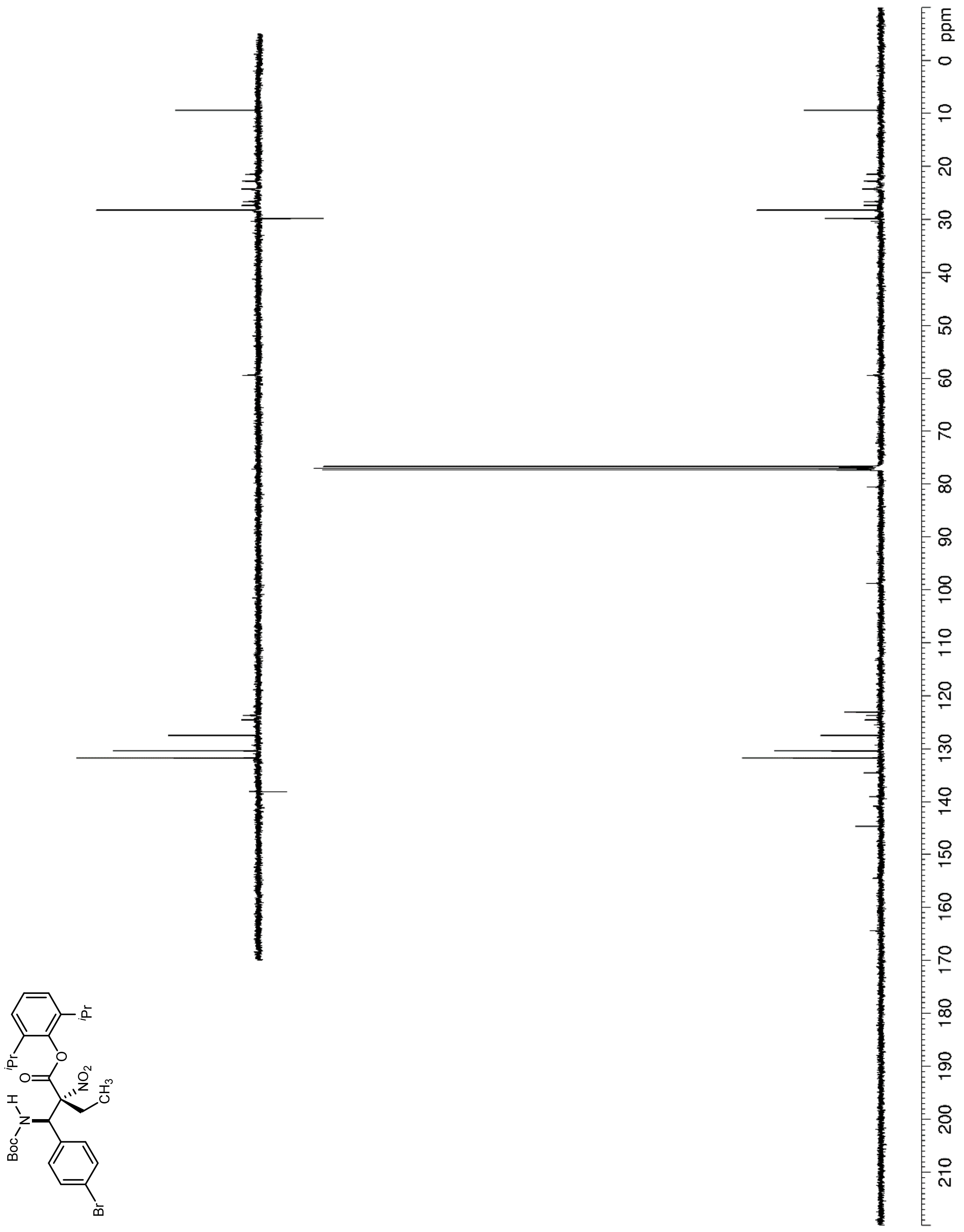

Figure 31. ^{1}H NMR (400 MHz, $CDCl_3$) of **13l**

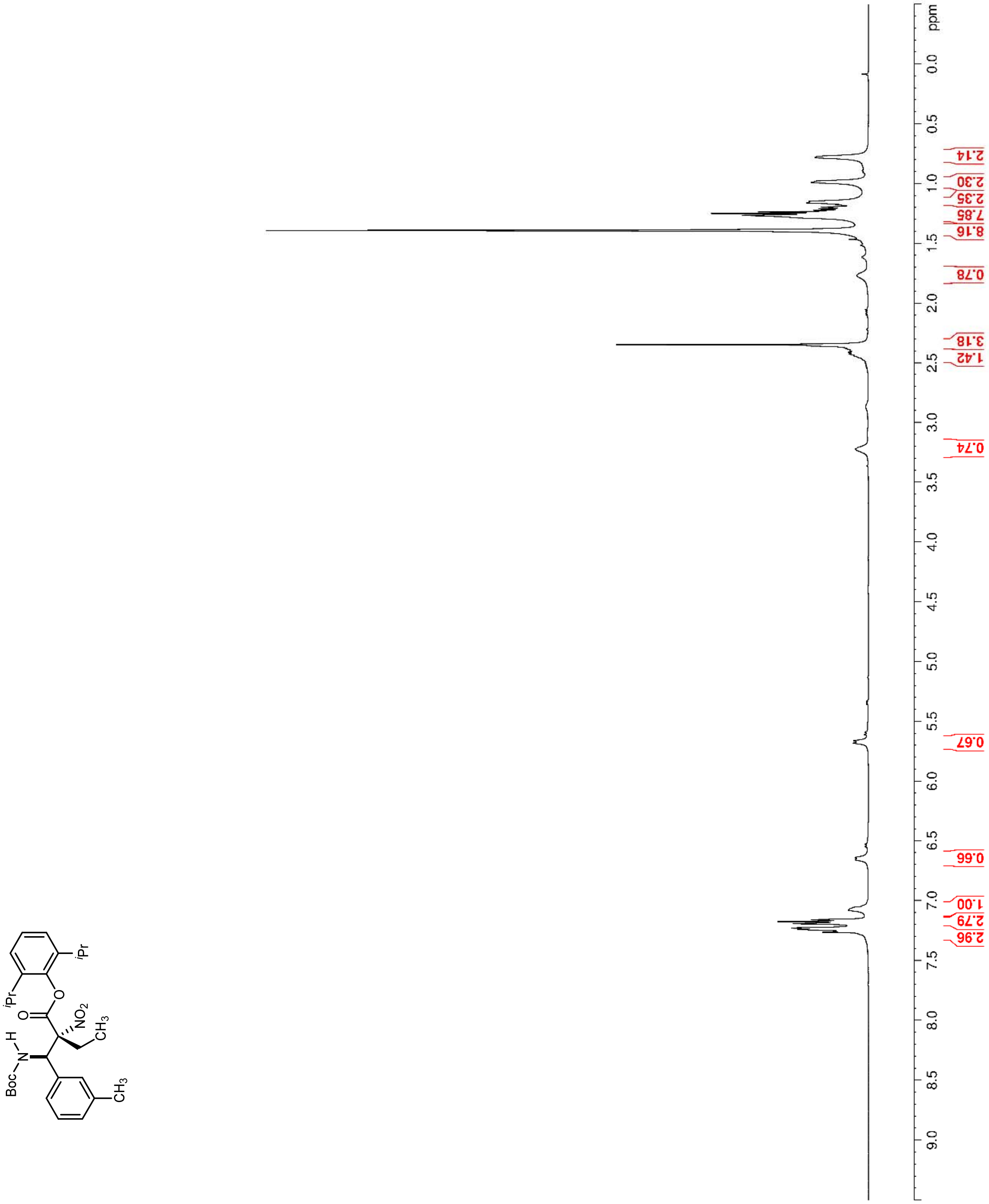

Figure 32. ^{13}C NMR (100 MHz, $CDCl_3$) of **13l**

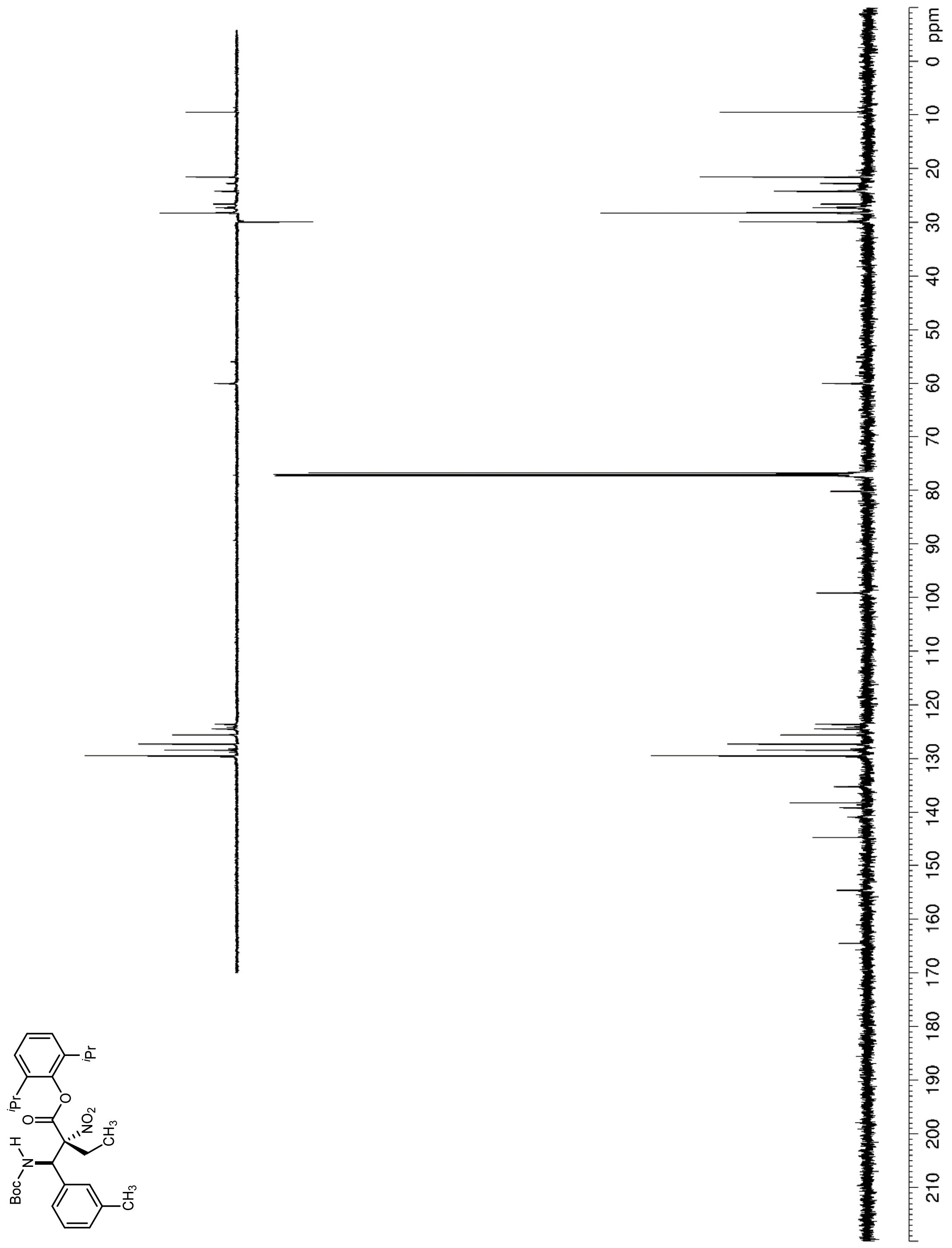

Figure 33. ^{1}H NMR (400 MHz, $CDCl_3$) of **13m**

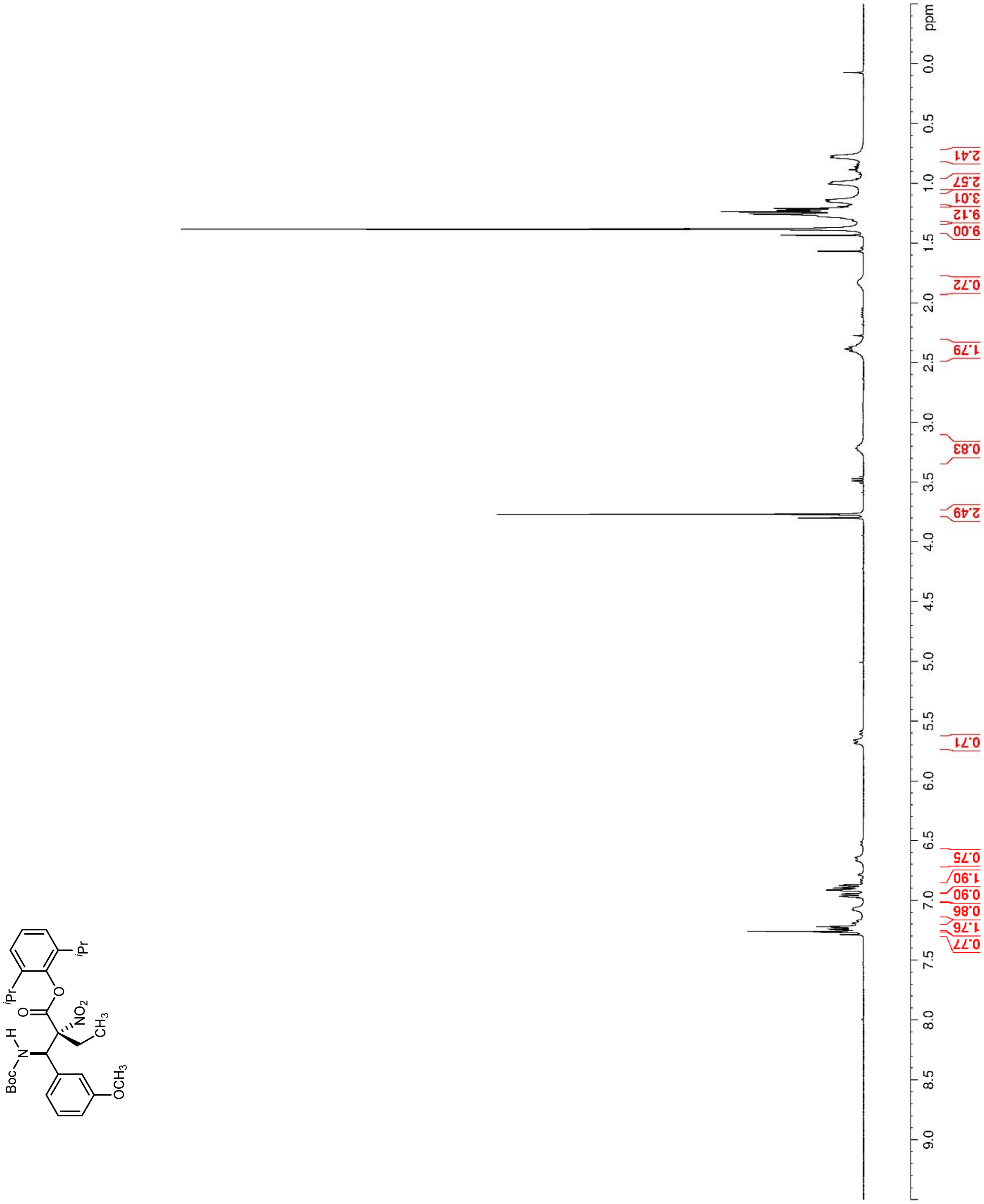

Figure 34. ^{13}C NMR (100 MHz, $CDCl_3$) of **13m**

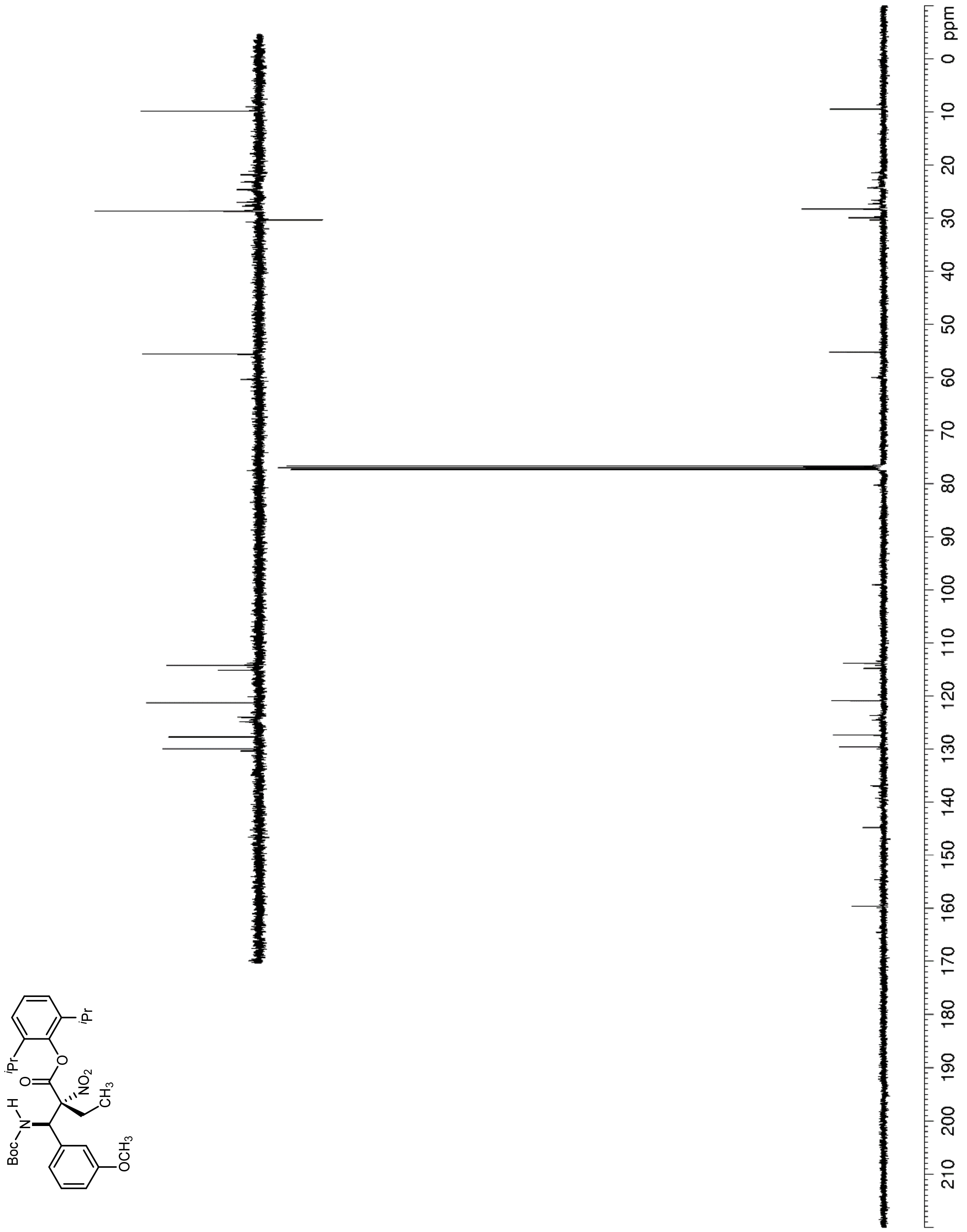

Figure 35. ^{1}H NMR (400 MHz, $CDCl_3$) of **13o**

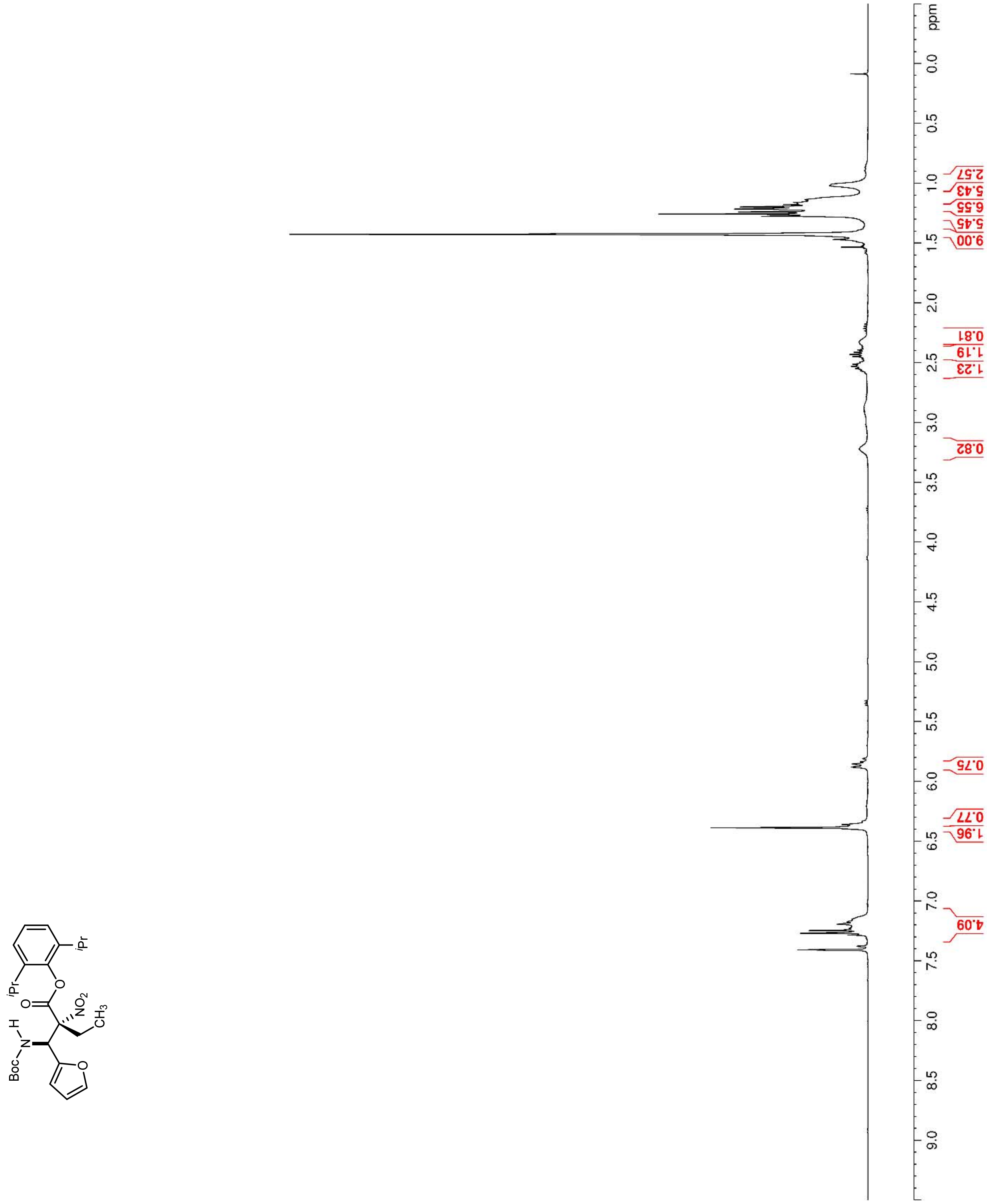

Figure 36. ^{13}C NMR (100 MHz, $CDCl_3$) of **13o**

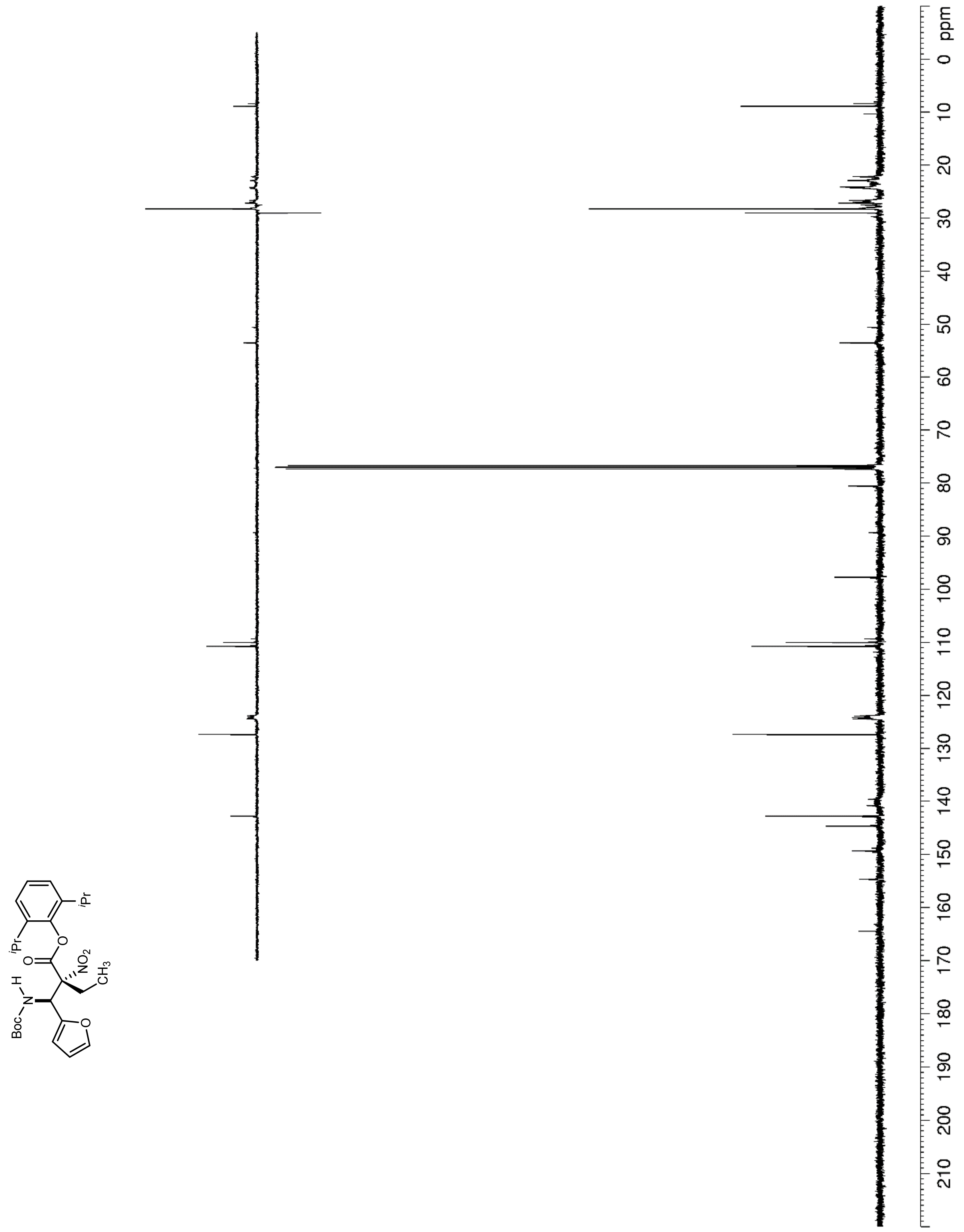

Figure 37. ^{1}H NMR (400 MHz, $CDCl_3$) of **13p**

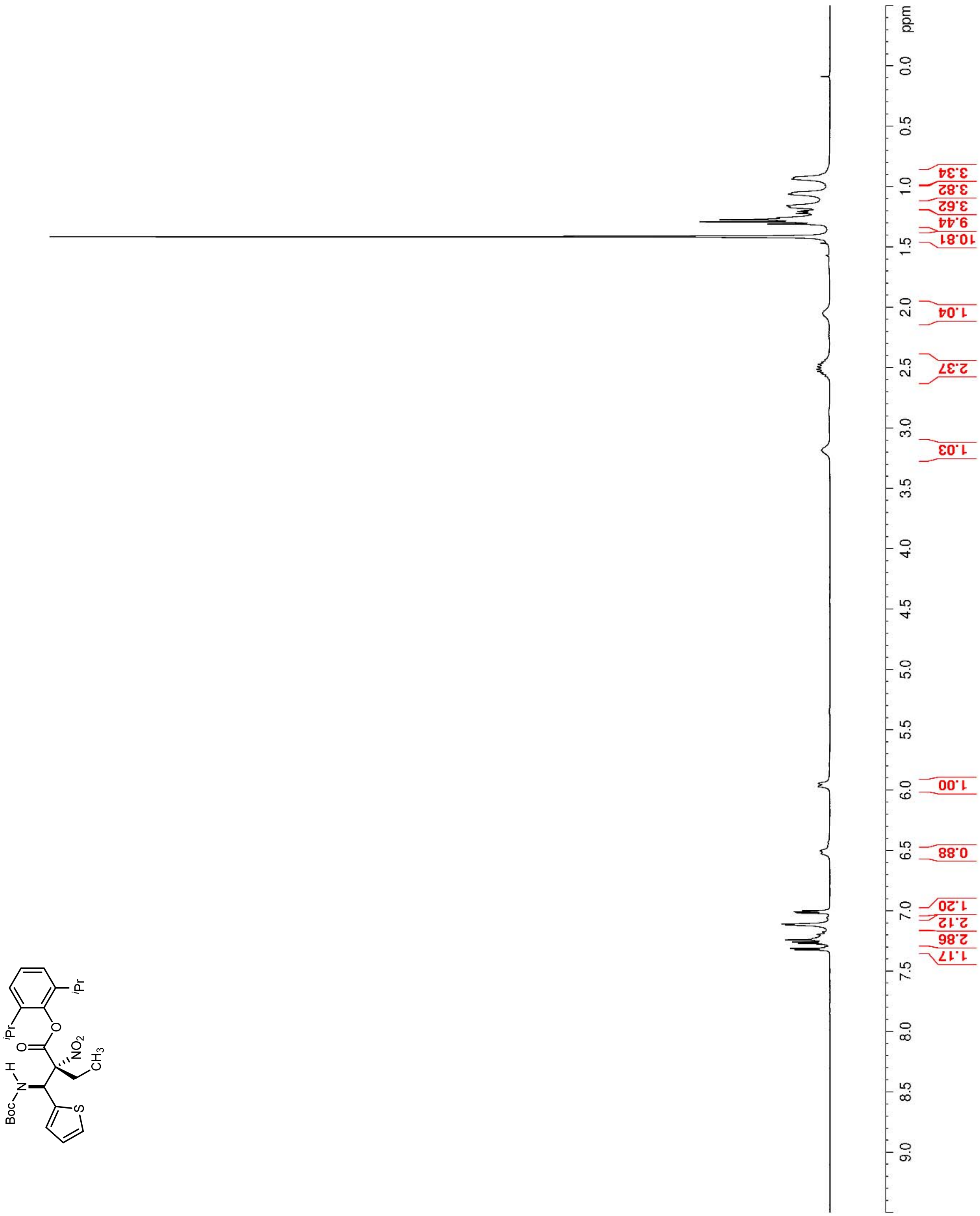

Figure 38. ^{13}C NMR (100 MHz, $CDCl_3$) of **13p**

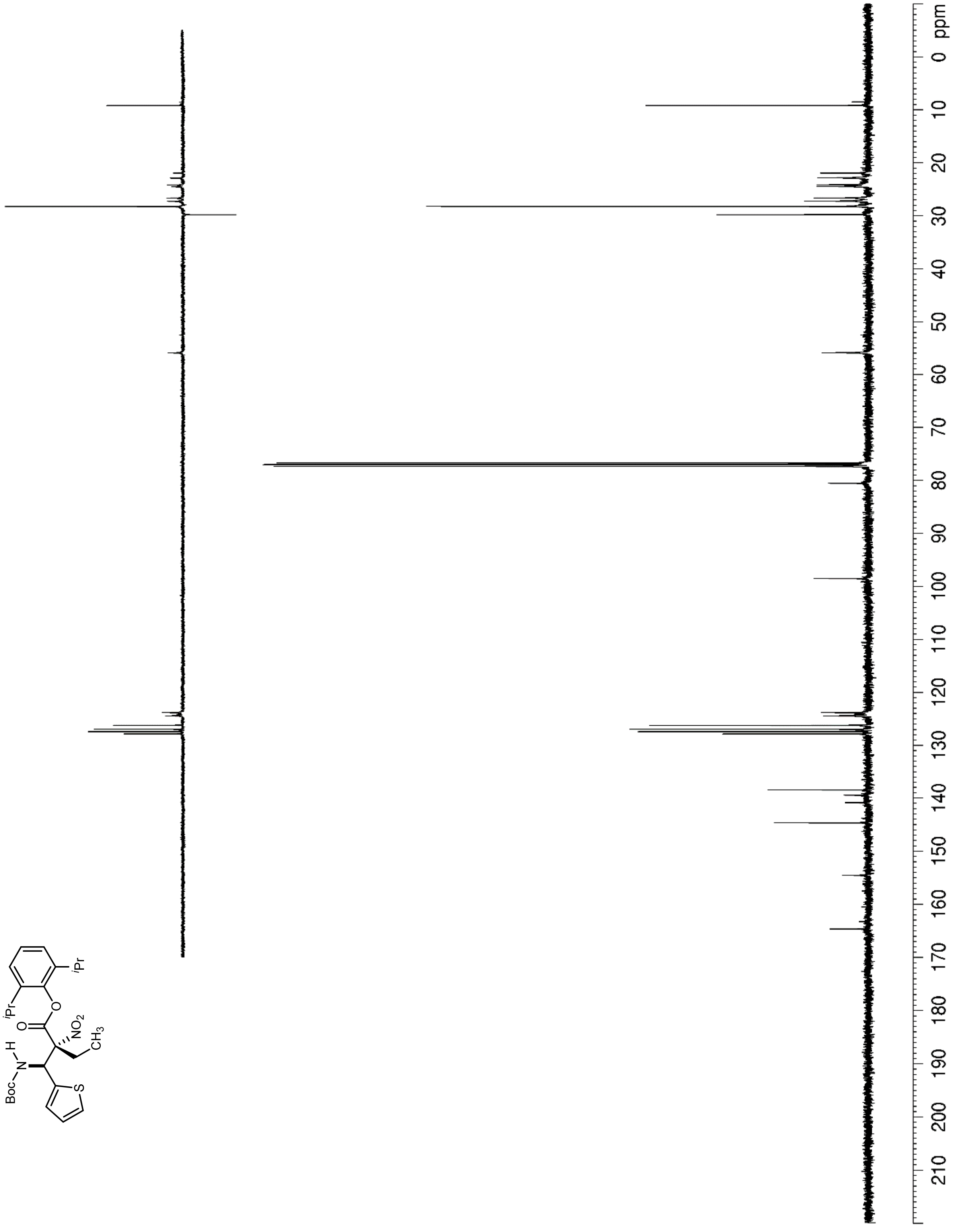

Figure 39. ^{1}H NMR (400 MHz, $CDCl_3$) of **13r**

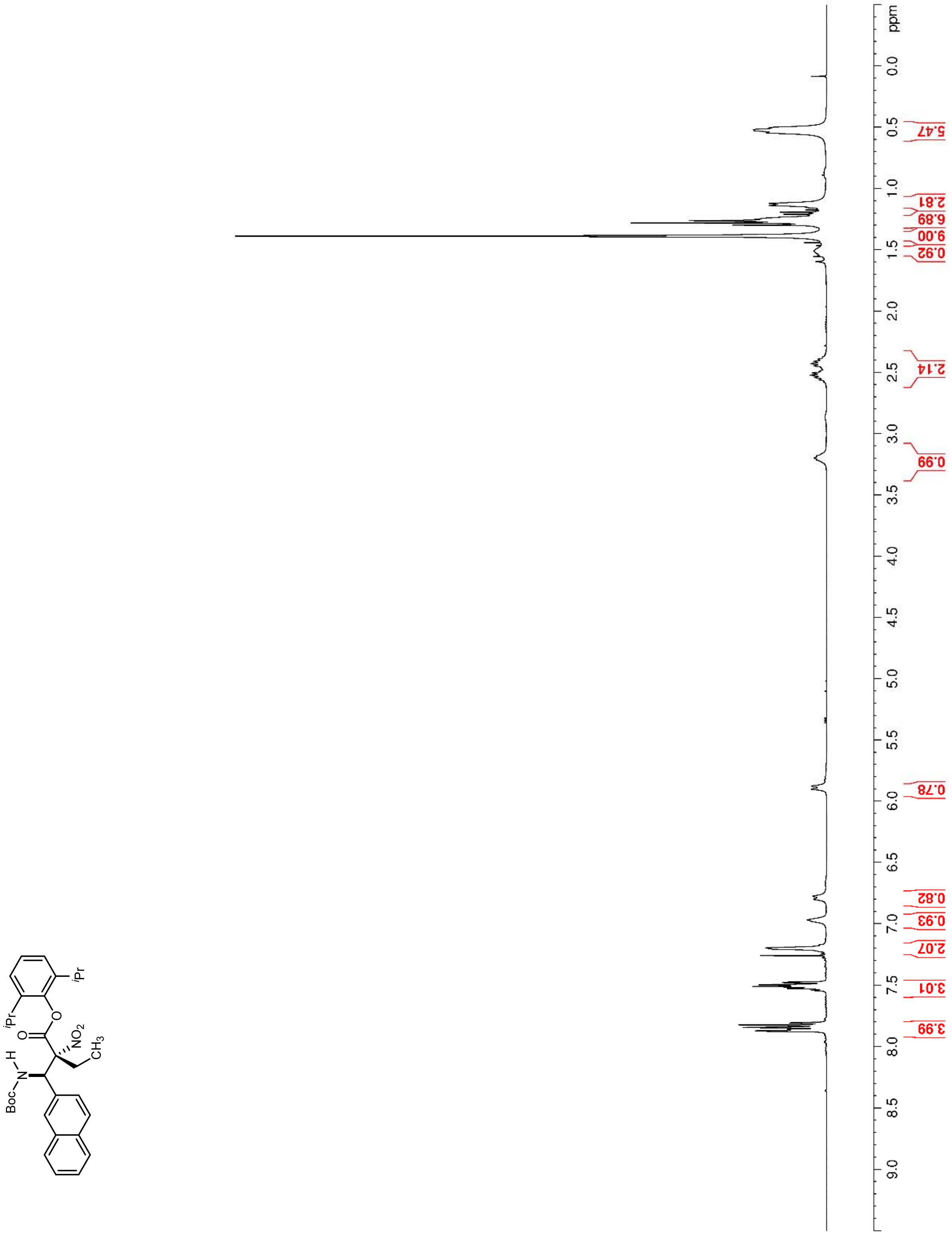

Figure 40. ^{13}C NMR (100 MHz, $CDCl_3$) of **13r**

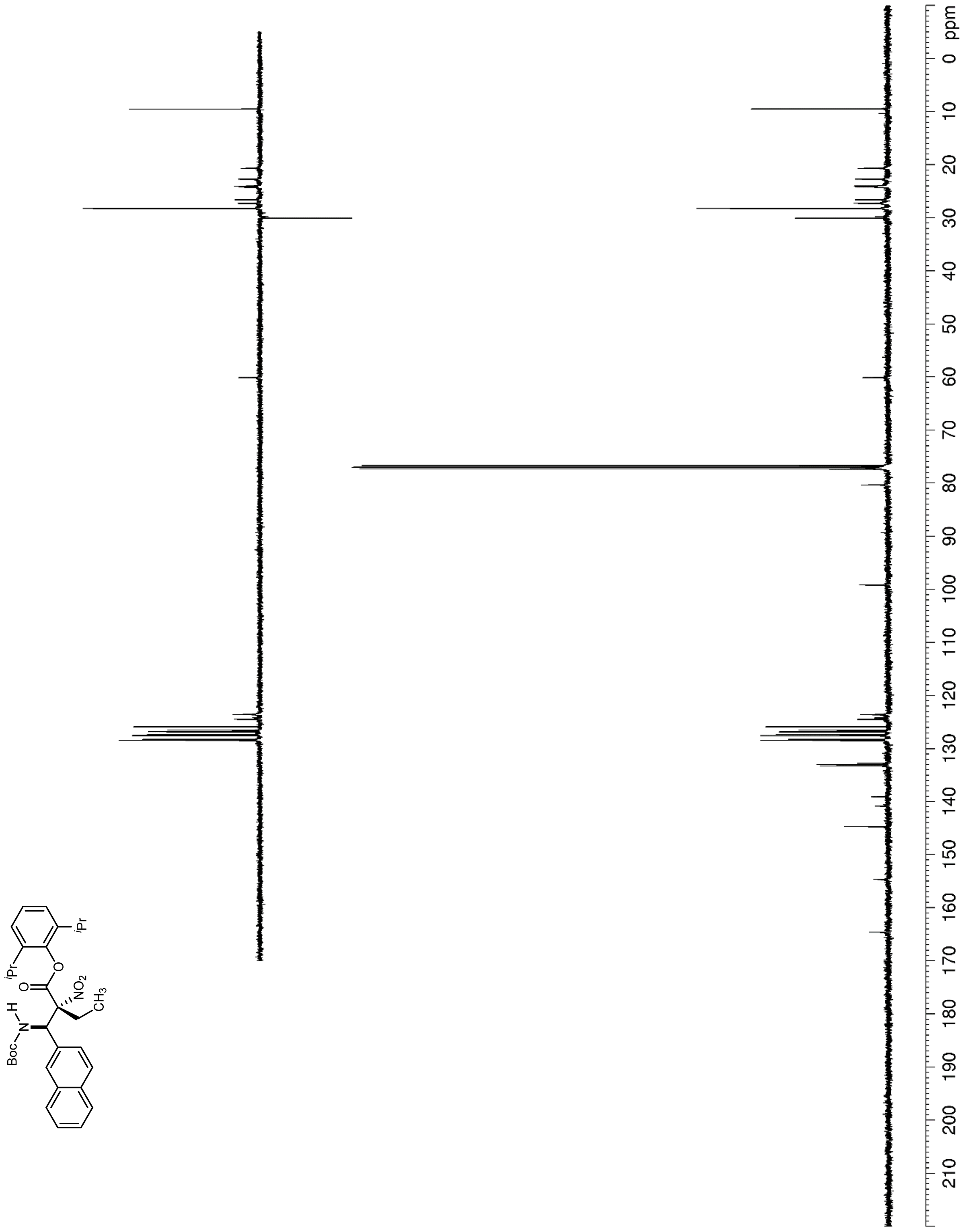

Figure 41. ^{1}H NMR (500 MHz, $CDCl_3$) of **13s**

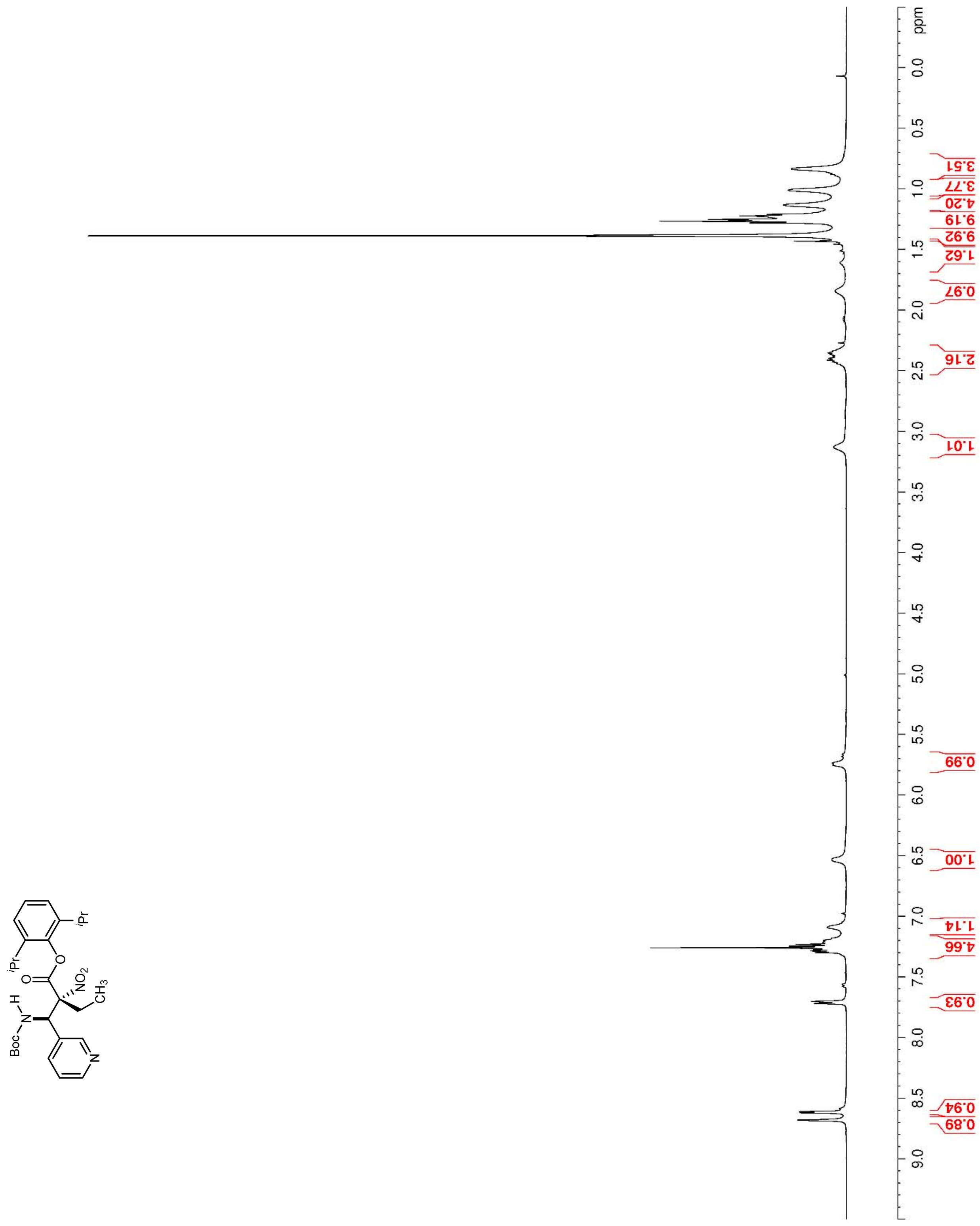

Figure 42. ^{13}C NMR (125 MHz, $CDCl_3$) of **13s**

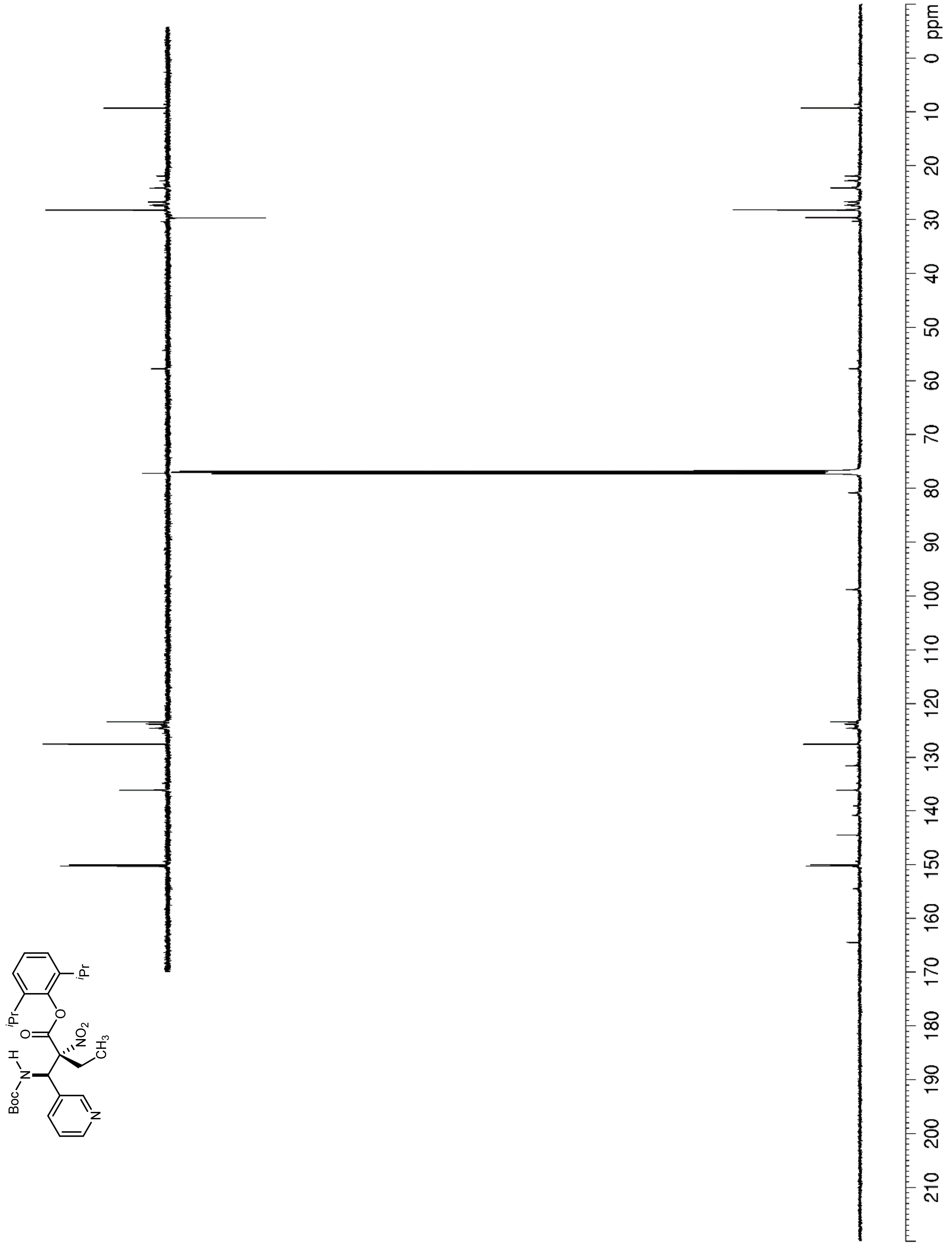

Figure 43. ^{1}H NMR (500 MHz, $CDCl_3$) of **13t**

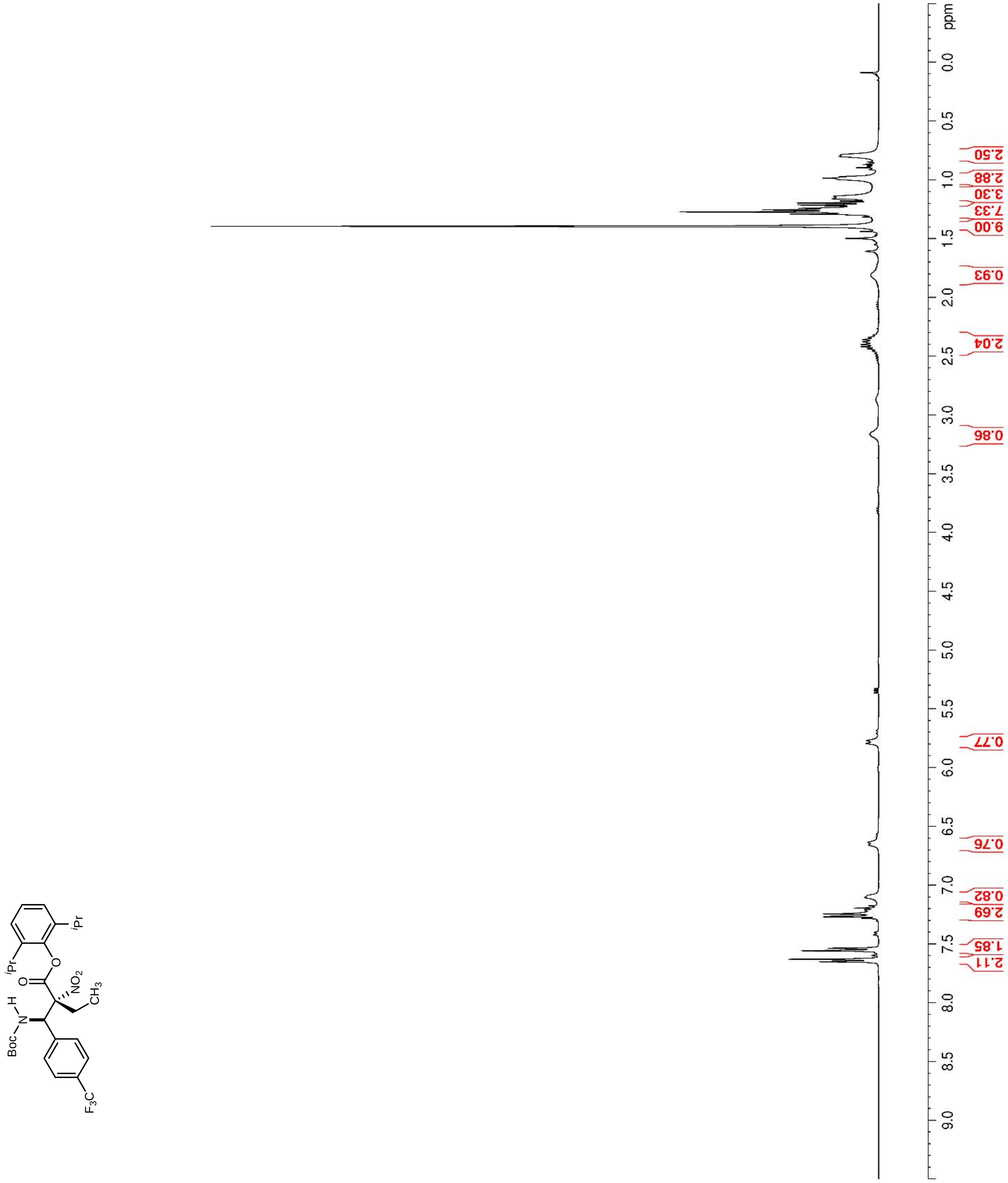

Figure 44. ^{13}C NMR (125 MHz, $CDCl_3$) of **13t**

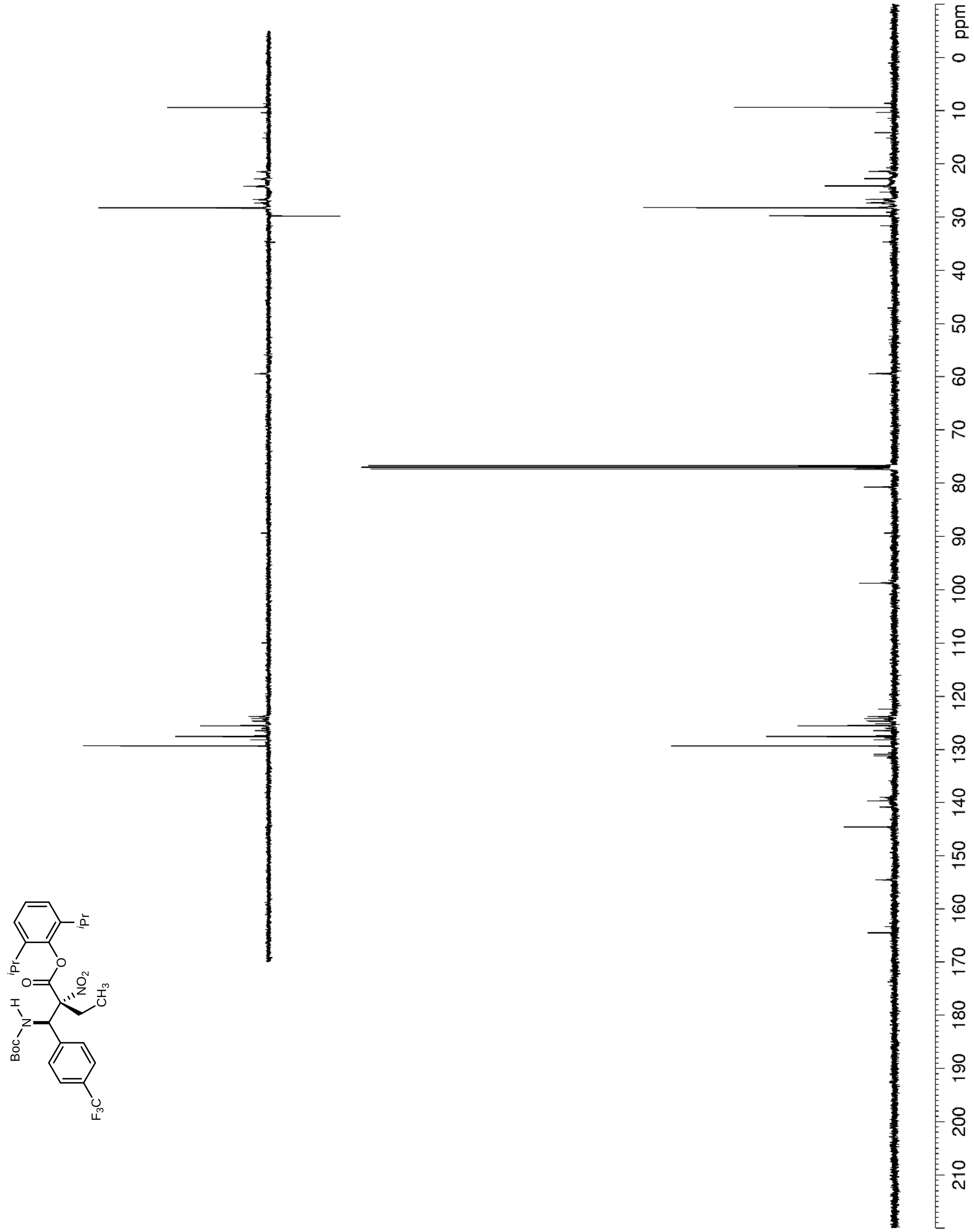

Figure 45. ^1H NMR (400 MHz, $CDCl_3$) of **13u**

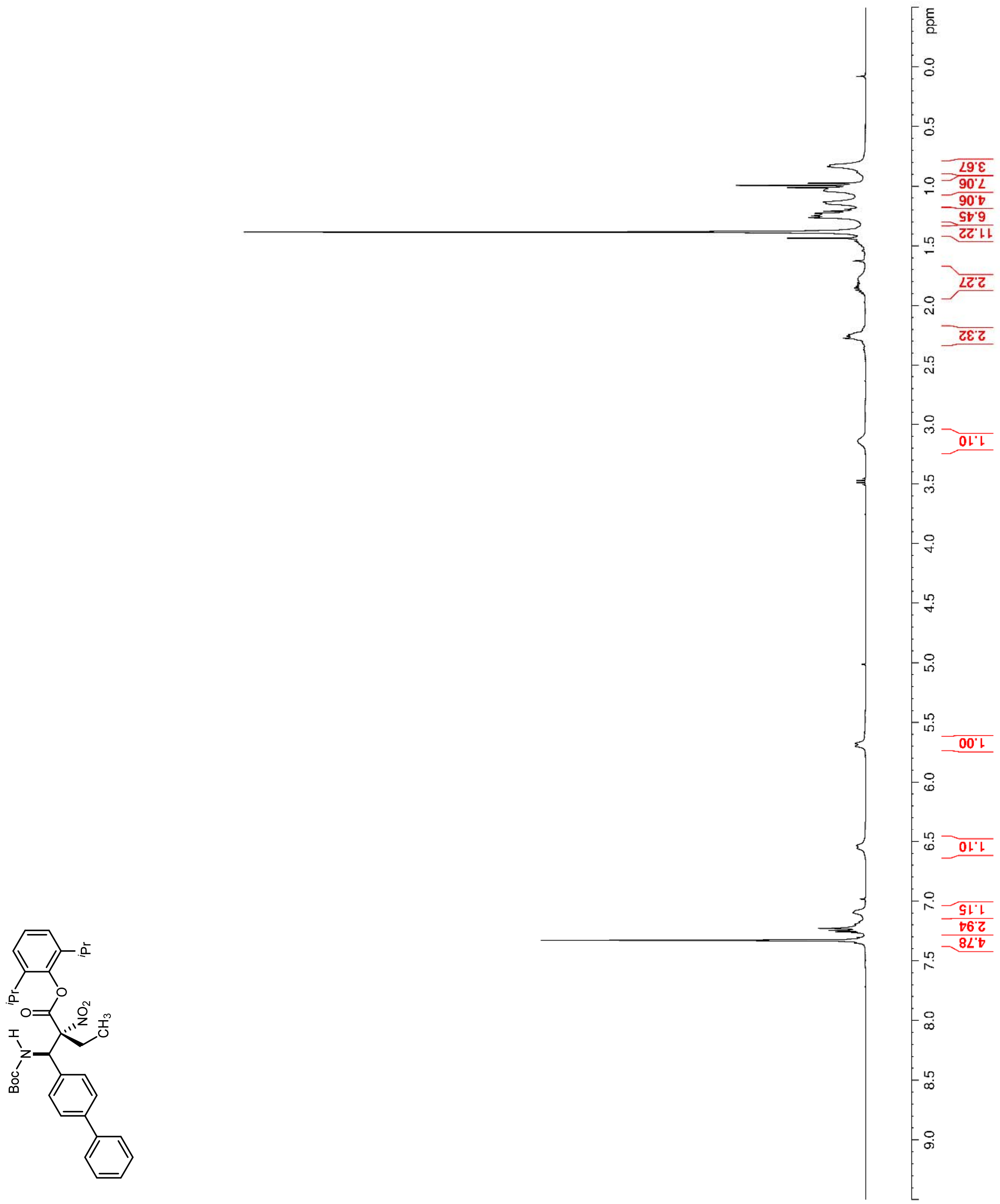

Figure 46. ^{13}C NMR (100 MHz, $CDCl_3$) of **13u**

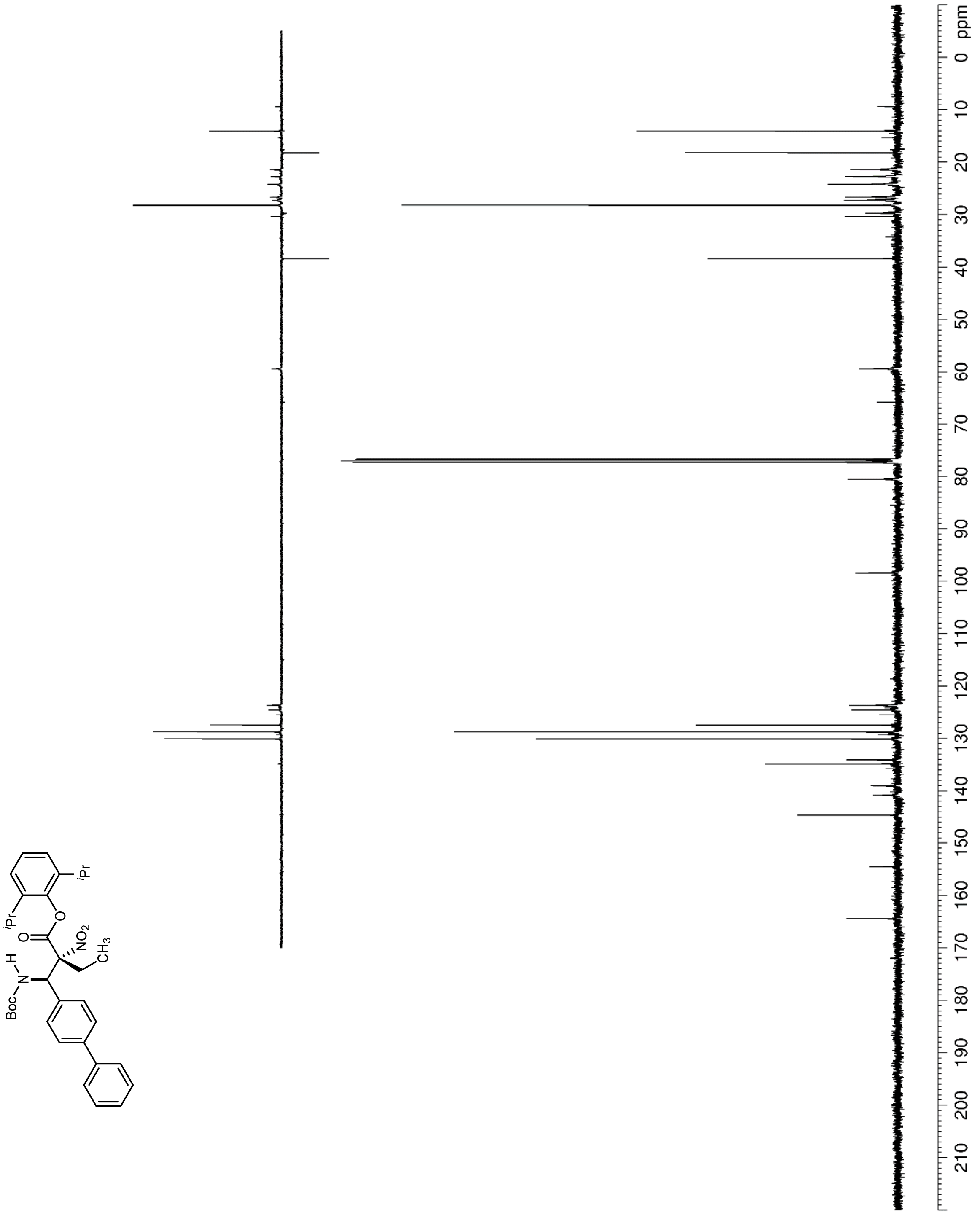

Figure 47. ^{1}H NMR (400 MHz, $CDCl_3$) of **14**

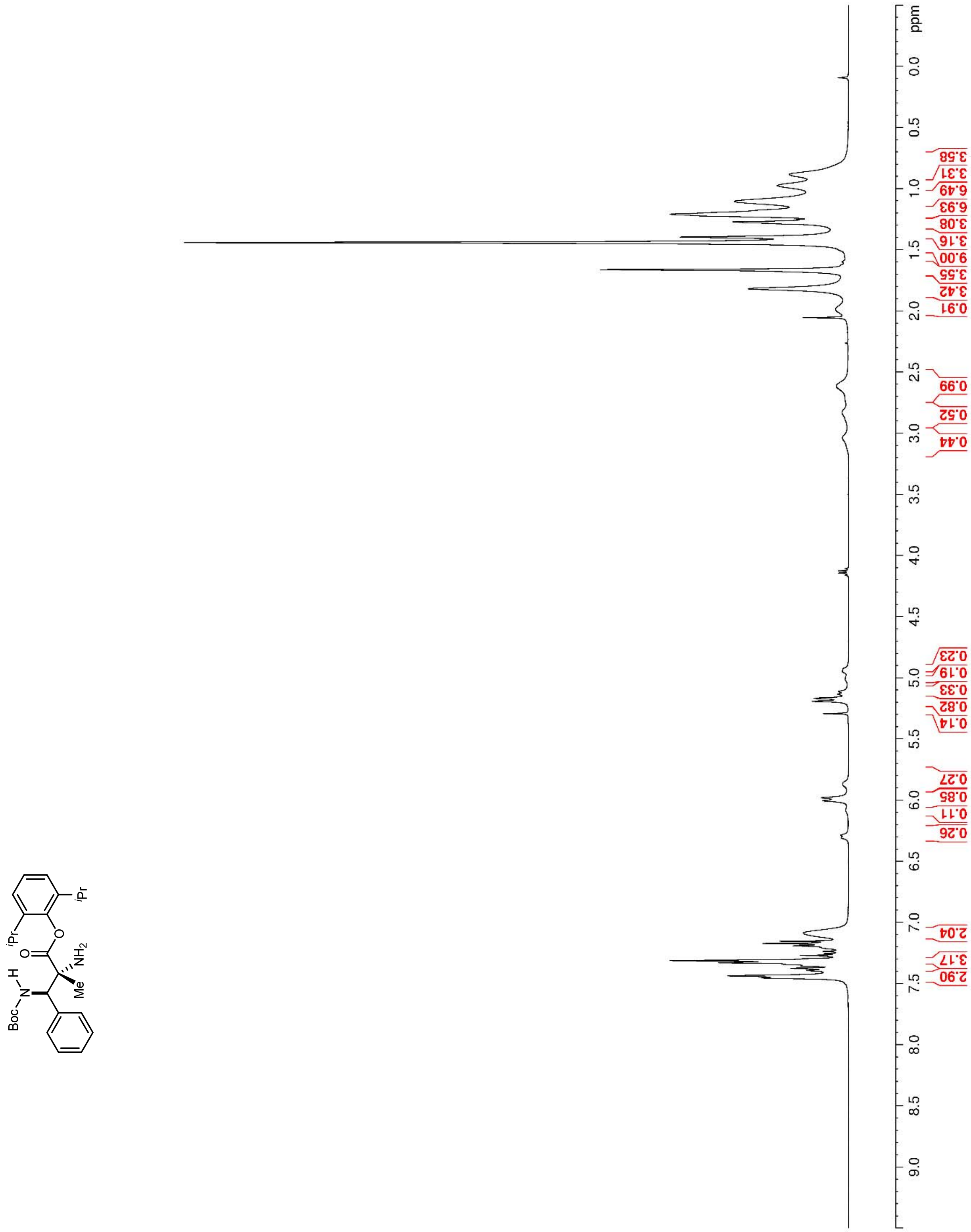

Figure 48. ^{13}C NMR (100 MHz, $CDCl_3$) of **14**

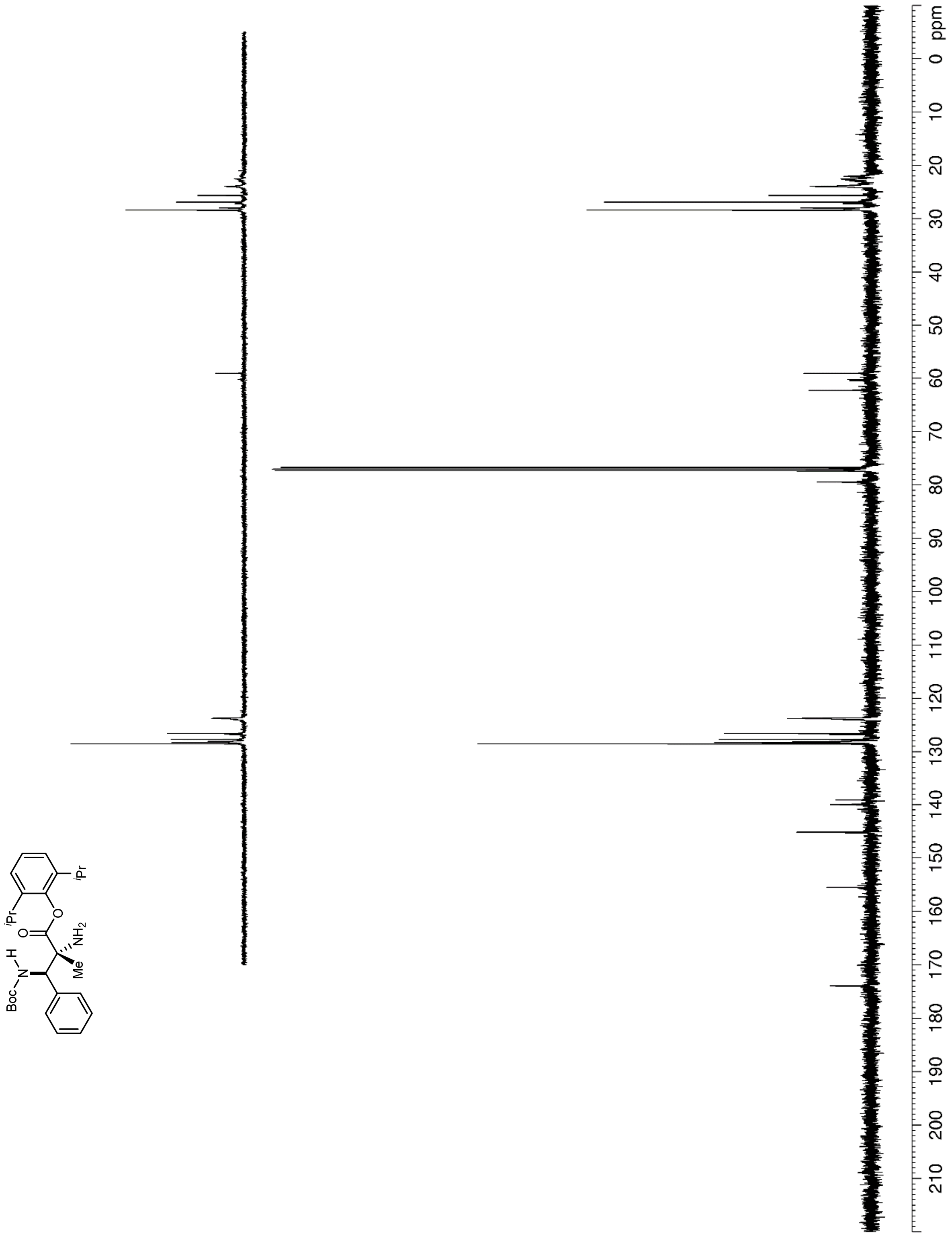

Figure 49. ^{1}H NMR (500 MHz, DMSO-d_6) of **SI-1**

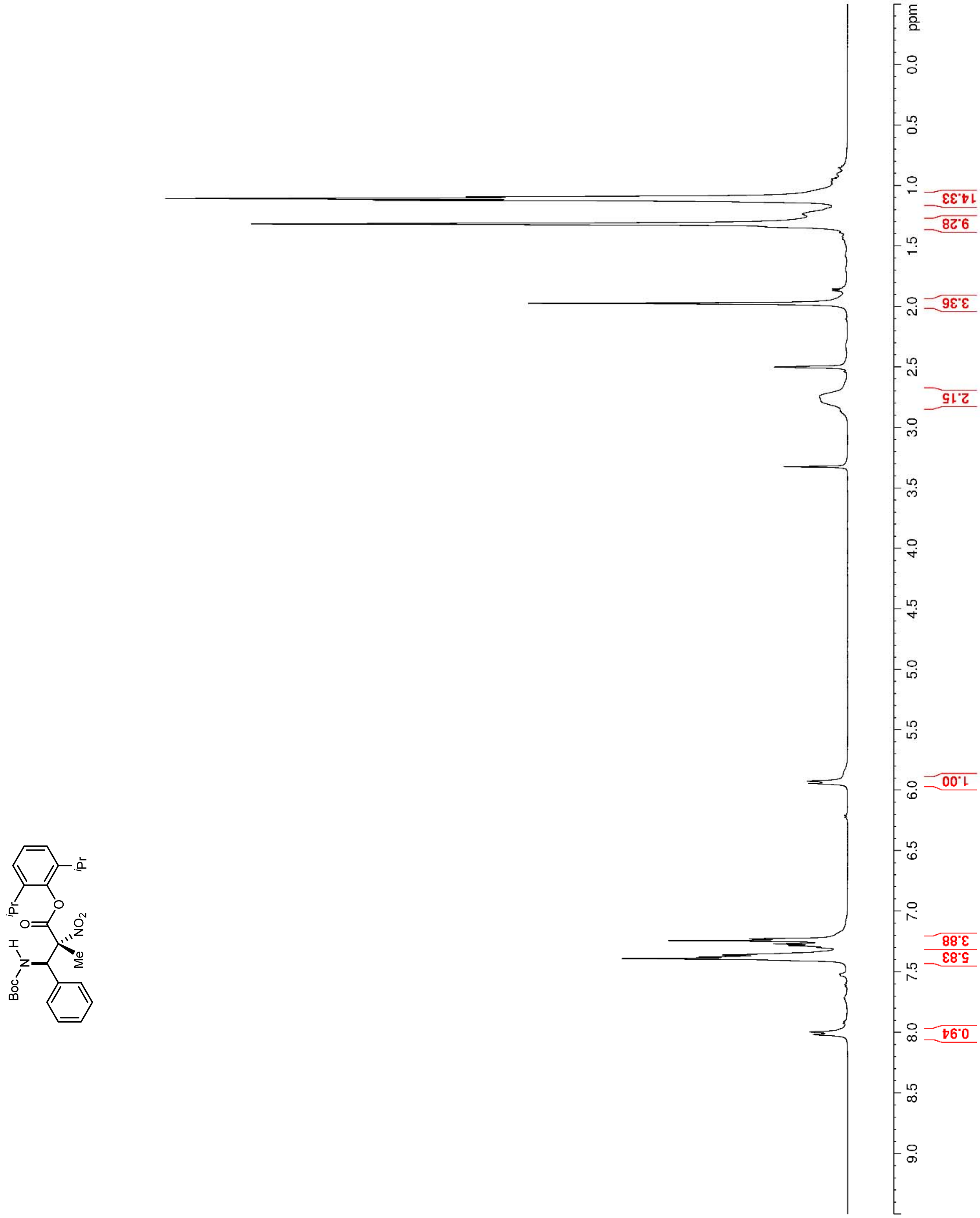

Figure 50. ^{13}C NMR (125 MHz, DMSO-d_6) of **SI-1**

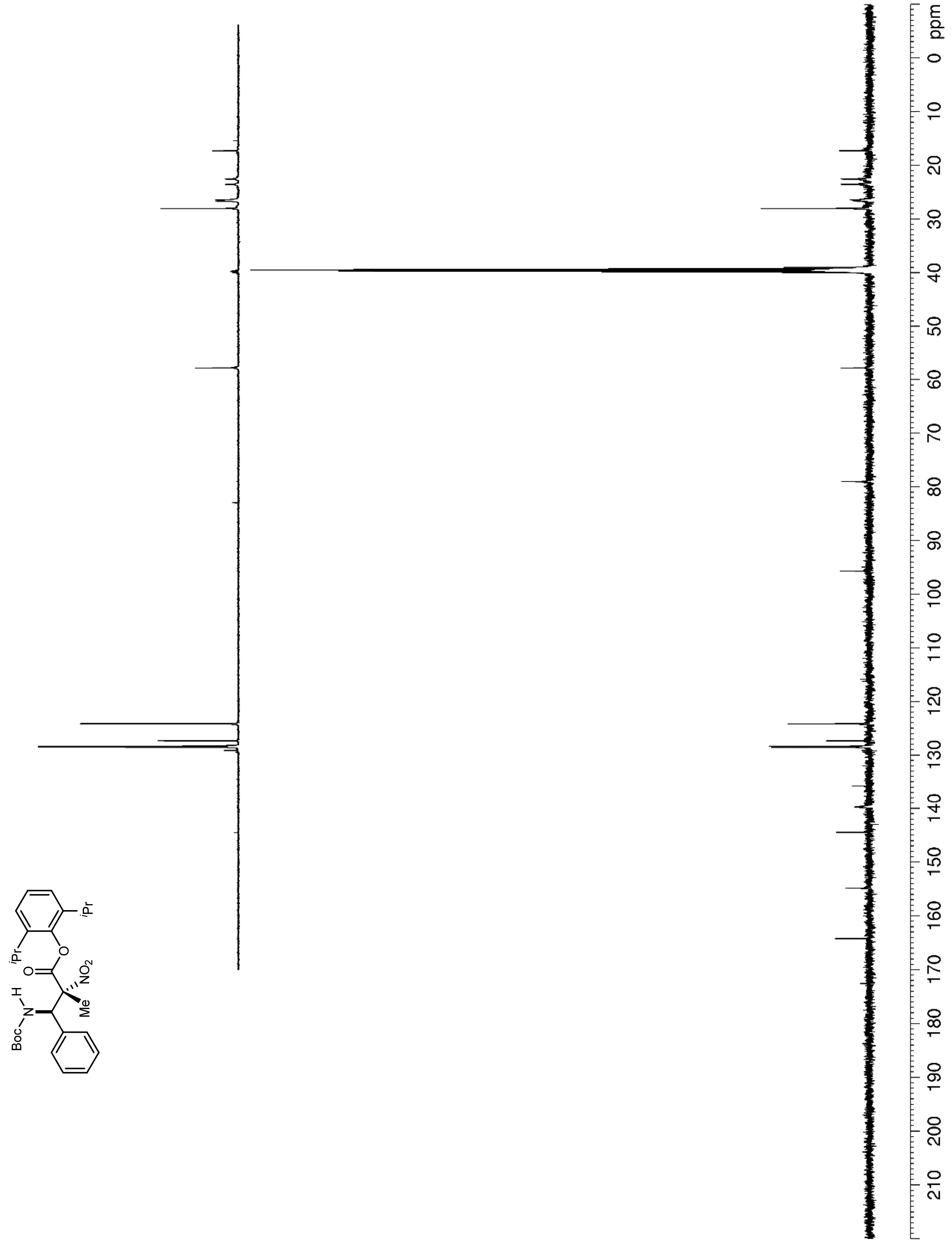

Figure 51. HPLC trace of **13a**

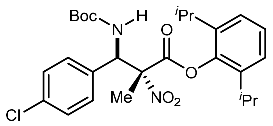

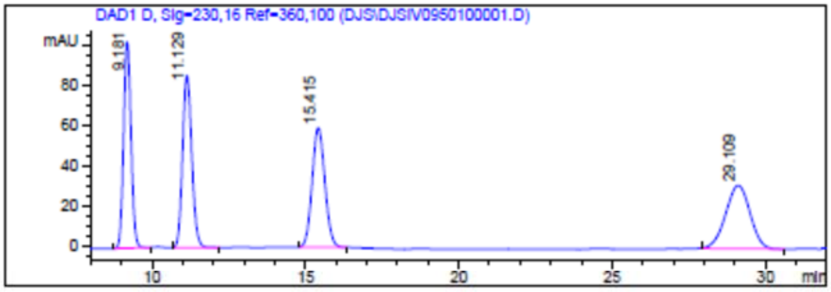

Signal 1: DAD1 D, Sig=230,16 Ref=360,100

Peak #	RT [min]	Width [min]	Area	Area %
1	9.181	0.281	1732.558	24.76
2	11.129	0.350	1797.143	25.68
3	15.415	0.483	1719.821	24.58
4	29.109	0.924	1748.465	24.99

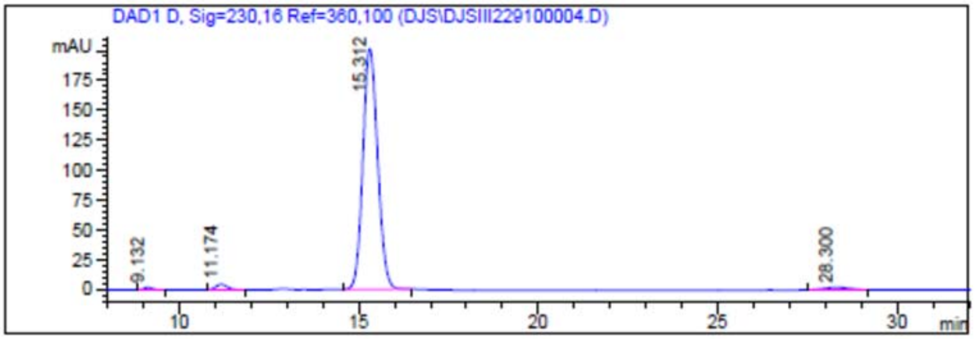

Signal 1: DAD1 D, Sig=230,16 Ref=360,100

Peak #	RT [min]	Width [min]	Area	Area %
1	9.132	0.287	35.665	0.59
2	11.174	0.384	101.297	1.69
3	15.312	0.476	5748.913	95.73
4	28.300	0.881	119.543	1.99

Figure 52. HPLC trace of **13b**

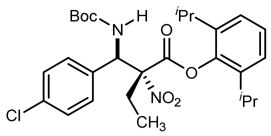

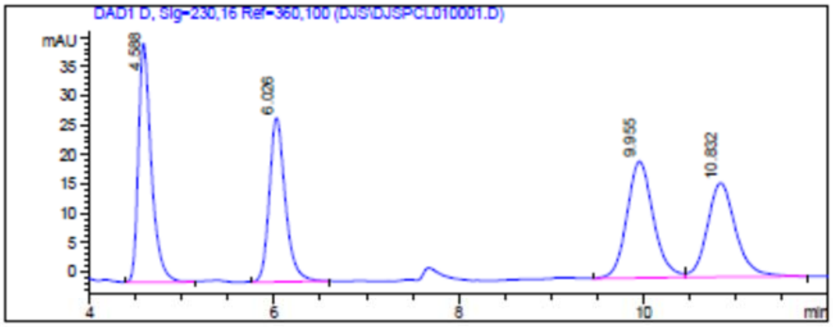

Signal 1: DAD1 D, Sig=230,16 Ref=360,100

Peak #	RT [min]	Width [min]	Area	Area %
1	4.588	0.162	395.129	26.83
2	6.026	0.201	337.076	22.89
3	9.955	0.332	397.256	26.98
4	10.832	0.355	343.018	23.30

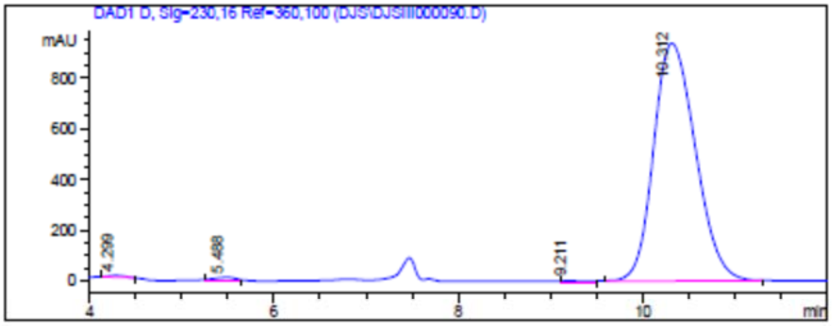

Signal 1: DAD1 D, Sig=230,16 Ref=360,100

Peak #	RT [min]	Width [min]	Area	Area %
1	4.299	0.204	81.523	0.27
2	5.488	0.245	158.381	0.53
3	9.211	0.358	54.916	0.18
4	10.312	0.526	29516.441	99.01

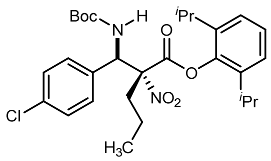

Figure 53. HPLC trace of **13c**

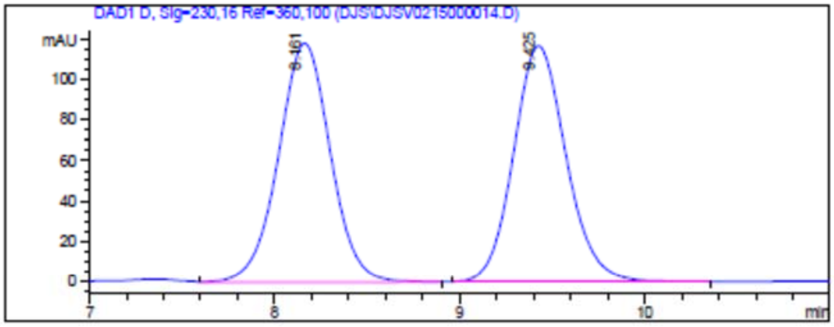

Signal 1: DAD1 D, Sig=230,16 Ref=360,100

Peak #	RT [min]	Width [min]	Area	Area %
1	8.161	0.327	2319.935	49.97
2	9.425	0.331	2322.924	50.03

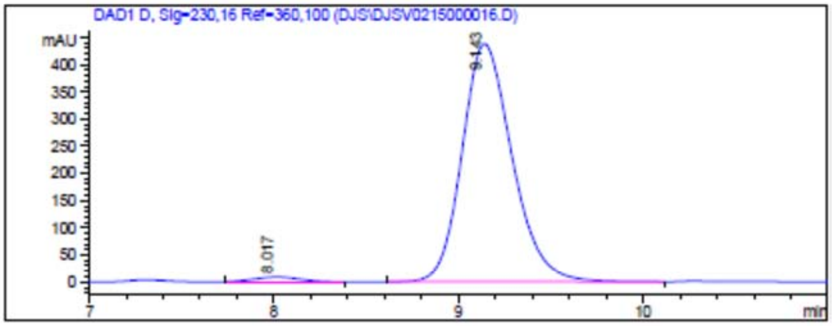

Signal 1: DAD1 D, Sig=230,16 Ref=360,100

Peak #	RT [min]	Width [min]	Area	Area %
1	8.017	0.276	138.022	1.64
2	9.143	0.315	8275.297	98.36

Figure 54. HPLC trace of **13d**

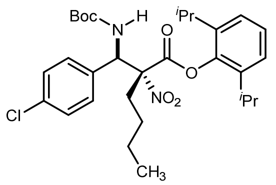

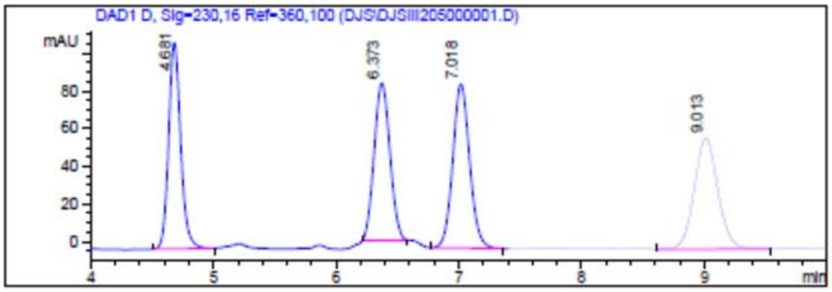

Signal 1: DAD1 D, Sig=230,16 Ref=360,100

Peak #	RT [min]	Width [min]	Area	Area %
1	4.681	0.114	748.867	23.96
2	6.373	0.149	750.449	24.01
3	7.018	0.159	834.238	26.69
4	9.013	0.223	792.462	25.35

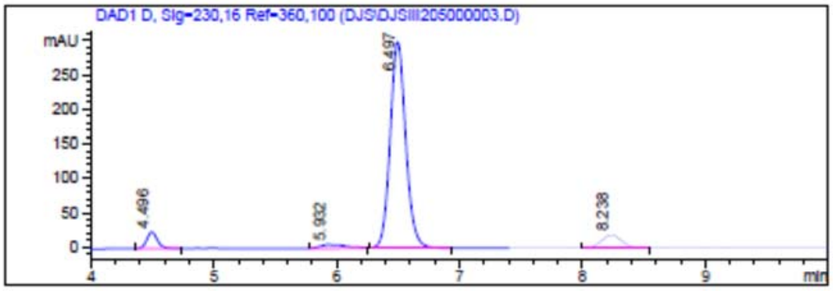

Signal 1: DAD1 D, Sig=230,16 Ref=360,100

Peak #	RT [min]	Width [min]	Area	Area %
1	4.496	0.098	141.020	4.57
2	5.932	0.244	79.387	2.57
3	6.497	0.147	2635.708	85.49
4	8.238	0.195	227.084	7.37

Figure 55. HPLC trace of **13e**

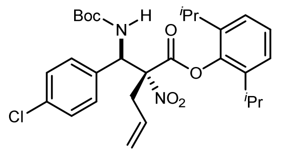

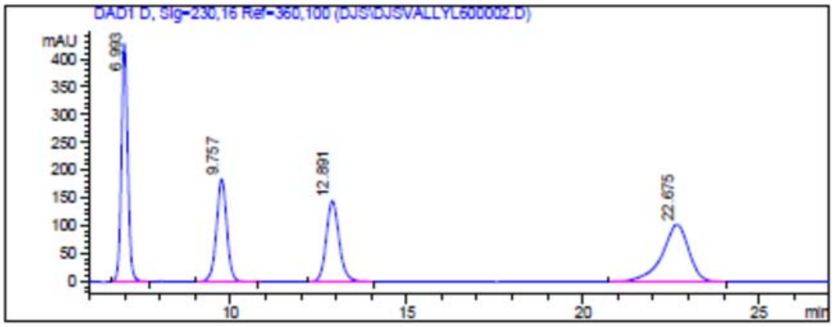

Signal 1: DAD1 D, Sig=230,16 Ref=360,100

Peak #	RT [min]	Width [min]	Area	Area %
1	6.993	0.209	5376.496	29.02
2	9.757	0.343	3800.438	20.51
3	12.891	0.434	3809.733	20.56
4	22.675	0.896	5542.191	29.91

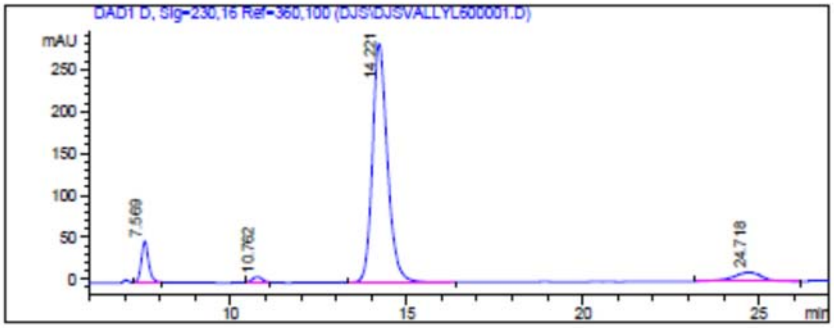

Signal 1: DAD1 D, Sig=230,16 Ref=360,100

Peak #	RT [min]	Width [min]	Area	Area %
1	7.569	0.239	702.016	7.12
2	10.762	0.332	130.307	1.32
3	14.221	0.494	8446.417	85.62
4	24.718	0.907	586.605	5.95

Figure 56. HPLC trace of **13f**[1]

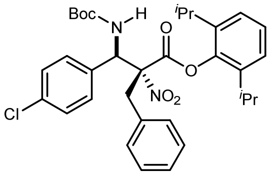

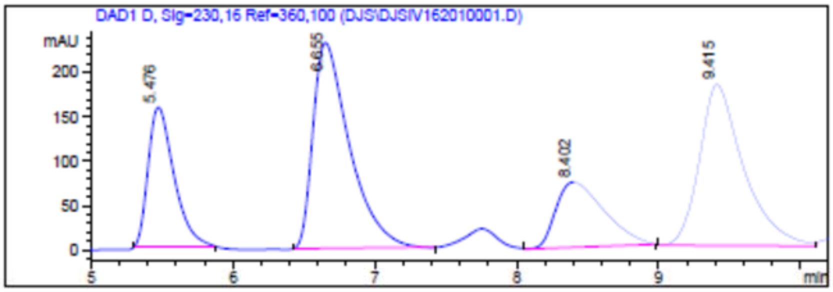

Signal 1: DAD1 D, Sig=230,16 Ref=360,100

Peak #	RT [min]	Width [min]	Area	Area %
1	5.476	0.209	1962.621	16.65
2	6.655	0.299	4154.844	35.24
3	8.402	0.391	1727.450	14.65
4	9.415	0.362	3944.966	33.46

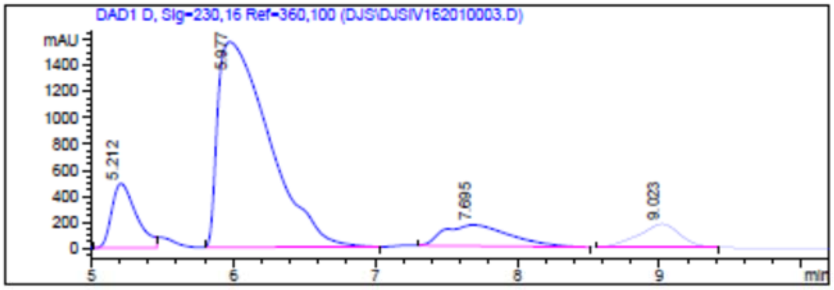

Signal 1: DAD1 D, Sig=230,16 Ref=360,100

Peak #	RT [min]	Width [min]	Area	Area %
1	5.212	0.201	5970.524	10.97
2	5.977	0.420	39633.594	72.81
3	7.695	0.536	5205.801	9.56
4	9.023	0.338	3622.799	6.66

[1] Product was unable to be separated from starting material impurity by column chromatography, however, all 4 peaks of the product are unaffected and visible in the HPLC chromatogram, allowing for determination of selectivity from the reaction (the *anti* peaks are peaks 2 and 4 in the spectrum).

Figure 57. HPLC trace of **13g**

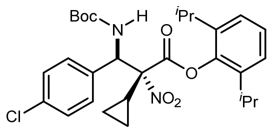

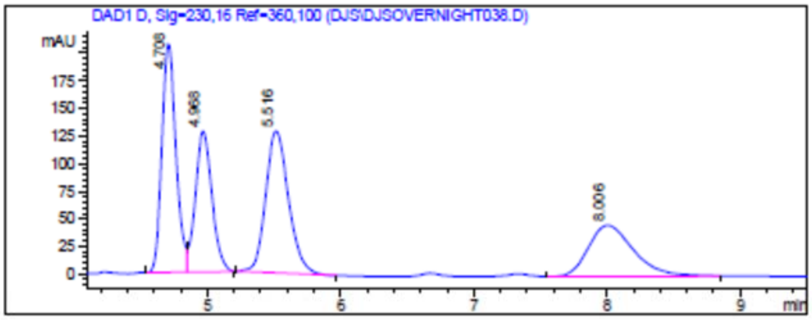

Signal 1: DAD1 D, Sig=230,16 Ref=360,100

Peak #	RT [min]	Width [min]	Area	Area %
1	4.708	0.123	1521.462	29.05
2	4.968	0.144	1101.792	21.04
3	5.516	0.198	1525.785	29.13
4	8.006	0.392	1088.827	20.79

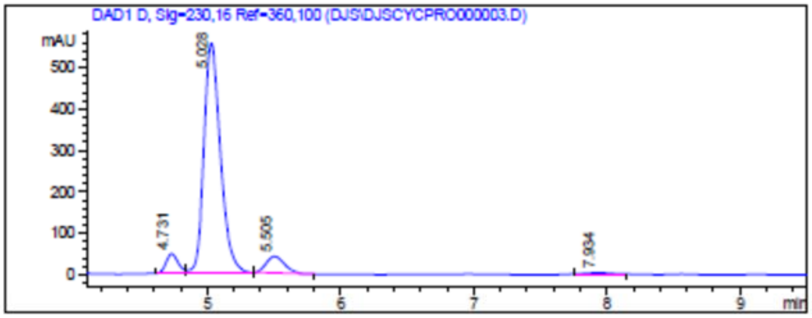

Signal 1: DAD1 D, Sig=230,16 Ref=360,100

Peak #	RT [min]	Width [min]	Area	Area %
1	4.731	0.105	301.070	5.43
2	5.028	0.144	4815.134	86.83
3	5.505	0.162	388.966	7.01
4	7.934	0.226	40.401	0.73

Figure 58. HPLC trace of **13h**

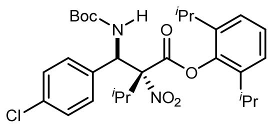

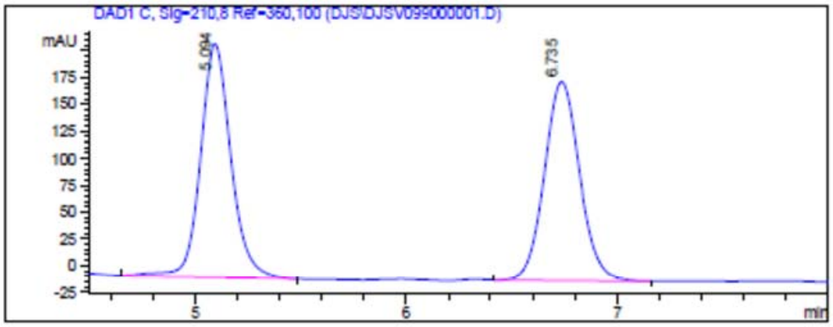

Signal 1: DAD1 C, Sig=210,8 Ref=360,100

Peak #	RT [min]	Width [min]	Area	Area %
1	5.094	0.169	2200.285	50.56
2	6.735	0.194	2151.210	49.44

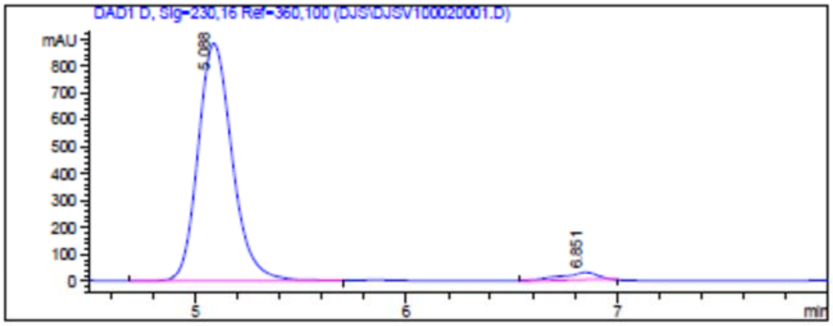

Signal 1: DAD1 D, Sig=230,16 Ref=360,100

Peak #	RT [min]	Width [min]	Area	Area %
1	5.088	0.190	10103.221	96.73
2	6.851	0.205	341.890	3.27

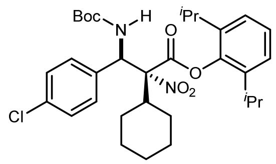

Figure 59. HPLC trace of **13i**

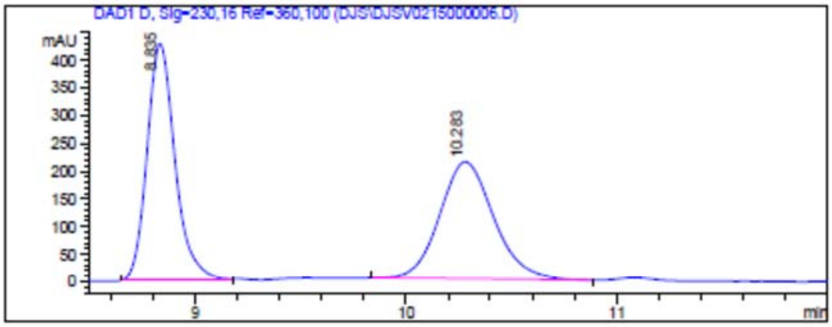

Signal 1: DAD1 D, Sig=230,16 Ref=360,100

Peak #	RT [min]	Width [min]	Area	Area %
1	8.835	0.152	3883.956	50.58
2	10.283	0.298	3795.124	49.42

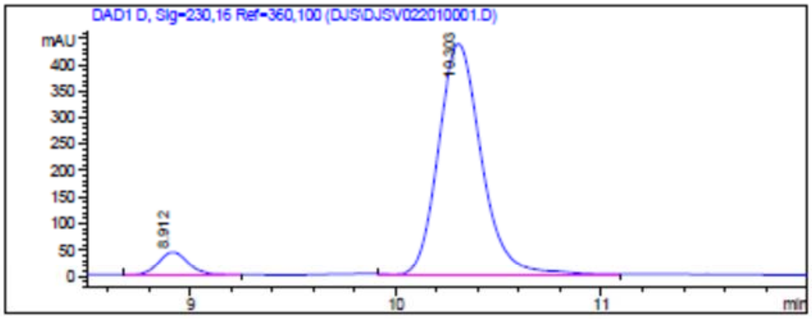

Signal 1: DAD1 D, Sig=230,16 Ref=360,100

Peak #	RT [min]	Width [min]	Area	Area %
1	8.912	0.168	444.695	6.54
2	10.303	0.242	6352.126	93.46

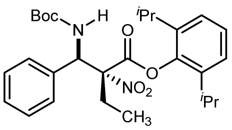

Figure 60. HPLC trace of **13j**

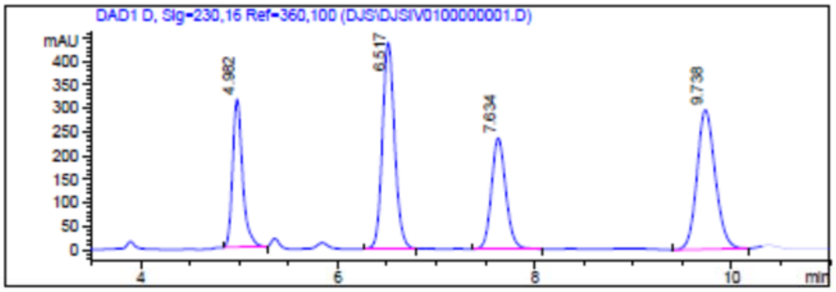

Signal 1: DAD1 D, Sig=230,16 Ref=360,100

Peak #	RT [min]	Width [min]	Area	Area %
1	4.982	0.117	2200.071	18.03
2	6.517	0.144	3777.581	30.95
3	7.634	0.169	2401.456	19.68
4	9.738	0.215	3825.402	31.34

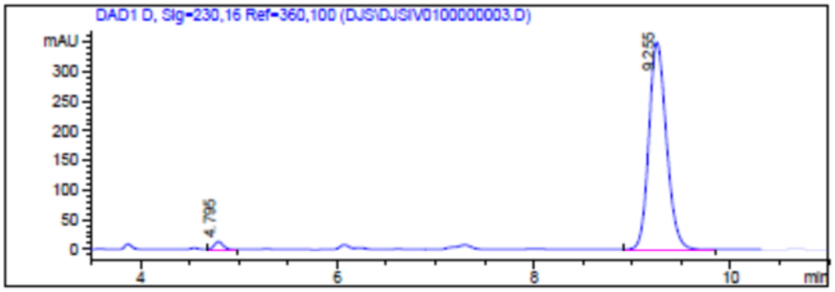

Signal 1: DAD1 D, Sig=230,16 Ref=360,100

Peak #	RT [min]	Width [min]	Area	Area %
1	4.795	0.105	86.437	1.94
2	9.255	0.207	4359.929	98.06

Figure 61. HPLC trace of **13k**

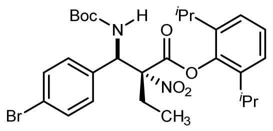

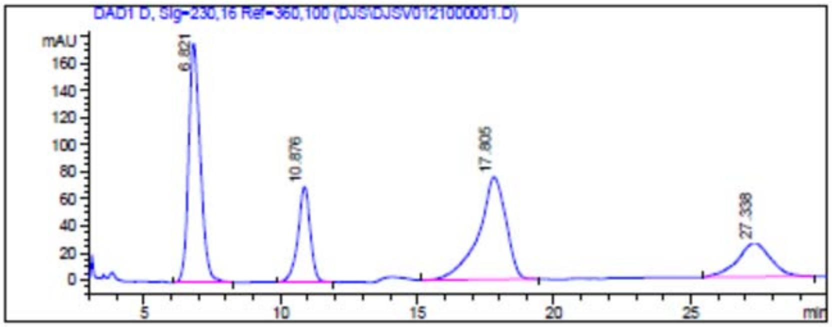

Signal 1: DAD1 D, Sig=230,16 Ref=360,100

Peak #	RT [min]	Width [min]	Area	Area %
1	6.821	0.496	5261.827	35.25
2	10.876	0.525	2226.999	14.92
3	17.805	1.169	5321.908	35.65
4	27.338	1.407	2116.072	14.18

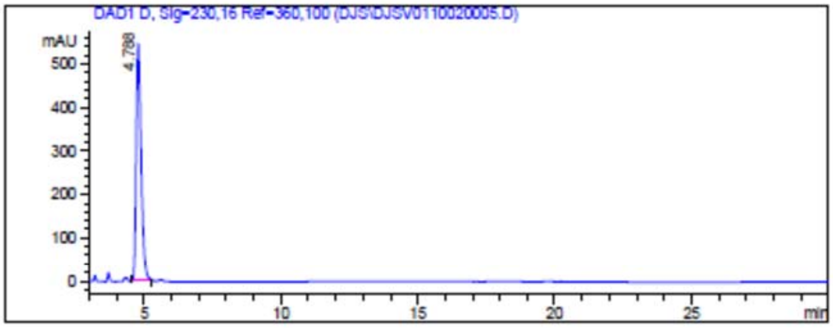

Signal 1: DAD1 D, Sig=230,16 Ref=360,100

Peak #	RT [min]	Width [min]	Area	Area %
1	4.788	0.213	6961.615	100.00

Figure 62. HPLC trace of **13l**

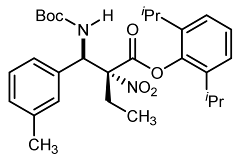

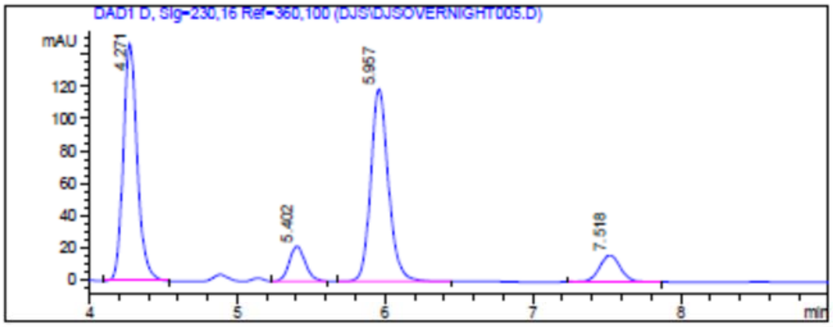

Signal 1: DAD1 D, Sig=230,16 Ref=360,100

Peak #	RT [min]	Width [min]	Area	Area %
1	4.271	0.110	968.415	42.34
2	5.402	0.125	163.946	7.17
3	5.957	0.139	993.731	43.45
4	7.518	0.161	161.119	7.04

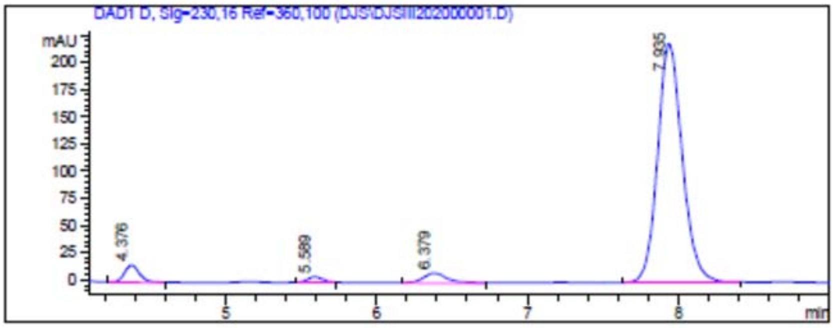

Signal 1: DAD1 D, Sig=230,16 Ref=360,100

Peak #	RT [min]	Width [min]	Area	Area %
1	4.376	0.117	112.484	4.10
2	5.589	0.113	33.327	1.21
3	6.379	0.187	102.333	3.73
4	7.935	0.189	2495.709	90.96

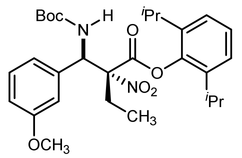

Figure 63. HPLC trace of **13m**

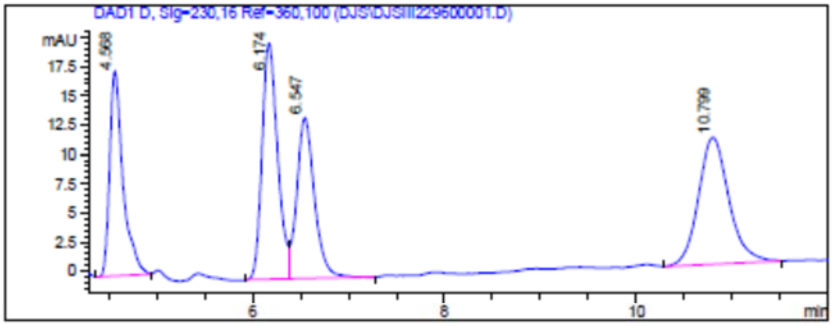

Signal 1: DAD1 D, Sig=230,16 Ref=360,100

Peak #	RT [min]	Width [min]	Area	Area %
1	4.568	0.161	169.195	20.84
2	6.174	0.188	228.191	28.11
3	6.547	0.213	175.896	21.67
4	10.799	0.367	238.463	29.38

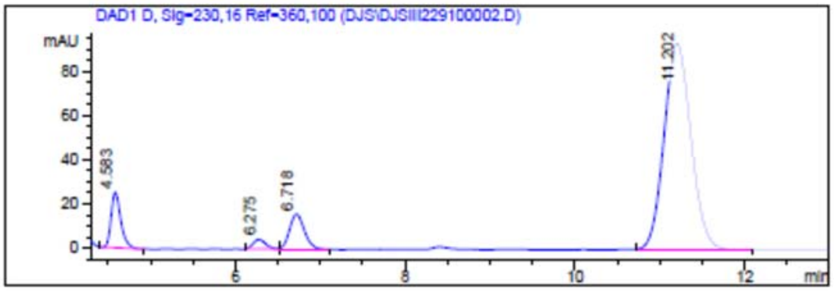

Signal 1: DAD1 D, Sig=230,16 Ref=360,100

Peak #	RT [min]	Width [min]	Area	Area %
1	4.583	0.132	198.499	8.02
2	6.275	0.172	42.868	1.73
3	6.718	0.201	190.837	7.71
4	11.202	0.365	2043.087	82.54

Figure 64. HPLC trace of **13o**

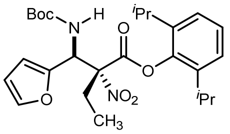

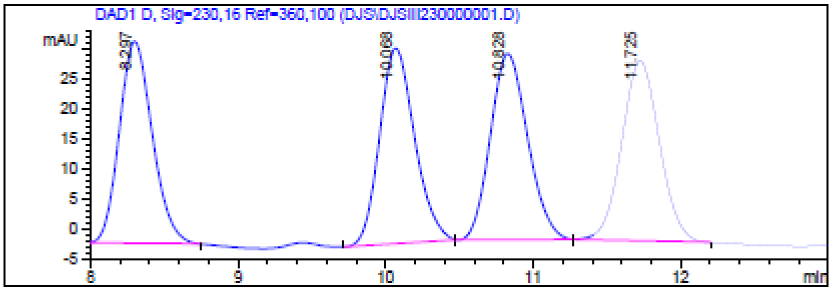

Signal 1: DAD1 D, Sig=230,16 Ref=360,100

Peak #	RT [min]	Width [min]	Area	Area %
1	8.297	0.259	524.881	24.45
2	10.068	0.276	541.293	25.21
3	10.828	0.292	546.372	25.45
4	11.725	0.296	534.589	24.90

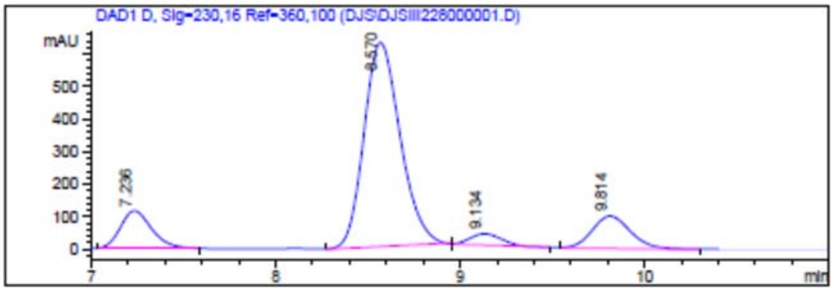

Signal 1: DAD1 D, Sig=230,16 Ref=360,100

Peak #	RT [min]	Width [min]	Area	Area %
1	7.236	0.184	1230.795	10.52
2	8.570	0.227	8585.271	73.42
3	9.134	0.194	405.434	3.47
4	9.814	0.244	1472.593	12.59

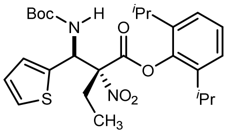

Figure 65. HPLC trace of **13p**

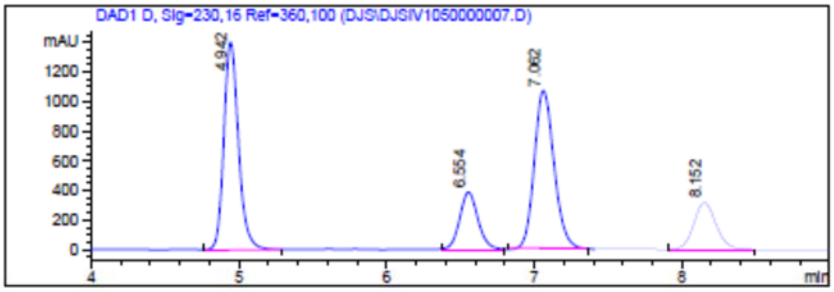

Signal 1: DAD1 D, Sig=230,16 Ref=360,100

Peak #	RT [min]	Width [min]	Area	Area %
1	4.942	0.115	9739.674	37.01
2	6.554	0.144	3361.386	12.77
3	7.062	0.154	9859.396	37.47
4	8.152	0.176	3355.291	12.75

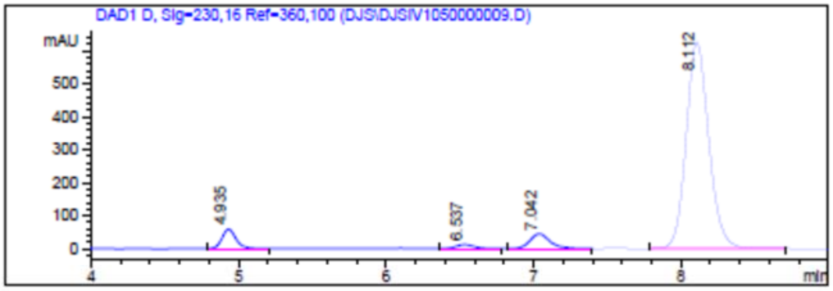

Signal 1: DAD1 D, Sig=230,16 Ref=360,100

Peak #	RT [min]	Width [min]	Area	Area %
1	4.935	0.109	388.537	5.12
2	6.537	0.141	102.983	1.36
3	7.042	0.158	422.848	5.57
4	8.112	0.178	6673.846	87.95

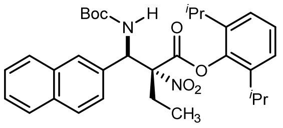

Figure 66. HPLC trace of **13r**

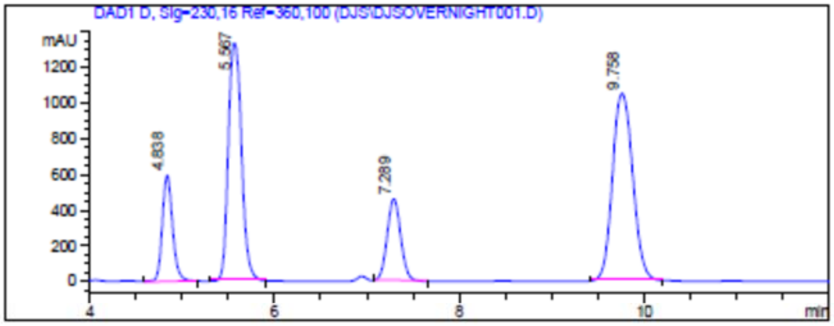

Signal 1: DAD1 D, Sig=230,16 Ref=360,100

Peak #	RT [min]	Width [min]	Area	Area %
1	4.838	0.129	4629.875	12.16
2	5.567	0.164	13068.275	34.32
3	7.289	0.167	4587.058	12.05
4	9.758	0.252	15791.271	41.47

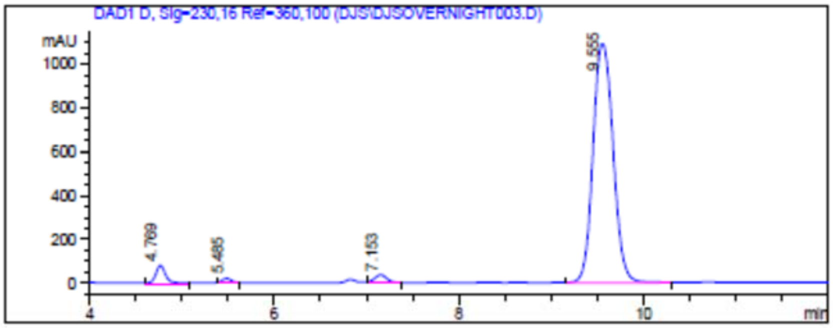

Signal 1: DAD1 D, Sig=230,16 Ref=360,100

Peak #	RT [min]	Width [min]	Area	Area %
1	4.769	0.131	665.205	3.74
2	5.485	0.111	127.301	0.72
3	7.153	0.156	330.658	1.86
4	9.555	0.254	16655.787	93.68

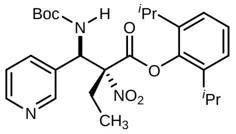

Figure 67. HPLC trace of **13s**

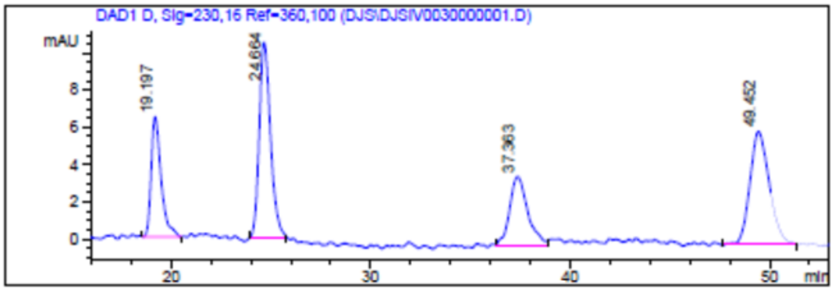

Signal 1: DAD1 D, Sig=230,16 Ref=360,100

Peak #	RT [min]	Width [min]	Area	Area %
1	19.197	0.579	224.281	17.49
2	24.664	0.655	413.658	32.26
3	37.363	1.042	231.462	18.05
4	49.452	1.131	412.803	32.19

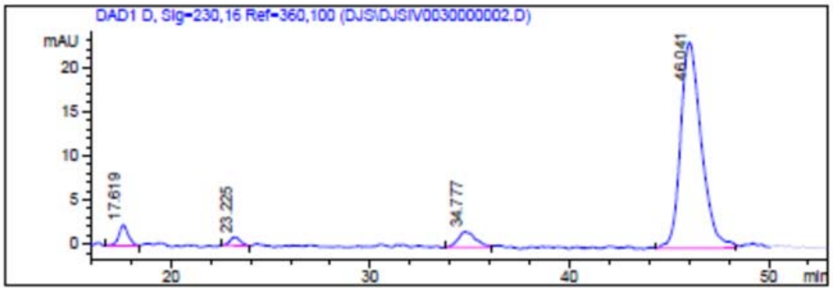

Signal 1: DAD1 D, Sig=230,16 Ref=360,100

Peak #	RT [min]	Width [min]	Area	Area %
1	17.619	0.526	73.799	3.86
2	23.225	0.620	34.850	1.82
3	34.777	1.028	107.879	5.65
4	46.041	1.214	1693.910	88.67

Figure 68. HPLC trace of **13t**

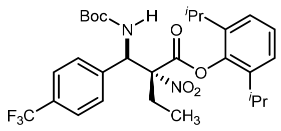

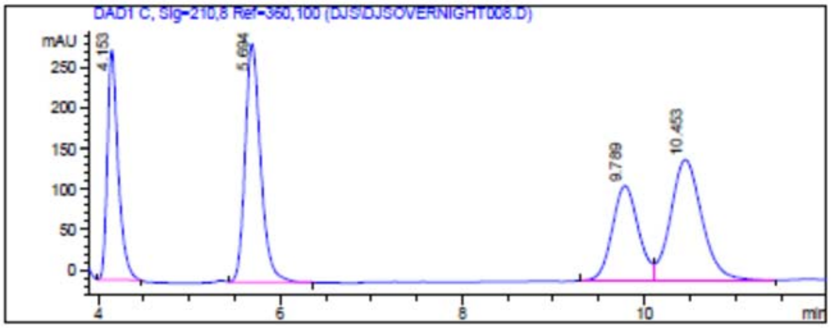

Signal 1: DAD1 C, Sig=210,8 Ref=360,100

Peak #	RT [min]	Width [min]	Area	Area %
1	4.153	0.138	2345.768	19.89
2	5.694	0.198	3497.544	29.65
3	9.789	0.332	2347.997	19.91
4	10.453	0.400	3604.464	30.56

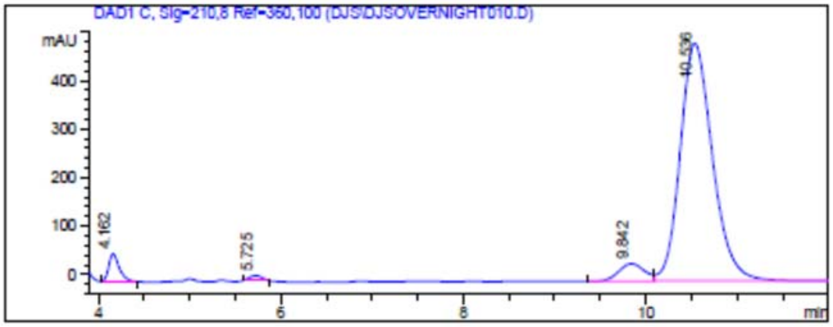

Signal 1: DAD1 C, Sig=210,8 Ref=360,100

Peak #	RT [min]	Width [min]	Area	Area %
1	4.162	0.131	451.034	3.40
2	5.725	0.148	82.500	0.62
3	9.842	0.325	702.720	5.29
4	10.536	0.410	12035.822	90.69

Figure 69. HPLC trace of **13u**[1]

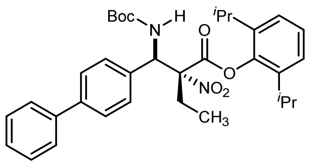

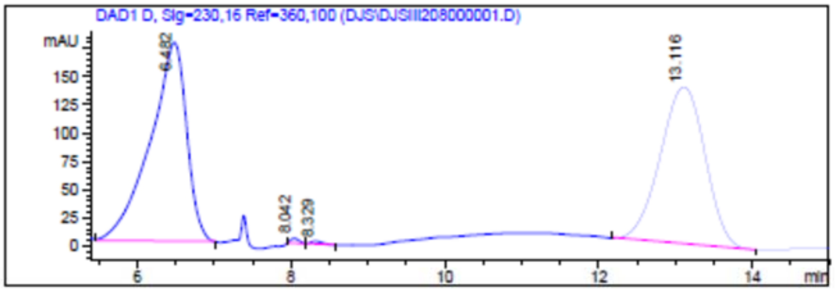

Signal 1: DAD1 D, Sig=230,16 Ref=360,100

Peak #	RT [min]	Width [min]	Area	Area %
1	6.482	0.552	5814.730	50.68
2	8.042	0.126	33.470	0.29
3	8.329	0.166	23.753	0.21
4	13.116	0.675	5602.249	48.82

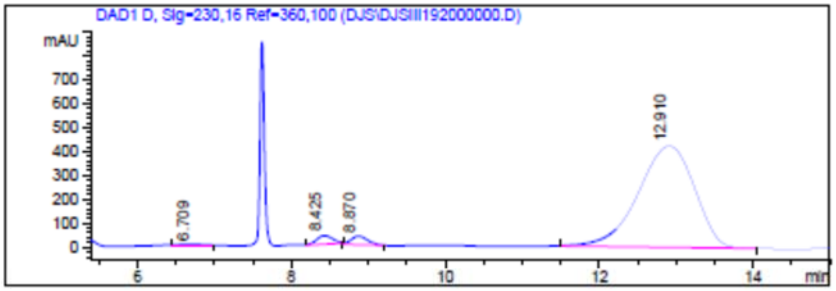

Signal 1: DAD1 D, Sig=230,16 Ref=360,100

Peak #	RT [min]	Width [min]	Area	Area %
1	6.709	0.347	97.340	0.43
2	8.425	0.245	539.446	2.37
3	8.870	0.251	539.384	2.37
4	12.910	0.848	21578.768	94.83

Figure 70. HPLC trace of **SI-1**

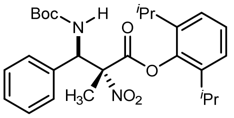

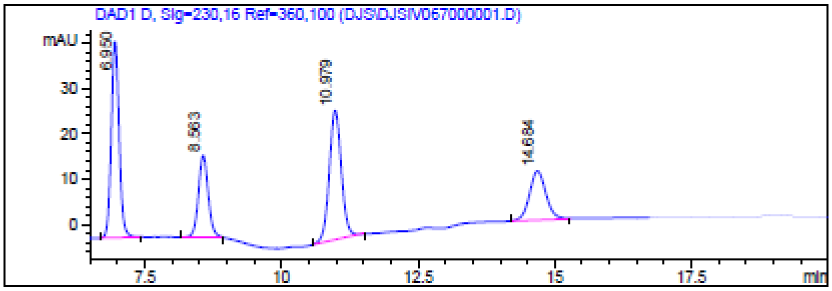

Signal 1: DAD1 D, Sig=230,16 Ref=360,100

Peak #	RT [min]	Width [min]	Area	Area %
1	6.950	0.167	432.810	32.75
2	8.563	0.209	226.393	17.13
3	10.979	0.255	435.921	32.99
4	14.684	0.350	226.234	17.12

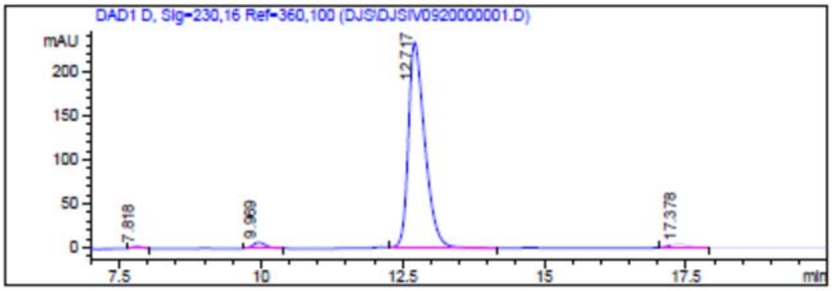

Signal 1: DAD1 D, Sig=230,16 Ref=360,100

Peak #	RT [min]	Width [min]	Area	Area %
1	7.818	0.196	29.793	0.60
2	9.969	0.259	103.128	2.09
3	12.717	0.336	4694.535	95.31
4	17.378	0.388	97.836	1.99
