# Supplementary material for: Diastereo- and enantioselective additions of α-nitro esters to imines for anti-α,β-diamino acid synthesis with α-alkyl-substitution
Source: Chem Sci. 2018 Jan 31;9(8):2336–9. doi: 10.1039/c7sc05176j (PMC5903423; doi:10.1039/c7sc05176j)
Supplement: Supplementary file 2 [file SC-009-C7SC05176J-s002.pdf]

Electronic Supplementary Material (ESI) for Chemical Science.
This journal is © The Royal Society of Chemistry 2018

Diastereo- and Enantioselective Additions of α-Nitro Esters to Imines for *anti*-α,β-Diamino Acid Synthesis with α-Alkyl-Substitution

Daniel J. Sprague, Anand Singh, and Jeffrey N. Johnston*

Department of Chemistry & Vanderbilt Institute of Chemical Biology
Vanderbilt University
2301 Vanderbilt Place, Nashville, TN 37235-1822

SI-I-X

Experimental Section 2
2,6-Diisopropylphenyl 2-nitropent-4-enoate (12e) 2
2,6-Diisopropylphenyl 2-nitro-3-phenylpropanoate (12f) 3
2,6-Diisopropylphenyl 2-cyclopropyl-2-nitroacetate (12g) 3
2,6-Diisopropylphenyl 2-cyclohexyl-2-nitroacetate (12i) 4
General Procedure for the Enantioselective aza-Henry Reaction 5
2,6-Diisopropylphenyl (2*R*,3*R*)-3-((*tert*-butoxycarbonyl)amino)-3-(4-chlorophenyl)-2-methyl-2-nitropropanoate (13a) 5
Diisopropylphenyl (*R*)-2-((*R*)-(4-chlorophenyl)((*tert*-butoxycarbonyl)amino)methyl)-2-nitrobutanoate (13b) 6
2,6-Diisopropylphenyl (*R*)-2-((*R*)-((*tert*-butoxycarbonyl)amino)(4-chlorophenyl)methyl)-2-nitropentanoate (13c) 6
2,6-Diisopropylphenyl (*R*)-2-((*R*)-((*tert*-butoxycarbonyl)amino)(4-chlorophenyl)methyl)-2-nitrohexanoate (13d) 6
2,6-Diisopropylphenyl (*R*)-2-((*R*)-((*tert*-butoxycarbonyl)amino)(4-chlorophenyl)methyl)-2-nitropent-4-enoate (13e) 7
2,6-Diisopropylphenyl (2*R*,3*R*)-2-benzyl-3-((*tert*-butoxycarbonyl)amino)-3-(4-chlorophenyl)-2-nitropropanoate (13f) 7
2,6-Diisopropylphenyl (2*R*,3*R*)-3-((*tert*-butoxycarbonyl)amino)-3-(4-chlorophenyl)-2-cyclopropyl-2-nitropropanoate (13g) 8
2,6-Diisopropylphenyl (*R*)-2-((*R*)-((*tert*-butoxycarbonyl)amino)(4-chlorophenyl)methyl)-3-methyl-2-nitrobutanoate (13h) 8
2,6-Diisopropylphenyl (2*R*,3*R*)-3-((*tert*-butoxycarbonyl)amino)-3-(4-chlorophenyl)-2-cyclohexyl-2-nitropropanoate (13i) 9
2,6-Diisopropylphenyl (*R*)-2-((*R*)-((tert-butoxycarbonyl)amino)(phenyl)methyl)-2-nitrobutanoate (13j) 9
2,6-Diisopropylphenyl (*R*)-2-((*R*)-(4-bromophenyl)((*tert*-butoxycarbonyl)amino)methyl)-2-nitrobutanoate (13k) 10
2,6-Diisopropylphenyl (*R*)-2-((*R*)-((*tert*-butoxycarbonyl)amino)(*m*-tolyl)methyl)-2-nitrobutanoate (13l) 10
2,6-Diisopropylphenyl (*R*)-2-((*R*)-((*tert*-butoxycarbonyl)amino)(3-methoxyphenyl)methyl)-2-nitrobutanoate (13m) 11
2,6-Diisopropylphenyl (*R*)-2-((*R*)-((*tert*-butoxycarbonyl)amino)(4-methoxyphenyl)methyl)-2-nitrobutanoate (13n) 11
2,6-Diisopropylphenyl (*R*)-2-((*S*)-((*tert*-butoxycarbonyl)amino)(furan-2-yl)methyl)-2-nitrobutanoate (13o) 12
2,6-Diisopropylphenyl (*R*)-2-((*S*)-((*tert*-butoxycarbonyl)amino)(thiophen-2-yl)methyl)-2-nitrobutanoate (13p) 12
2,6-Diisopropylphenyl (*R*)-2-((*R*)-((*tert*-butoxycarbonyl)amino)(naphthalen-1-yl)methyl)-2-nitrobutanoate (13q) 12
2,6-Diisopropylphenyl (*R*)-2-((*R*)-((*tert*-butoxycarbonyl)amino)(naphthalen-2-yl)methyl)-2-nitrobutanoate (13r) 13
2,6-Diisopropylphenyl (*R*)-2-((*R*)-((*tert*-butoxycarbonyl)amino)(pyridin-3-yl)methyl)-2-nitrobutanoate (13s) 13
2,6-Diisopropylphenyl (*R*)-2-((*R*)-((*tert*-butoxycarbonyl)amino)(4-(trifluoromethyl)phenyl)methyl)-2-nitrobutanoate (13t) 14
2,6-Diisopropylphenyl (*R*)-2-((*R*)-[1, 1'-biphenyl]-4-yl((*tert*-butoxycarbonyl)amino)methyl)-2-nitrobutanoate (13u) 14
2,6-Diisopropylphenyl (2*R*,3*R*)-2-amino-3-((*tert*-butoxycarbonyl)amino)-2-methyl-3-phenylpropanoate (14) 15
2,6-Diisopropylphenyl (2*R*,3*R*)-3-((*tert*-butoxycarbonyl)amino)-2-methyl-2-nitro-3-phenylpropanoate (SI-1) 15

Experimental Section

All reagents and solvents were commercial grade and purified prior to use when necessary. Toluene and tetrahydrofuran were dried by passage through a column of activated alumina as described by Grubbs.[1] Thin layer chromatography (TLC) was performed using glass-backed silica gel (250 μm) plates and flash chromatography utilized 230–400 mesh silica gel from Sorbent Technologies. UV light, and/or the use of potassium iodoplatinate and potassium permanganate solutions were used to visualize products.

Nuclear magnetic resonance spectra (NMR) were acquired on a Bruker AV-400 (400 MHz) or Bruker DRX-500 (500 MHz) spectrometer. Chemical shifts were measured relative to residual solvent peaks as an internal standard set to δ 7.26 and δ 77.0 ($CDCl_3$).[2] IR spectra were recorded on a Thermo Nicolet IR100 spectrophotometer and are reported in wave numbers (cm^{-1}). Compounds were analyzed as neat films on a NaCl plate (transmission). Mass spectra were recorded on a Waters LCT spectrometer by use of the ionization method noted. A post acquisition gain correction was applied using sodium formate or sodium iodide as the lock mass. Optical rotations were measured on a Perkin Elmer-341 polarimeter.

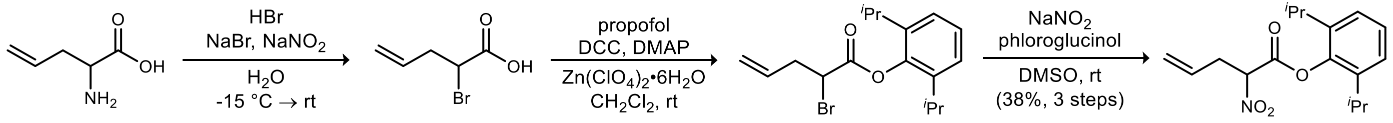

2,6-Diisopropylphenyl 2-nitropent-4-enoate (12e). The α-bromo carboxylic acid was prepared according to literature procedure.[3] The carboxylic acid (7.77 g, 43.4 mmol) was dissolved in dichloromethane (217 mL) and chilled to 0 °C. To the resulting solution was added propofol (7.5 mL, 39 mmol), $Zn(ClO_4)_2$•$6H_2O$ (2.0 g, 5.2 mmol), DMAP (1.1 g, 8.7 mmol) and DCC (10.7 g, 52.1 mmol). The resulting mixture was warmed to ambient temperature and stirred for 24 h. Diethyl ether (250 mL) was added and the reaction mixture was filtered through Celite. The filtrate was washed with 1 M NaOH, 1 M HCl, and brine, and then dried and concentrated. The resulting dark brown oil was passed through a short pad of silica gel (5% ethyl acetate in hexanes) and concentrated to a pale yellow oil.

The oil was dissolved in DMSO (180 mL) and $NaNO_2$ (4.3 g, 62 mmol) and phloroglucinol (4.7 g, 37.2 mmol) were added. The deep red solution was stirred for 24 h and then poured over ice. The ice mixture was warmed to ambient temperature and extracted with Et_2O. The combined organic layers were washed with brine, dried, and concentrated to a dark red oil. Flash column chromatography (SiO_2, 0.5-3% diethyl ether in hexanes) afforded an orange oil contaminated by propofol. This mixture was then subjected to bulb-to-bulb distillation (150 °C, 0.35 torr) which removed further impurities, leaving behind the desired α-nitro ester[4] (4.3 g, 38% yield over 3 steps). R_f = 0.25 (2% Et_2O/Hexanes); IR (film) 3573, 3070, 2963, 2872, 1772, 1645, 1566, 1463, 1365, 794, 749 cm^{-1}; ^1H NMR (400 MHz, $CDCl_3$) δ 7.26 (br dd, J = 8.4, 8.4 Hz, 1H), 7.17 (br d, J = 7.8 Hz, 2H), 5.85 (dddd, J = 13.1, 10.2, 6.7, 6.7 Hz, 1H), 5.46 (dd, J = 9.9, 5.4 Hz, 1H), 5.34 (dddd, J = 17.0, 1.3, 1.3, 1.3 Hz, 2H), 5.29 (dddd, J =

[1] Pangborn, A. B.; Giardello, M. A.; Grubbs, R. H.; Rosen, R. K.; Timmers, F. J. *Organometallics* **1996**, *15*, 1518-1520.
[2] Common in the aza-Henry reaction is hindered rotation at the site of the propofol ester. At times, this leads to magnetically non-equivalent protons and carbons which manifest themselves as unique observable signals in the NMR spectra. It should be noted that in cases of low diastereoselectivity, the carbon signals are reported as a mixture of the two inseparable diastereomers.
[3] Guthrie, D. B.; Geib, S. J.; Curran, D. P. *J. Am. Chem. Soc.* **2009**, *131*, 15492.
[4] The α-nitro ester was contaminated by 8% propofol. Further attempts at purification proved unsuccessful.

10.2, 1.1, 1.1, 1.1 Hz, 1H), 3.24-3.03 m, 2H), 2.95-2.78 (br m, 1H), 1.19 (br d, J = 6.9 Hz, 12H); ^{13}C NMR (100 MHz, $CDCl_3$) ppm 162.8, 144.7, 140.0, 133.6, 129.9, 127.4, 124.2, 123.4, 120.8, 87.2, 34.4, 27.4, 27.1, 23.6, 22.7 (br, 3C); HRMS (ESI): Exact mass calcd for $C_{17}H_{24}NO_4$ $[M+H]^+$ 306.1700, found 306.1702.

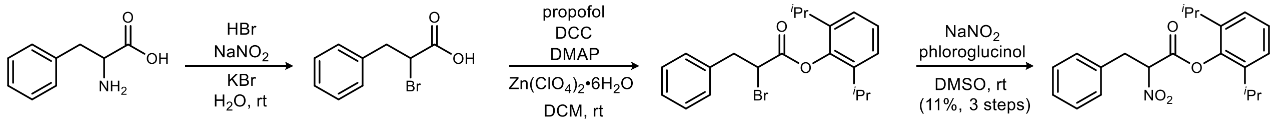

2,6-Diisopropylphenyl 2-nitro-3-phenylpropanoate (12f). According to a known procedure,[5] DL-phenylalanine (2.15 g, 13.0 mmol), $NaNO_2$ (1.88 g, 27.3 mmol), aq HBr (48%, 9.4 mL), and KBr (6.2 g, 52.0 mmol) in H_2O (27 mL) afforded the α-bromo acid as a yellow oil which was used without further purification.

The α-bromo acid was dissolved in CH_2Cl_2 (50 mL) and chilled to 0 °C. To this was added propofol (1.61 mL, 8.67 mmol), $Zn(ClO_4)_2$•$6H_2O$ (323 mg, 867 μmol), DMAP (159 mg, 1.30 mmol), and DCC (2.67 g, 13.0 mmol). The resulting solution was warmed to ambient temperature and stirred for 24 h. The reaction was diluted with Et_2O, filtered through Celite, and concentrated. The residue was dissolved in Et_2O, and washed with 1 N NaOH, 1 N HCl, and brine, and then dried and concentrated. The residue was subjected to MPLC (SiO_2, 0-3% ethyl acetate in hexanes) to afford the α-bromo ester as a pale yellow oil contaminated with 10% of propofol[6] (2.33 g).

This α-bromo ester was dissolved in DMSO (24.0 mL), treated with $NaNO_2$ (711 mg, 10.3 mmol) and phloroglucinol (792 mg, 6.28 mmol), and stirred at ambient temperature for 16 h. The resulting solution was poured over ice, and warmed to ambient temperature. The aqueous layer was extracted with Et_2O, dried, and concentrated. The residue was subjected to flash column chromatography (SiO_2, 0.5-2.0% diethyl ether in hexanes) to afford the desired α-nitro ester as a colorless oil (348 mg, 11% yield over 3 steps). R_f = 0.10, $KMnO_4$ (3% Et_2O/hexanes); IR (film) 2968, 1769, 1562, 1161, 1144 cm^{-1}; ^{1}H NMR (500 MHz, $CDCl_3$) δ 7.40-7.28 (series of br m, 5H), 7.24 (br dd, J = 8.0, 8.0 Hz, 1H), 7.16 (br s, 2H), 5.67 (dd, J = 7.1, 7.1 Hz, 1H), 3.75 (dd, J = 14.4, 8.9 Hz, 1H), 3.65 (dd, J = 14.4, 6.6 Hz, 1H), 2.91 (br s, 1H), 2.48 (br s, 1H), 1.25-1.00 (br m, 12H); ^{13}C NMR (125 MHz, $CDCl_3$) ppm 162.7, 144.7, 140.0 (br), 133.7, 129.1, 129.0, 127.9, 127.4, 124.2, 88.8, 36.3, 27.3, 23.7, 22.7 (2C); HRMS (CI): Exact mass calcd for $C_{21}H_{26}NO_4$ $[M+H]^+$ 356.1856, found 356.1848.

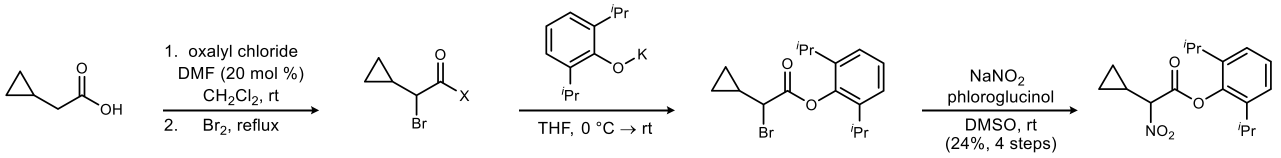

2,6-Diisopropylphenyl 2-cyclopropyl-2-nitroacetate (12g). According to Fu's procedure,[7] a flame-dried flask was charged with the carboxylic acid (1.40 mL, 15.0 mmol), DMF (230 μL, 3.00 mmol), and CH_2Cl_2 (60 mL) and chilled to 0 °C. Oxalyl chloride (1.4 mL, 16 mmol) was added dropwise as gas evolved. The solution was warmed to ambient temperature and stirred for 12 h. Short path distillation of the yellow solution (bath temperature = 75 °C) removed excess oxalyl chloride and solvent, leaving behind the acid chloride in crude form.

---

[5] Kudelko, A. *Tetrahedron* **2012**, *68,* 3616.
[6] At this scale, further separation was not attempted. Purification was more straightforward after nitration.
[7] Dai, X.; Strotman, N. A.; Fu, G. C. *J. Am. Chem. Soc.* **2008**, *130,* 3302.

Bromine (845 µL, 16.5 mmol) was added to the acid chloride and the mixture was refluxed for 4 h. Short path distillation (as above, to remove excess bromine) left behind a mixture of acid halides[8] which were subsequently esterified without further purification or analysis.

A flame-dried flask was charged with KHMDS (2.51 g, 12.6 mmol) and THF (40 mL) and chilled to 0 °C. Propofol (2.22 mL, 12.0 mmol) was added, and the mixture was stirred at 0 °C for 1 h. The mixture of acid halides in THF (50 mL) was added dropwise to the resulting phenolate solution, warmed to ambient temperature and stirred for 12 h. The resulting brown mixture was poured into H_2O and extracted with CH_2Cl_2. The combined organic layers were washed with satd aq $Na_2S_2O_3$, 1 N NaOH, H_2O, and brine. The organic layer was then dried and concentrated. The residue was subjected to MPLC (SiO_2, 0-3% ethyl acetate in hexanes, 80 mL/min) to afford a mixture of α-bromo ester and phenol (3.18 g).[6]

The α-bromo ester was dissolved in DMSO (37.0 mL) and treated with $NaNO_2$ (1.12 g, 16.2 mmol) and phloroglucinol (1.30 g, 10.3 mmol). The mixture was stirred at ambient temperature for 12 h. The resulting dark red solution was poured over ice, warmed to ambient temperature, and extracted with Et_2O. The combined organic layers were washed with brine, dried, and concentrated to yield the crude nitroester as a red oil. Flash column chromatography (SiO_2, 0.5-3% diethyl ether in hexanes) afforded the title compound as a colorless oil (776 mg, 24% yield over 4 steps). R_f = 0.21, $KMnO_4$ (3% Et_2O/hexanes); IR (film) 2968, 1770, 1562, 1162 cm^{-1}; ^{1}H NMR (500 MHz, $CDCl_3$) δ 7.27 (dd, *J* = 7.5, 7.0 Hz, 1H), 7.20 (d, *J* = 7.5 Hz, 2H), 4.57 (d, *J* = 10.5 Hz, 1H), 3.01-2.89 (br m, 2H), 1.97-1.86 (br m, 1H), 1.21 (br d, *J* = 6.7 Hz, 12H), 1.05-0.95 (br m, 2H), 0.80-0.74 (br m, 1H), 0.73-0.68 (br m, 1H); ^{13}C NMR (125 MHz, $CDCl_3$) ppm 163.0, 144.8, 140.1, 127.4, 124.2, 92.5, 27.4 (2C), 23.7, 22.6, 22.5 (2C), 11.8, 5.06, 4.39; HRMS (CI): Exact mass calcd for $C_{17}H_{24}NO_4$ [M+H]$^+$ 306.1700, found 306.1694.

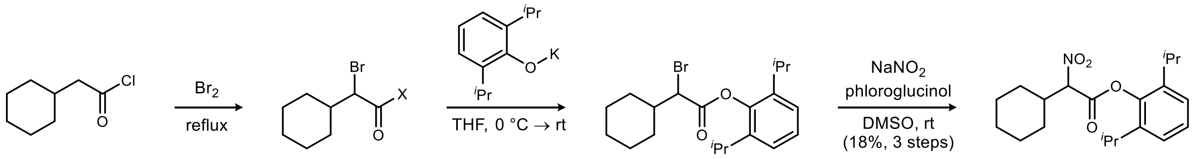

2,6-Diisopropylphenyl 2-cyclohexyl-2-nitroacetate (12i). Bromine (845 µL, 16.5 mmol) was added to the acid chloride (2.30 mL, 15.0 mmol), and the mixture was refluxed for 6 h. Short path distillation (oil bath temperature = 75 °C), to remove excess bromine, left behind a mixture of acid halides[8] which were subsequently esterified without further purification or analysis.

A flame-dried flask was charged with KHMDS (3.30 g, 16.6 mmol) and THF (40 mL) and chilled to 0 °C. Propofol (1.40 mL, 7.50 mmol) was added, and the mixture was stirred at 0 °C for 1 h. The crude mixture of acid halides in THF (40 mL) was added dropwise to the resulting phenolate solution, warmed to ambient temperature, and stirred for 16 h. The resulting brown mixture was poured into H_2O, and extracted with CH_2Cl_2. The combined organic layers were washed with satd aq $Na_2S_2O_3$, 1 N NaOH, H_2O, and brine. The organic layer was then dried and concentrated. The crude residue was subjected to MPLC (SiO_2, 0-3% ethyl acetate in hexanes, 80 mL/min) to afford a mixture of desired bromoester and phenol (1.35 g).[6]

The bromoester was dissolved in DMSO (15.0 mL), treated with $NaNO_2$ (428 mg, 6.20 mmol) and phloroglucinol (469 mg, 3.72 mmol), and stirred at ambient temperature for 16 h. The resulting dark red mixture was poured over ice, warmed to room temperature, and extracted with Et_2O. The combined organic layers were

[8] The acyl bromide and acyl chloride were obtained from this reaction.

washed with brine, dried, and concentrated. The red oil was subjected to flash column chromatography (SiO_2, 0.5-3% diethyl ether in hexanes) to afford the desired α-nitro ester as a colorless oil (469 mg, 18% yield over 3 steps). R_f = 0.33, $KMnO_4$ (3% Et_2O/hexanes); IR (film) 2932, 1769, 1560, 1160, 793 cm^{-1}; ^{1}H NMR (400 MHz, $CDCl_3$) δ 7.28 (br dd, *J* = 7.4, 7.4 Hz, 1H), 7.21 (br d, *J* = 8 Hz, 2H), 5.25 (d, *J* = 8.9 Hz, 1H), 2.93-2.80 (br m, 2H), 2.63-2.48 (br m, 1H), 2.09-1.70 (series of br m, 5H), 1.50-1.28 (series of br m, 5H), 1.22 (br d, *J* = 6.8 Hz, 6H), 1.21 (br d, *J* = 6.8 Hz, 6H); ^{13}C NMR (100 MHz, $CDCl_3$) ppm 162.2, 144.8, 140.3, 127.2, 124.1, 93.1, 38.9, 29.0, 28.7, 27.5, 25.7, 25.5, 25.3, 23.5, 22.8, 22.7; HRMS (CI): Exact mass calcd for $C_{20}H_{30}NO_4$ $[M+H]^+$ 348.2169, found 348.2176.

General Procedure for the Enantioselective aza-Henry Reaction:[9] A flame-dried vial was charged with imine (200 µmol), PBAM•$HNTf_2$ (10 µmol) and toluene (285 µL). The resulting solution was cooled to -20 °C for 30 minutes. The α-nitro ester (220 µmol) was added, and the reaction was stirred at -20 °C for 48 h, then filtered while cold through a short plug of silica gel (ethyl acetate) to remove the catalyst. The filtrate was concentrated and subjected to flash column chromatography which afforded the pure adduct. The diastereomeric ratio was determined by ^{1}H NMR analysis of the crude reaction mixture and the enantiomeric excess was determined by HPLC using a chiral stationary phase.

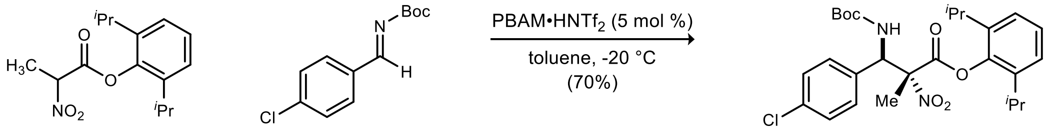

2,6-Diisopropylphenyl (2*R*,3*R*)-3-((*tert*-butoxycarbonyl)amino)-3-(4-chlorophenyl)-2-methyl-2-nitropropanoate (13a). Following the general procedure, the imine (48.0 mg, 200 µmol), ester (61.5 mg, 220 µmol), and catalyst (7.9 mg, 10 µmol) in toluene (285 µL), after flash column chromatography (2-5% ethyl acetate in hexanes) afforded the product as a colorless solid (73 mg, 70% yield) in >20:1 dr and 99% ee; (Chiralcel AD-H, 5% ^{i}PrOH/hexanes, 1 mL/min, t_r(*anti*, minor) = 9.2 min, t_r(*syn*, minor) = 11.2 min, t_r(*anti*, major) = 15.3 min, t_r(*syn*, major) = 28.3 min); mp = 57-60 °C; R_f= 0.42 (10% EtOAc/hexanes); $[\alpha]_D^{25}$ -63 (*c* 1.0, $CHCl_3$); IR (film) 3440, 1751, 1720, 1560, 1491 cm^{-1}; ^{1}H NMR (400 MHz, $CDCl_3$) δ 7.38 (br d, *J* = 8.8 Hz, 2H), 7.35 (br d, *J* = 8.8 Hz, 2H), 7.30-7.18 (br m, 2H), 7.12 (br d, *J* = 6.3 Hz, 1H), 6.47 (br d, *J* = 8.8 Hz, 1H), 5.61 (br d, *J* = 9.6 Hz, 1H), 3.20-3.08 (br m, 1H), 2.04 (s, 3H), 2.00-1.90 (br m, 1H), 1.40 (s, 9H), 1.27 (br d, *J* = 4.4 Hz, 3H), 1.15 (br d, *J* = 6.0 Hz, 3H), 1.02 (br d, *J* = 5.9 Hz, 3H), 0.90 (br d, *J* = 5.9 Hz, 3H); ^{13}C NMR (100 MHz, $CDCl_3$) ppm 164.3, 154.6, 144.7, 140.8, 139.2, 134.9, 133.9, 130.3, 129.1, 127.5, 124.5, 123.8, 94.9, 80.6, 60.0, 28.2, 27.3, 26.6, 24.0, 23.8, 22.8, 22.7, 21.7; HRMS (ESI): Exact mass calcd for $C_{27}H_{35}ClN_2NaO_6$ $[M+Na]^+$ 541.2081, found 541.2085.

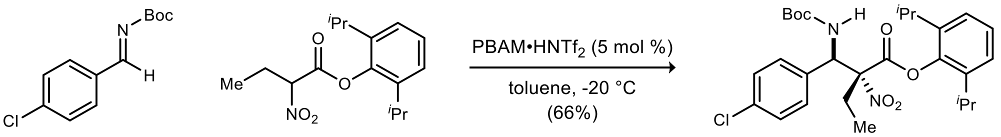

[9] Racemic standards were prepared in the same manner, at ambient temperature with *rac*-PBAM•$HNTf_2$.

Diisopropylphenyl (*R*)-2-((*R*)-(4-chlorophenyl)((*tert*-butoxycarbonyl)amino)methyl)-2-nitrobutanoate (13b). Following the general procedure, the imine (48.0 mg, 200 µmol), ester (64.5 mg, 220 µmol), and catalyst (7.9 mg, 10 µmol) in toluene (285 µL) provided, after flash column chromatography (2-5% ethyl acetate in hexanes), the product as a colorlessv solid (73 mg, 66% yield) in >20:1 dr and 99% ee; (Chiralcel AD-H, 5% ^{i}PrOH/hexanes, 1 mL/min) t_r(*syn*, major) = 4.3 min, t_r(*anti*,minor) = 5.5 min, t_r(*syn*, minor) = 9.2 min, t_r(*anti*, major) = 10.3 min); mp = 77-78 °C; R_f = 0.37 (10% EtOAc/hexanes); $[\alpha]_D^{25}$ +2.8 (*c* 0.40, $CHCl_3$); IR (film) 2972, 2917, 1720, 1562, 1325, 1168, 1130, 668 cm^{-1}; ^1H NMR (400 MHz, $CDCl_3$) δ 7.38-7.16 (series of br m, 6H), 7.10 (br d, *J* = 5.3 Hz, 1H), 6.61 (br d, *J* = 9.2 Hz, 1H), 5.69 (br d, *J* = 9.8 Hz, 1H), 3.24-3.10 (br m, 1H), 2.48-2.30 (m, 2H), 1.76 (br s, 1H), 1.39 (s, 9H), 1.26 (br dd, *J* = 7.1, 7.1 Hz, 3H)[11], 1.15 (br d, *J* = 6.2 Hz, 3H), 1.04 (br d, *J* = 5.6 Hz, 3H), 0.82 (br d, *J* = 5.5 Hz, 3H); ^{13}C NMR (100 MHz, $CDCl_3$) ppm 164.5, 154.6, 144.6, 134.9, 130.1, 129.2, 129.0, 128.8, 127.5, 124.5, 123.7, 98.8, 80.6, 59.3, 29.8, 28.2, 27.3, 26.6, 24.3, 24.2, 22.7, 21.4, 9.4; HRMS (ESI): Exact mass calcd for $C_{28}H_{37}ClN_2NaO_6$ $[M+Na]^+$ 555.2238, found 555.2248.

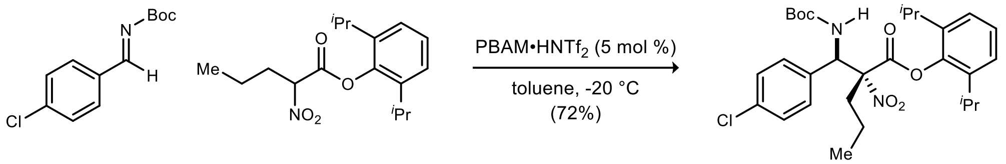

2,6-Diisopropylphenyl (*R*)-2-((*R*)-((*tert*-butoxycarbonyl)amino)(4-chlorophenyl)methyl)-2-nitropentanoate (13c). Following the general procedure, the imine (48.0 mg, 200 µmol), ester (67.6 mg, 220 µmol), and catalyst (7.9 mg, 10 µmol) in toluene (285 µL) provided, after flash column chromatography (2-5% diethyl ether in hexanes), the product as a colorless foamy solid (79 mg, 72% yield) in >20:1 dr and 96% ee; (Chiralpak AD-H, 3% ^{i}PrOH/hexanes, 1 mL/min, t_r(*anti*, minor) = 8.0 min, t_r(*anti*, major) = 9.1 min); mp = 70-72 °C; R_f = 0.39 (10% EtOAc/hexanes); $[\alpha]_D^{25}$ +5.4 (*c* 0.17, $CHCl_3$); IR (film) 3439, 2968, 1745, 1720, 1560, 1491, 1159 cm^{-1}; ^1H NMR (400 MHz, $CDCl_3$) δ 7.39-7.30 (br m, 4H), 7.30-7.16 (series of br m, 2H), 7.10 (br d, *J* = 5.6 Hz, 1H), 6.56 (br d, *J* = 9.4 Hz, 1H), 5.69 (br d, *J* = 9.4 Hz, 1H), 3.21-3.07 (br m, 1H), 2.34-2.17 (br m, 1H), 1.94-1.70 (br m, 2H), 1.39 (s, 9H), 1.31-1.18 (br m, 5H), 1.15 (br d, *J* = 5.9 Hz, 3H), 1.04 (br d, *J* = 4.6 Hz, 3H), 1.00 (dd, *J* = 7.3 Hz, 3H), 0.84 (br d, *J* = 5.0 Hz, 3H); ^{13}C NMR (100 MHz, $CDCl_3$) ppm 164.4, 154.5, 144.6, 140.9, 139.0, 134.9, 134.1, 130.1, 128.9, 128.8, 127.5, 124.6, 123.7, 98.4, 80.6, 59.4, 38.4, 30.3, 29.7, 28.2, 27.2, 26.6, 24.2, 22.7, 21.4, 18.2, 14.1; HRMS (ESI): Exact mass calcd for $C_{29}H_{39}ClN_2NaO_6$ $[M+Na]^+$ 569.2394, found 569.2372.

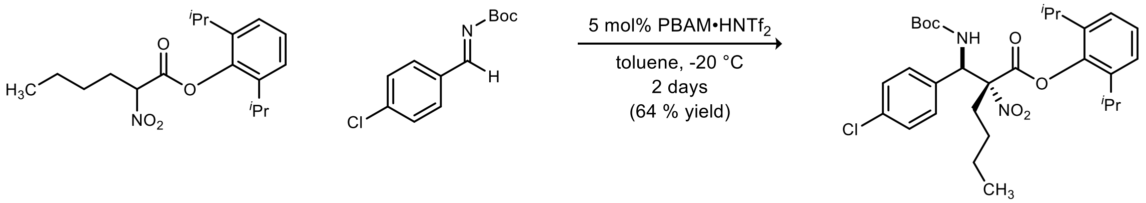

2,6-Diisopropylphenyl (*R*)-2-((*R*)-((*tert*-butoxycarbonyl)amino)(4-chlorophenyl)methyl)-2-nitrohexanoate (13d). Following the general procedure, the imine (48.0 mg, 200 µmol), ester (70.7 mg, 220 µmol), and catalyst (7.9 mg, 10 µmol) in toluene (285 µL), after flash column chromatography (2-5% ethyl acetate in hexanes) afforded the product as a colorless solid (79 mg, 64% yield) in 11:1 dr and 97% ee; (Chiralcel IA, 3% ^{i}PrOH/hexanes, 1 mL/min, t_r(*anti*, major) = 6.5 min, t_r(*anti*, minor) = 5.9 min); mp = 57-60 °C; R_f = 0.13 (3%

EtOAc/Hexanes); $[\alpha]_D^{25}$ -63 (*c* 1.0, $CHCl_3$); IR (film) 3440, 2968, 1720, 1560, 1491, 1159, 1091 cm^{-1}; ^1H NMR (500 MHz, $CDCl_3$) δ 7.40-7.30 (m, 4H), 7.28-7.20 (m, 2H), 7.13-7.06 (m, 1H), 6.60-6.45 (m, 1H), 5.69 (d, J = 9.5 Hz, 1H), 3.20-3.10 (m, 1H), 2.40-2.20 (m, 2H), 1.90-1.70 (m, 1H), 1.40-1.30 (m, 4H), 1.38 (s, 9H), 1.30-1.19 (m, 3H), 1.20-1.15 (m, 3H), 1.10-1.00 (m, 3H), 0.91 (t, J = 7.0 Hz, 3H), 0.85-0.75 (m, 3H); ^{13}C NMR (125 MHz, $CDCl_3$) ppm 164.5, 154.5, 144.6, 140.9, 139.1, 134.9, 134.1, 130.1, 128.8, 127.5, 124.6, 123.7, 98.5, 80.6, 59.3, 36.1, 28.2, 27.2, 26.7, 26.6, 24.3, 22.8, 22.7, 21.5, 13.7; HRMS (ESI): Exact mass calcd for $C_{30}H_{41}ClN_2NaO_6$ [M+Na] 583.2551, found 583.2575.

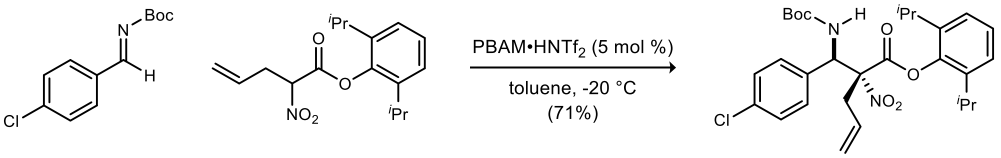

2,6-Diisopropylphenyl (*R*)-2-((*R*)-((*tert*-butoxycarbonyl)amino)(4-chlorophenyl)methyl)-2-nitropent-4-enoate (13e). Following the general procedure, the imine (48.0 mg, 200 µmol), ester (67.2 mg, 220 µmol), and catalyst (7.9 mg, 10 µmol) in toluene (285 µL) provided, after flash column chromatography (3-5% diethyl ether in hexanes), the product as a colorless solid (77 mg, 71% yield) in 9:1 dr and 97% ee; (Chiralpak AD-H, 3% ^{i}PrOH/hexanes, 1 mL/min, t_r(*syn*, major) = 7.6 min, t_r(*anti*, minor) = 10.8 min, t_r(*anti*, major) = 14.2 min, t_r(*syn*, minor) = 24.7 min); mp = 72-74 °C; R_f = 0.56 (10% EtOAc/hexanes); $[\alpha]_D^{25}$ +2.9 (*c* 0.30, $CHCl_3$); IR (film) 3442, 2969, 2932, 1766, 1723, 1562, 1490, 1366, 1340, 1211, 1160, 1093, 1015 cm^{-1}; ^1H NMR (400 MHz, $CDCl_3$) δ 7.36 (s, 4H), 7.29-7.16 (series of br m, 2H), 7.10 (br d, *J* = 5.8 Hz, 1H), 6.59 (br d, *J* = 9.2 Hz, 1H), 6.01 (dddd, *J* = 14.0, 10.2, 7.2, 7.2 Hz, 1H), 5.71 (br d, *J* = 9.8 Hz, 1H), 5.30 (br dd, *J* = 17.2 Hz, 0.88 Hz, 1H), 5.29 (br dd, *J* = 9.8 Hz, 0.6 Hz, 1H), 3.24-3.10 (br m, 1H), 3.05 (br d, *J* = 6.6 Hz, 2H), 1.77 (br s, 1H), 1.38 (s, 9H), 1.26 (br d, *J* = 6.5 Hz, 3H), 1.15 (br d, *J* = 5.8 Hz, 3H), 1.04 (br d, *J* = 5.6 Hz, 3H), 0.82 (br d, *J* = 5.5 Hz, 3H); ^{13}C NMR (100 MHz, $CDCl_3$) ppm 164.1, 154.5, 144.6, 140.8, 139.0, 135.0, 133.8, 130.2, 129.5, 129.3, 129.2, 128.9, 127.5, 124.6, 123.7, 122.2, 98.3, 80.6, 59.3, 40.6, 28.2, 27.3, 26.6, 24.3, 24.2, 22.7, 21.4; HRMS (ESI): Exact mass calcd for $C_{29}H_{37}ClN_2NaO_6$ $[M+Na]^+$ 567.2238, found 567.2224.

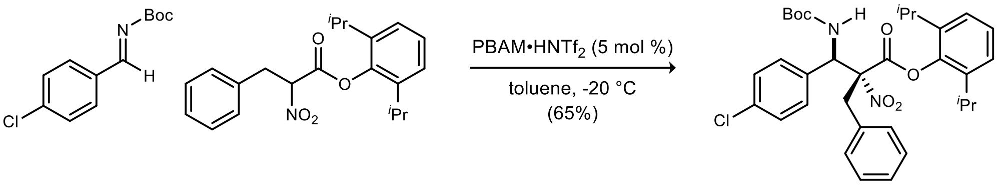

2,6-Diisopropylphenyl (2*R*,3*R*)-2-benzyl-3-((*tert*-butoxycarbonyl)amino)-3-(4-chlorophenyl)-2-nitropropanoate (13f). Following the general procedure, the imine (47.9 mg, 200 µmol), ester (78.2 mg, 220 µmol), and catalyst (7.9 mg, 10 µmol) in toluene (285 µL) provided, after flash column chromatography (2-5% diethyl ether in hexanes), the product as a viscous oil (77 mg, 65% yield)[10] in 4:1 dr and 83% ee; (Chiralcel OD-H, 3% ^{i}PrOH/hexanes, 1 mL/min, t_r(*syn*, major) = 5.2 min, t_r(*anti*, major) = 6.0 min, t_r(*syn*, minor) = 7.7 min, t_r(*anti*, minor) = 9.0 min); R_f = 0.42 (10% EtOAc/hexanes); IR (film) 3432, 2968, 1720, 1561, 1491, 1366, 1160,

[10] Unable to separate the desired adduct from a small amount of residual starting material α-nitro ester.

1093, 698 cm^{-1}; ^1H NMR (400 MHz, $CDCl_3$) δ 7.46-7.06 (series of br m, 12H), 6.66 (br d, *J* = 8.4 Hz, 1H), 5.85 (br d, *J* = 9.2 Hz, 1H), 3.70 (d, *J* = 14.6 Hz, 1H), 3.62 (d, *J* = 14.2 Hz, 1H), 2.46 (br s, 1H), 1.96-1.79 (br m, 1H), 1.41 (s, 9H), 1.36-0.70 (series of br m, 12H); ^{13}C NMR (100 MHz, $CDCl_3$) ppm 169.1, 154.4, 144.0, 135.0, 132.6, 130.3, 130.2, 129.6, 129.1, 129.0, 128.9, 128.5, 128.1, 127.5, 99.0, 80.7, 60.3, 42.0, 28.2, 27.3, 27.1, 26.2, 24.7, 23.8, 21.7; HRMS (ESI): Exact mass calcd for $C_{33}H_{39}ClN_2NaO_6$ $[M+Na]^+$ 617.2394, found 617.2420.

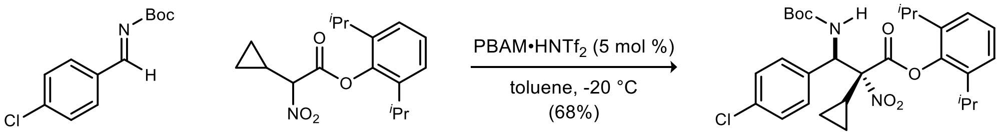

2,6-Diisopropylphenyl (2*R*,3*R*)-3-((*tert*-butoxycarbonyl)amino)-3-(4-chlorophenyl)-2-cyclopropyl-2-nitropropanoate (13g). Following the general procedure, the imine (24.0 mg, 100 µmol), ester (34.0 mg, 110 µmol), and catalyst (4.0 mg, 5 µmol) in toluene (145 µL) provided, after flash column chromatography (SiO_2, 2-5% diethyl ether in hexanes), the product as a colorless viscous oil (37 mg, 68% yield) in 11:1 dr and 98% ee; (Chiralcel OD-H, 2% ^{i}PrOH/hexanes, 0.4 mL/min, t_r(*syn*, minor) = 4.7 min, t_r(*anti*, major) = 5.0 min, t_r(*syn*, major) = 5.5 min, t_r(*anti*, minor) = 7.9 min); R_f = 0.39 (10% EtOAc/hexanes); $[\alpha]_D^{25}$ -6.1 (*c* 0.28, $CHCl_3$); IR (film) 2969, 2929, 1722, 1644, 1561, 1491, 1225, 1161, 1094 cm^{-1}; ^1H NMR (500 MHz, $CDCl_3$) δ 7.44 (br d, *J* = 8.3 Hz, 2H), 7.35 (br d, *J* = 8.6 Hz, 3H), 7.25-7.16 (series of br m, 2H), 7.09 (br d, *J* = 6.5 Hz, 1H), 6.73 (br d, *J* = 9.9 Hz, 1H), 5.76 (br d, *J* = 10.0 Hz, 1H), 3.34-3.23 (br m, 1H), 1.82-1.71 (br m, 1H), 1.26 (br s, 9H), 1.21 (br d, *J* = 6.8 Hz, 2H), 1.16 (br d, *J* = 6.5 Hz, 5H), 1.02 (br d, *J* = 6.2 Hz, 3H), 0.93-0.85 (br m, 2H), 0.76 (br d, *J* = 5.7 Hz, 3H), 0.59-0.50 (br m, 1H); ^{13}C NMR (125 MHz, $CDCl_3$) ppm 164.5, 154.6, 145.2, 140.8, 139.0, 134.8, 134.0, 130.3, 129.8, 128.9, 128.7, 127.4, 124.4, 123.7, 98.5, 80.5, 59.8, 30.3, 29.7, 28.2, 27.4, 26.6, 24.3, 24.0, 22.6, 21.4, 16.5, 5.92, 3.44; HRMS (ESI): Exact mass calcd for $C_{29}H_{37}ClN_2NaO_6$ $[M+Na]^+$ 567.2238, found 567.2224.

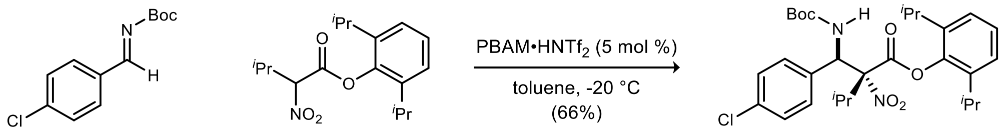

2,6-Diisopropylphenyl (*R*)-2-((*R*)-((*tert*-butoxycarbonyl)amino)(4-chlorophenyl)methyl)-3-methyl-2-nitrobutanoate (13h). Following the general procedure, the imine (48.0 mg, 200 µmol), ester (67.6 mg, 220 µmol), and catalyst (7.9 mg, 10 µmol) in toluene (285 µmol) provided, after flash column chromatography (SiO_2, 1-3% diethyl ether in hexanes), the product as a colorless oil which solidified upon standing (73 mg, 66% yield) in >20:1 dr and 93% ee; (Chiralpak IA, 3% ^{i}PrOH/hexanes, 1 mL/min, t_r(*anti*, major) = 5.1 min, t_r(*anti*, minor) = 6.9 min); mp = 137-138 °C; R_f = 0.61 (10% EtOAc/hexanes); $[\alpha]_D^{25}$ +2.8 (*c* 0.75, $CHCl_3$); IR (film) 2970, 2930, 2871, 1743, 1720, 1556, 1492, 1226, 1160, 1143, 1092 cm^{-1}; ^1H NMR (400 MHz, $CDCl_3$) δ 7.30-7.19 (series of br m, 5H), 7.15 (br d, *J* = 6.2 Hz, 2H), 6.00 (br s, 1H), 5.88 (br d, *J* = 9.0 Hz, 1H), 3.16-3.00 (br m, 1H), 2.86 (br s, 1H), 2.46-2.28 (br m, 1H), 1.41 (s, 9H), 1.36 (d, *J* = 6.8 Hz, 3H), 1.21 (d, *J* = 6.8 Hz, 3H), 1.12 (br d, *J* = 6.5 Hz, 6H), 1.00 (br s, 3H), 0.99 (br s, 3H); ^{13}C NMR (100 MHz, $CDCl_3$) ppm 164.5, 154.6, 144.6, 134.9, 130.1,

129.2, 129.0, 128.8, 127.5, 124.5, 123.7, 98.8, 80.6, 59.3, 29.8, 28.2, 27.3, 26.6, 24.3, 24.2, 22.7, 21.4, 9.4; HRMS (ESI): Exact mass calcd for $C_{29}H_{39}ClN_2NaO_6$ $[M+Na]^+$ 569.2394, found 569.2398.

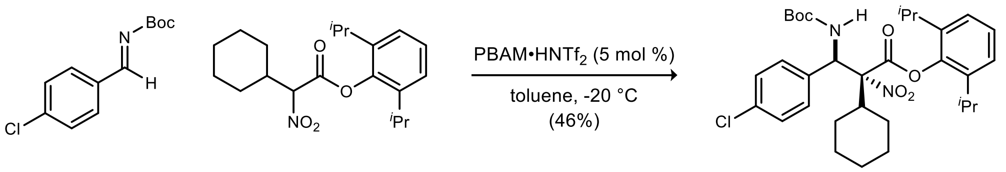

2,6-Diisopropylphenyl (2*R*,3*R*)-3-((*tert*-butoxycarbonyl)amino)-3-(4-chlorophenyl)-2-cyclohexyl-2-nitropropanoate (13i). Following the general procedure, the imine (108 mg, 450 µmol), ester (172 mg, 495 µmol), and catalyst (18.1 mg, 23 µmol) in toluene (650 µL) provided, after flash column chromatography (1-3% ethyl acetate in hexanes), the product as an off-white viscous oil (121 mg, 46% yield) in >20:1 dr and 87% ee; (Chiralcel OZ-H, 2% ^{i}PrOH/hexanes, 0.4 mL/min, t_r(*anti*, minor) = 8.9 min, t_r(*anti*, major) = 10.3 min); R_f = 0.54 (10% EtOAc/hexanes); $[\alpha]_D^{25}$ +3.2 (*c* 0.34, $CHCl_3$); IR (film) 2966, 2930, 2857, 1743, 1720, 1554, 1492, 1366, 1207, 1161, 1092 cm^{-1}; ^1H NMR (400 MHz, $CDCl_3$) δ 7.22-7.12 (series of br m, 5H), 7.07 (br d, *J* = 7.5 Hz, 2H), 5.95-5.73 (br m, 2H), 2.64 (br s, 2H), 2.48 (br s, 1H), 2.05 (br d, *J* = 10.4 Hz, 1H), 1.89-1.75 (br m, 3H), 1.70 (br dd, *J* = 11.4, 8.9 Hz, 2H), 1.36 (br s, 9H), 1.29-0.98 (series of br m, 10H), 0.92 (br s, 6H); ^{13}C NMR (100 MHz, $CDCl_3$) ppm 163.6, 144.8, 135.1 (br, 2C). 134.6, 129.7, 128.7, 127.4, 124.4 (br, 2C), 103.3, 80.8, 55.3, 43.7, 29.7, 28.2, 27.4, 27.1, 26.6, 26.5, 26.0, 24.3, 22.7; HRMS (ESI): Exact mass calcd for $C_{32}H_{43}ClN_2NaO_6$ $[M+Na]^+$ 609.2707, found 609.2735.

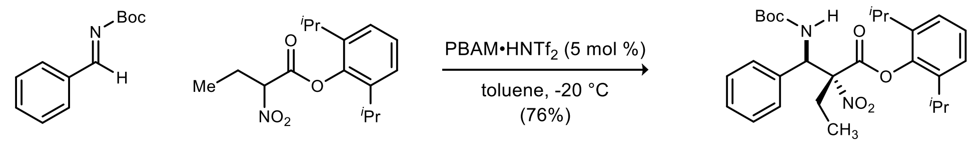

2,6-Diisopropylphenyl (*R*)-2-((*R*)-((tert-butoxycarbonyl)amino)(phenyl)methyl)-2-nitrobutanoate (13j). Following the general procedure, the imine (41.1 mg, 200 µmol), ester (64.5 mg, 220 µmol), and catalyst (7.9 mg, 10 µmol) in toluene (285 µmol) provided, after flash column chromatography (2-10% ethyl acetate in hexanes), the product as an amorphous solid (66 mg, 76% yield) in >20:1 dr and 96% ee; (Chiralpak IA, 3% ^{i}PrOH/hexanes, 1 mL/min, t_r(*syn*, minor) = 5.0 min, t_r(*anti*, minor) = 6.5 min, t_r(*syn*, major) = 7.6 min, t_r(*anti*, major) = 9.7 min); mp = 66-68 °C; R_f = 0.44 (10% EtOAc/hexanes); $[\alpha]_D^{25}$ +3.3 (*c* 0.67, $CHCl_3$); IR (film) 2972, 2917, 1720, 1562, 1325, 1168, 1130, 668 cm^{-1}; ^1H NMR (500 MHz, DMSO-d_6) δ 7.73 (br d, *J* = 10.5 Hz, 1H), 7.44 (br s, 2H), 7.36 (br s, 3H), 7.31-7.15 (br m, 3H), 5.79 (br d, *J* = 9.5 Hz, 1H), 2.85 (br s, 1H), 2.48-2.39 (br m, 2H), 2.30 (br s, 1H), 1.32 (s, 9H), 1.15-0.97 (br m, 12H), 0.93 (br s, 3H); ^{13}C NMR (125 MHz, DMSO-d_6) ppm 163.4, 154.8, 144.2, 135.9, 128.9, 128.5, 128.2, 127.4, 124.3, 99.6, 79.3, 65.0, 58.2, 28.0, 26.9, 26.5 (2C), 23.9 (2C), 22.6, 22.3, 15.2, 8.7; HRMS (ESI): Exact mass calcd for $C_{28}H_{38}NaN_2O_6$ $[M+Na]^+$ 521.2628, found 521.2609.

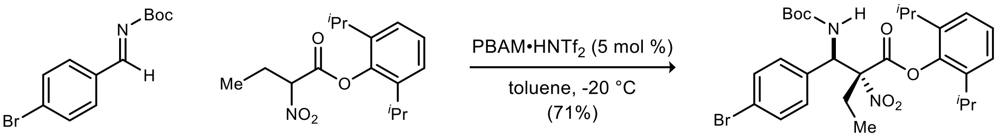

2,6-Diisopropylphenyl (*R*)-2-((*R*)-(4-bromophenyl)((*tert*-butoxycarbonyl)amino)methyl)-2-nitrobutanoate (13k). Following the general procedure, the imine (56.8 mg, 200 μmol), ester (64.5 mg, 220 μmol), and catalyst (7.9 mg, 10 μmol) in toluene (285 μmol) provided, after flash column chromatography (2-5% diethyl ether in hexanes), the product as a colorless solid (82 mg, 71% yield) in >20:1 dr and 99% ee; (Chiralpak IA, 3% ^{i}PrOH/hexanes, 1 mL, /min) t_r(*anti*, major) = 4.7 min, t_r(*syn*, minor) = 10.9, t_r(*anti*, minor) = 17.8 min, t_r(*syn*, major) = 27.3 min); mp = 73-74 °C; R_f = 0.37 (10% EtOAc/hexanes); $[\alpha]_D^{25}$ -3.2 (*c* 0.34, $CHCl_3$); IR (film) 2968, 2929, 1271, 1641, 1561, 1489, 1366, 1225, 1160 cm^{-1}; ^{1}H NMR (400 MHz, $CDCl_3$) δ 7.49 (br d, *J* = 8.5 Hz, 2H), 7.27 (br d, *J* = 5.3 Hz, 2H), 7.24 (br d, *J* = 8.6 Hz, 2H), 7.09 (br d, *J* = 6.0 Hz, 1H), 6.59 (br d, *J* = 9.6 Hz, 1H), 5.66 (br d, *J* = 10.0 Hz, 1H), 3.24-3.08 (br m, 1H), 2.46-2.28 (br m, 2H), 1.75 (br s, 1H), 1.38 (s, 9H), 1.24 (br dd, *J* = 7.2, 7.2 Hz, 3H)[11], 1.14 (br d, *J* = 6.2 Hz, 3H), 1.03 (br d, *J* = 5.5 Hz, 3H), 0.81 (br d, *J* = 5.5 Hz, 3H); ^{13}C NMR (100 MHz, $CDCl_3$) ppm 164.5, 154.5, 144.6, 140.9, 139.1, 134.6, 131.8, 130.4, 127.5. 124.6, 123.7, 123.1, 98.8, 80.6, 59.4, 29.8, 28.2, 27.3, 24.3, 24.2, 22.7, 21.4, 9.4; HRMS (ESI): Exact mass calcd for $C_{28}H_{37}BrN_2NaO_6$ $[M+Na]^+$ 599.1733, found 599.1761.

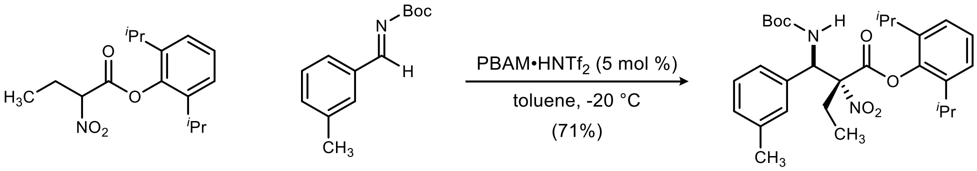

2,6-Diisopropylphenyl (*R*)-2-((*R*)-((*tert*-butoxycarbonyl)amino)(*m*-tolyl)methyl)-2-nitrobutanoate (13l). Following the general procedure, the imine (43.9 mg, 200 μmol), ester (64.5 mg, 220 μmol), and catalyst (7.9 mg, 10 μmol) in toluene (285 μmol), after flash column chromatography (2-5% ethyl acetate in hexanes) afforded the product as a colorless solid (73 mg, 71% yield) in 12:1 dr and 97% ee; (Chiralcel AD-H, 3% ^{i}PrOH/hexanes, 1 mL/min, t_r(*syn*, major) = 4.4 min, t_r(*anti*, minor) = 5.6 min), t_r(*syn*, minor) = 6.4 min, t_r(*anti*, major) = 7.9 min); mp = 48-50 °C; R_f = 0.38 (10% EtOAc/hexanes); $[\alpha]_D^{25}$ -34 (*c* 1.0, $CHCl_3$); IR (film) 3442, 2968, 1747, 1723, 1559, 1490, 1220, 1160 cm^{-1}; ^{1}H NMR (500 MHz, $CDCl_3$) δ 7.30-7.10 (series of m, 6H), 7.10-7.00 (br m, 1H), 6.65 (br d, *J* = 9.1 Hz, 1H), 5.67 (br d, *J* = 9.5 Hz, 1H), 3.30-3.15 (br m, 1H), 2.43-2.36 (br m, 2H), 2.35 (s, 3H), 1.90-1.70 (br s, 1H), 1.39 (s, 9H), 1.35-1.20 (br m, 6H), 1.15 (br d, *J* = 6.1 Hz, 3H), 1.05-0.95 (br s, 3H), 0.80-0.74 (br s, 3H); ^{13}C NMR (125 MHz, $CDCl_3$) ppm 164.5, 154.6, 144.7, 139.2, 138.3, 135.3, 129.5, 128.5, 127.3, 125.6, 124.5, 123.6, 99.2, 80.2, 60.0, 29.9, 28.2, 27.3, 27.1, 26.6, 24.2, 22.7, 21.5, 9.44; HRMS (ESI): Exact mass calcd for $C_{29}H_{40}N_2NaO_6$ $[M+Na]^+$ 535.2784, found 535.2778.

---

[11] One methyl signal arising from the aryl ^{i}Pr groups is eclipsed by the dd.

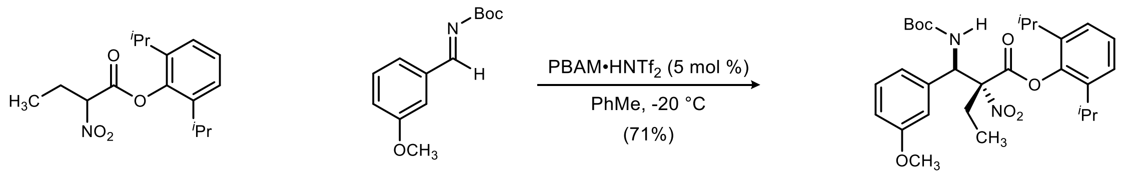

2,6-Diisopropylphenyl (*R*)-2-((*R*)-((*tert*-butoxycarbonyl)amino)(3-methoxyphenyl)methyl)-2-nitrobutanoate (13m). Following the general procedure, the imine (47.1 mg, 200 µmol), ester (64.5 mg, 220 µmol), and catalyst (7.9 mg, 10 µmol) in toluene (285 µmol) provided, after flash column chromatography (3-10% ethyl acetate in hexanes), the product as a colorless viscous oil (75 mg, 71% yield) in 5:1 dr and 96% ee. (Chiralcel AD-H, 5% ^{i}PrOH/hexanes, 1 mL/min, t_r(*syn*, major) = 4.6 min, t_r(*anti*, minor) = 6.3 min, t_r(*syn*, minor) = 6.7 min, t_r(*anti*, major) = 11.2 min); R_f = 0.21 (10% EtOAc/hexanes); $[\alpha]_D^{25}$ +6.9 (*c* 0.35, $CHCl_3$); IR (film) 3441, 2971, 1747, 1722, 1602, 1560, 1489, 1366, 1221, 1159 cm^{-1}; ^{1}H NMR (400 MHz, $CDCl_3$) δ 7.30-7.15 (series of br m, 3H), 7.07 (br d, *J* = 5.6 Hz, 1H), 6.99-6.86 (series of br m, 3H), 6.66 (br d, *J* = 9.8 Hz, 1H), 5.67 (br d, *J* = 9.6 Hz, 1H), 3.77 (s, 3H), 3.28-3.14 (br m, 1H), 2.47-2.30 (br m, 2H), 1.90-1.76 (br m, 1H), 1.38 (s, 9H), 1.32-1.20 (series of m, 6H), 1.15 (br d, *J* = 5.5 Hz, 3H), 0.99 (br d, *J* = 5.9 Hz, 3H), 0.78 (br d, *J* = 5.8 Hz, 3H); ^{13}C NMR (100 MHz, $CDCl_3$) ppm 164.5, 159.7, 159.6, 154.6, 144.8 (2C), 136.9, 129.6, 127.3, 124.5, 123.6, 120.9, 114.8, 113.8, 99.0, 80.3, 59.9, 55.2, 29.9, 28.2 (3C), 27.3 (2C), 26.6, 24.3, 22.8, 21.4, 9.45; HRMS (ESI): Exact mass calcd for $C_{29}H_{40}N_2NaO_7$ $[M+Na]^+$ 551.2733, found 551.2755.

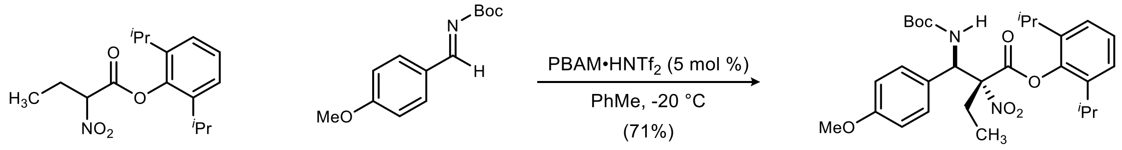

2,6-Diisopropylphenyl (*R*)-2-((*R*)-((*tert*-butoxycarbonyl)amino)(4-methoxyphenyl)methyl)-2-nitrobutanoate (13n). Following the general procedure, the imine (47.1 mg, 200 µmol), ester (64.5 mg, 220 µmol), and catalyst (7.9 mg, 10 µmol) in toluene (285 µmol) provided, after flash column chromatography (3-10% ethyl acetate in hexanes), the product as a colorless viscous oil (75 mg, 71% yield) in 5:1 dr and 78% ee. (Chiralcel AD-H, 5% ^{i}PrOH/hexanes, 1 mL/min, t_r(*syn*, major) = 4.6 min, t_r(*anti*, minor) = 6.3 min, t_r(*syn*, minor) = 6.7 min, t_r(*anti*, major) = 11.2 min); R_f=0.36 (20% EtOAc/hexanes); IR (film) 3456, 2968, 2932, 1765, 1722, 1557, 1514, 1483 cm^{-1}; ^{1}H NMR (400 MHz, $CDCl_3$) δ 7.28-7.23 (m, 1H), 7.19-7.16 (m, 4H), 6.87 (d, *J* = 8.7 Hz, 2H), 6.49 (d, *J* = 9.8 Hz, 1H), 5.57 (d, *J* = 9.9 Hz, 1H), 3.80 (s, 3H), 3.06 (br s, 1H), 2.86 (br s, 1H), 2.45-2.36 (m, 1H), 2.06 (dq, *J* = 15.0, 7.5 Hz, 1H), 1.37 (s, 9H), 1.25-1.20 (m, 3H), 1.22 (d, *J* = 6.9 Hz, 12H); ^{13}C NMR (100 MHz, $CDCl_3$) ppm 163.6, 159.9, 154.7, 144.6, 130.0, 128.8, 127.5, 127.4, 124.2, 114.4, 99.4, 80.2, 55.7, 55.3, 28.4, 27.4, 27.2, 23.9 (2C), 8.7. HRMS (CI): Exact mass calculated for $C_{29}H_{41}N_2O_7$ $[M+H]^+$ 529.2914, found 529.2908.

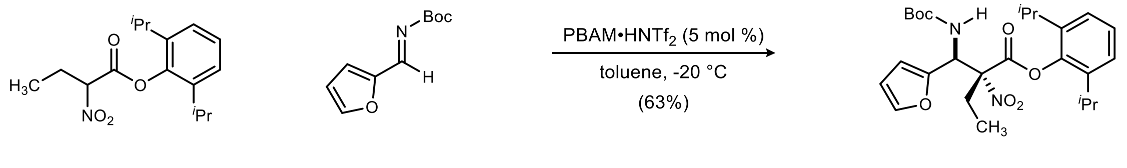

2,6-Diisopropylphenyl (*R*)-2-((*S*)-((*tert*-butoxycarbonyl)amino)(furan-2-yl)methyl)-2-nitrobutanoate (13o).[12] Following the general procedure, the imine (39.0 mg, 200 µmol), ester (64.5 mg, 220 µmol), and catalyst (7.9 mg, 10 µmol) in toluene (285 µL), after flash column chromatography (2-5% ethyl acetate in hexanes) afforded the product as a colorless viscous oil (64 mg, 63% yield) in 4:1 dr and 91% ee; (Chiralcel AD-H, 3% ^{i}PrOH/hexanes, 1 mL/min, t_r(*syn*, minor) = 7.2 min t_r(*anti*, major) = 8.6 min, t_r(*anti*, minor) = 9.1 min, t_r(*syn*, major) = 9.8 min); R_f = 0.42 (10% EtOAc/hexanes); IR (film) 3440, 1724, 1560, 1488, 1226, 1158, 1091 cm^{-1}; ^{1}H NMR (400 MHz, $CDCl_3$) δ 7.41 (s, 1H), 7.30-7.10 (series of br m, 4H), 6.40 (s, 1H), 6.38 (br d, *J* = 10.8 Hz, 1H), 5.87 (br d, *J* = 10.1 Hz, 1H), 3.30-3.16 (br s, 1H), 2.60-2.50 (br m, 1H), 2.48-2.38 (br m, 1H), 2.37-2.28 (br m, 1H), 1.42 (s, 9H), 1.27 (dd, *J* = 4.4, 4.4 Hz, 3H), 1.23-1.10 (series of br m, 9H), 1.04-0.98 (br s, 3H); ^{13}C NMR (100 MHz, $CDCl_3$) ppm 164.4, 154.7, 149.4, 144.7, 142.8, 140.8, 139.7, 127.4, 124.4, 110.8, 110.0, 97.7, 80.5, 53.5, 29.0, 28.2, 27.1, 26.9, 24.3, 22.8, 22.1, 8.84; HRMS (ESI): Exact mass calcd for $C_{26}H_{36}N_2NaO_7$ [M+Na]$^+$

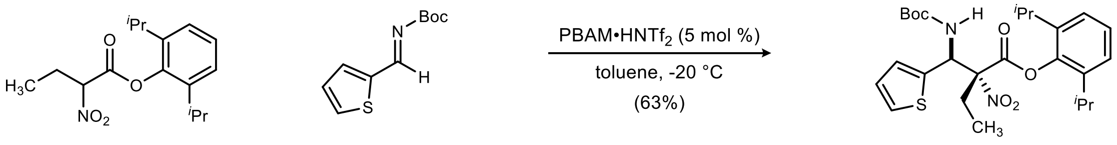

511.2420, found 511.2444.

2,6-Diisopropylphenyl (*R*)-2-((*S*)-((*tert*-butoxycarbonyl)amino)(thiophen-2-yl)methyl)-2-nitrobutanoate (13p). Following the general procedure, the imine (42.7 mg, 200 µmol), ester (64.5 mg, 220 µmol), and catalyst (7.9 mg, 10 µmol) in toluene (285 µL), after flash column chromatography (1.5-4% ethyl acetate in hexanes) afforded the product as a colorless solid (64 mg, 63% yield) in >20:1 dr and 97% ee; (Chiralcel AD-H, 3% ^{i}PrOH/hexanes, 1 mL/min, t_r (*syn*, minor) = 4.9 min, t_r(*anti*, minor) = 6.5 min, t_r(*syn*, major) = 7.0 min, t_r(*anti*, major) = 8.1 min); mp = 103-106 °C; R_f = 0.33 (10% EtOAc/hexanes); $[\alpha]_D^{25}$ -17 (*c* 0.51, $CHCl_3$); IR (film) 3433, 1746, 1722, 1560, 1487, 1366, 1225, 1159 cm^{-1}; ^{1}H NMR (400 MHz, $CDCl_3$) δ 7.32 (dd, *J* = 5.0, 0.72 Hz, 1H), 7.29-7.16 (br m, 2H), 7.11 (br d, *J* = 3.5 Hz, 2H), 7.01 (dd, *J* = 3.7, 1.4 Hz, 1H), 6.51 (br d, *J* = 9.4 Hz, 1H), 5.96 (br d, *J* = 9.8 Hz, 1H), 3.25-3.15 (br m, 1H), 2.50 (m, 2H), 2.05 (br s, 1H), 1.42 (s, 9H), 1.29 (dd, *J* = 7.2, 7.2 Hz, 3H), 1.27-1.20 (br m, 3H), 1.17 (br d, *J* = 4.6 Hz, 3H), 1.06 (br d, *J* = 4.9 Hz, 3H), 0.93 (br d, *J* = 5.3 Hz, 3H); ^{13}C NMR (100 MHz, $CDCl_3$) ppm 164.6, 154.5, 144.7, 140.9, 139.4, 138.5, 127.8, 127.4, 126.9, 126.2, 124.4, 123.8, 98.5, 80.5, 55.9, 29.8, 28.2, 27.2, 26.6, 24.4, 24.1, 22.8, 21.9, 9.15; HRMS (ESI): Exact mass calcd for $C_{26}H_{36}N_2NaO_6S$ [M+Na]$^+$ 527.2192, found 527.2192.

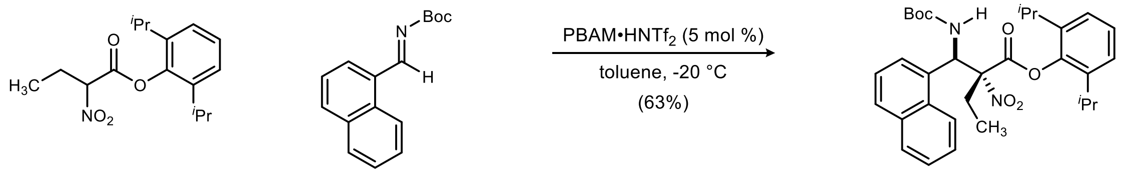

2,6-Diisopropylphenyl (*R*)-2-((*R*)-((*tert*-butoxycarbonyl)amino)(naphthalen-1-yl)methyl)-2-nitrobutanoate (13q). Following the general procedure, the imine (42.7 mg, 200 µmol), ester (64.5 mg, 220 µmol), and catalyst (7.9 mg, 10 µmol) in toluene (285 µL), after flash column chromatography (1.5-4% ethyl acetate in hexanes)

[12] ^{13}C NMR signals are reported as a combination of a mixture of the two inseparable diastereomers.

afforded the product as a colorless solid (64 mg, 63% yield) in >20:1 dr and 97% ee; (Chiralcel AD-H, 3% ^{i}PrOH/hexanes, 1 mL/min, t_r (*syn*, minor) = 4.9 min, t_r(*anti*, minor) = 6.5 min, t_r(*syn*, major) = 7.0 min, t_r(*anti*, major) = 8.1 min); mp = 103-106 °C; R_f = 0.33 (10% EtOAc/hexanes); $[\alpha]_D^{25}$ -17 (*c* 0.51, $CHCl_3$); IR (film) 3433, 1746, 1722, 1560, 1487, 1366, 1225, 1159 cm^{-1}; ^{1}H NMR (400 MHz, $CDCl_3$) δ 7.32 (dd, *J* = 5.0, 0.72 Hz, 1H), 7.29-7.16 (br m, 2H), 7.11 (br d, *J* = 3.5 Hz, 2H), 7.01 (dd, *J* = 3.7, 1.4 Hz, 1H), 6.51 (br d, *J* = 9.4 Hz, 1H), 5.96 (br d, *J* = 9.8 Hz, 1H), 3.25-3.15 (br m, 1H), 2.50 (m, 2H), 2.05 (br s, 1H), 1.42 (s, 9H), 1.29 (dd, *J* = 7.2, 7.2 Hz, 3H), 1.27-1.20 (br m, 3H), 1.17 (br d, *J* = 4.6 Hz, 3H), 1.06 (br d, *J* = 4.9 Hz, 3H), 0.93 (br d, *J* = 5.3 Hz, 3H); ^{13}C NMR (100 MHz, $CDCl_3$) ppm 164.6, 154.5, 144.7, 140.9, 139.4, 138.5, 127.8, 127.4, 126.9, 126.2, 124.4, 123.8, 98.5, 80.5, 55.9, 29.8, 28.2, 27.2, 26.6, 24.4, 24.1, 22.8, 21.9, 9.15; HRMS (ESI): Exact mass calcd for $C_{26}H_{36}N_2NaO_6S$ $[M+Na]^+$ 527.2192, found 527.2192.

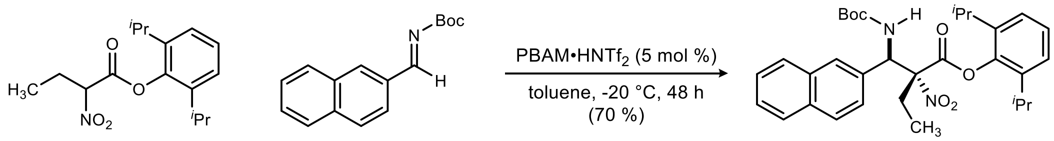

2,6-Diisopropylphenyl (*R*)-2-((*R*)-((*tert*-butoxycarbonyl)amino)(naphthalen-2-yl)methyl)-2-nitrobutanoate (13r). Following the general procedure, the imine (42.7 mg, 200 μmol), ester (64.5 mg, 220 μmol), and catalyst (7.9 mg, 10 μmol) in toluene (285 μmol) provided, after flash column chromatography (2-5% ethyl acetate in hexanes), the product as a colorless solid (76 mg, 70% yield) in >20:1 dr and 96% ee; (Chiralcel IA, 5% ^{i}PrOH/hexanes, 1 mL/min, t_r(*syn*, minor) = 4.8 min), t_r(*anti*, minor) = 5.7 min, t_r(*syn*, major) = 7.4 min, t_r(*anti*, major) = 10.8 min); mp = 66-68 °C; R_f = 0.42 (10% EtOAc/hexanes); $[\alpha]_D^{25}$ -23 (*c* 0.13, $CHCl_3$); IR (film) 3445, 1747, 1720, 1559, 1490, 1160 cm^{-1}; ^{1}H NMR (400 MHz, $CDCl_3$) δ 7.90-7.80 (series of br m, 4H), 7.56-7.46 (m, 3H), 7.23-7.16 (br m, 2H), 7.02-6.90 (br m, 1H), 6.79 (br d, *J* = 9.6 Hz, 1H), 5.89 (br d, *J* = 9.6 Hz, 1H), 3.30-3.10 (br m, 1H), 2.60-2.35 (br m, 2H), 1.55-1.45 (br m, 1H), 1.39 (s, 9H), 1.28 (dd, *J* = 7.2, 7.2 Hz, 3H), 1.26-1.21 (m, 3H), 1.13 (d, *J* = 6.2 Hz, 3H), 0.54 (br d, *J* = 7.0 Hz, 3H), 0.51 (br d, *J* = 6.8 Hz, 3H); ^{13}C NMR (100 MHz, $CDCl_3$) ppm 164.6, 154.6, 144.7, 140.9, 139.1, 133.3, 133.0, 133.7, 128.5, 128.4, 128.2, 127.5, 127.3, 126.8, 126.5, 125.9, 124.5, 123.6, 99.2, 80.3, 60.1, 30.1, 28.2, 27.2, 26.6, 24.2, 24.0, 22.7, 20.7, 9.48; HRMS (ESI): Exact mass calcd for $C_{32}H_{40}N_2NaO_6$ $[M+Na]^+$ 571.2784, found 571.2785.

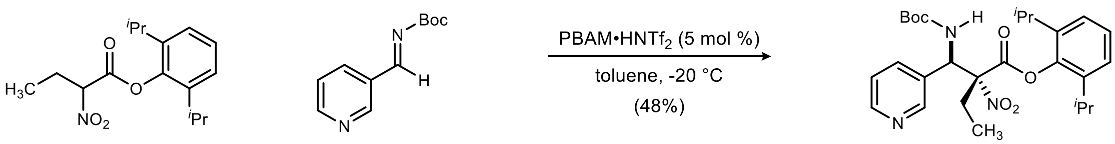

2,6-Diisopropylphenyl (*R*)-2-((*R*)-((*tert*-butoxycarbonyl)amino)(pyridin-3-yl)methyl)-2-nitrobutanoate (13s). Following the general procedure, the imine (20.6 mg, 100 μmol), ester (32.3 mg, 110 μmol), and catalyst (4.0 mg, 5.0 μmol) in toluene (143 μL), after flash column chromatography (20% ethyl acetate in hexanes) afforded the product as a colorless solid (24 mg, 48% yield) in 9:1 dr and 96% ee; (Chiralcel AD-H, 3% ^{i}PrOH/hexanes, 1 mL/min, t_r(*syn*, minor) = 17.6 min , t_r(*anti*, minor) = 23.2 min, t_r(*anti*, major) = 34.8 min, t_r(*syn*, major) = 46.0 min); mp 52-54 °C; R_f = 0.14 (20% EtOAc/hexanes); $[\alpha]_D^{25}$ -18 (*c* 0.45, $CHCl_3$); IR (film) 3442, 1747, 1723, 1559, 1490, 1220, 1160 cm^{-1}; ^{1}H NMR (500 MHz, $CDCl_3$) δ 8.68 (br s, 1H), 8.62 (br d, *J* = 3.5 Hz,

1H), 7.71 (br d, J = 7.9 Hz, 1H), 7.35-7.20 (series of br m, 3H), 7.10 (br s, 1H), 6.53 (br s, 1H), 5.74 (br d, J = 9.7 Hz, 1H), 3.13 (br s, 1H), 2.50-2.30 (series of br m, 2H), 1.90-1.80 (br s, 1H), 1.39 (s, 9H), 1.30-1.20 (series of m, 6H), 1.18-1.15 (br s, 3H), 1.05-0.95 (br s, 3H), 0.85-0.75 (br s, 3H); ^{13}C NMR (125 MHz, $CDCl_3$) ppm 164.5, 154.5, 150.3, 150.0, 144.5, 140.8, 139.1, 136.1, 131.6, 127.5, 124.6, 123.8, 123.4, 98.8, 80.8, 57.7, 29.6, 28.2, 27.3, 27.2, 24.1, 22.8, 21.9, 9.25; HRMS (ESI): Exact mass calcd for $C_{27}H_{38}N_3O_6$ [M+H]$^+$ 500.2761, found 500.2776.

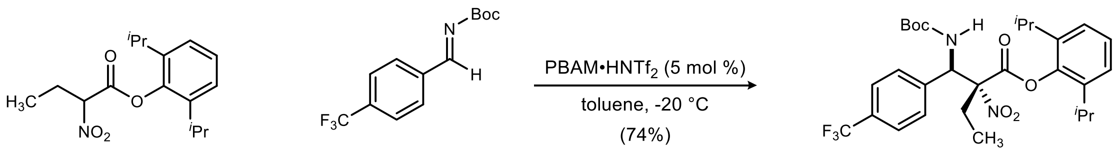

2,6-Diisopropylphenyl (*R*)-2-((*R*)-((*tert*-butoxycarbonyl)amino)(4-(trifluoromethyl)phenyl)methyl)-2-nitrobutanoate (13t). Following the general procedure, the imine (54.7 mg, 200 µmol), ester (64.5 mg, 220 µmol), and catalyst (7.9 mg, 10 µmol) in toluene (285 µmol) provided, after flash column chromatography (2-5% ethyl acetate in hexanes), the product as a colorless solid (84 mg, 74% yield) in 15:1 dr and 99% ee; (Chiralcel AD-H, 5% ^{i}PrOH/hexanes, 1 mL/min, t_r(*anti*, minor) = 5.3 min. t_r(*syn*, major and minor)[13] = 7.3 min, t_r(*anti*, major) = 9.6 min); mp = 55-58 °C; R_f = 0.42 (10% EtOAc/hexanes); $[\alpha]_D^{25}$ +3.3 (c 0.67, $CHCl_3$); IR (film) 2972, 2917, 1720, 1562, 1325, 1168, 1130, 668 cm^{-1}; ^{1}H NMR (400 MHz, $CDCl_3$) δ 7.64 (d, J = 8.3 Hz, 2H), 7.55 (d, J = 8.2 Hz, 2H), 7.30-7.15 (series of br m, 2H), 7.11 (br s, 1H), 6.65 (br d, J = 9.3 Hz, 1H), 5.78 (br d, J = 9.6 Hz, 1H), 3.22-3.11 (br m, 1H), 2.49-2.29 (br m, 2H), 1.86-1.76 (br s, 1H), 1.40 (s, 9H), 1.32-1.20 (m, 3H), 1.27 (dd, J = 7.2, 7.2 Hz, 3H), 1.15 (br d, J = 5.4 Hz, 3H), 1.04-0.96 (br m, 3H), 0.79 (br d, J = 4.9 Hz, 3H); ^{13}C NMR (100 MHz, $CDCl_3$) ppm 164.5, 154.5, 144.6, 140.8, 139.7, 139.0, 131.0 ($^2J_{CF}$ = 32 Hz), 129.3, 127.8, 126.5 ($^1J_{CF}$ = 271 Hz), 125.5 ($^3J_{CF}$ = 3.6 Hz), 98.8, 80.7, 59.5, 29.7, 28.2, 27.3, 26.6, 24.1, 22.7, 21.4, 9.33; HRMS (ESI): Exact mass calcd for $C_{29}H_{37}F_3NaN_2O_6$ [M+Na]$^+$ 589.2501, found 589.2510.

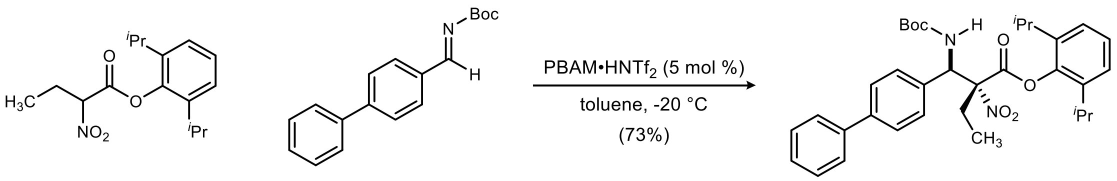

2,6-Diisopropylphenyl (*R*)-2-((*R*)-[1, 1'-biphenyl]-4-yl((*tert*-butoxycarbonyl)amino)methyl)-2-nitrobutanoate (13u). Following the general procedure, the imine (56.3 mg, 200 µmol), ester (64.5 mg, 220 µmol), and catalyst (7.9 mg, 10 µmol) in toluene (285 µmol) provided, after flash column chromatography (2-10% ethyl acetate in hexanes), the product as a colorless solid (84 mg, 73% yield[14]) in >20:1 dr and 99% ee; (Chiralcel AD-H, 5% ^{i}PrOH/hexanes, 1 mL/min, t_r(*anti*, minor) = 6.7 min, t_r(*syn*, major) = 8.4 min, t_r(*syn*, minor) = 8.9 min, t_r(*anti*, major) = 12.9 min); mp = 59-63 °C; R_f = 0.33 (10% EtOAc/hexanes); $[\alpha]_D^{25}$ -18.3 (c 1.0, $CHCl_3$); IR (film) 3441, 2967, 1747, 1720, 1558, 1488, 1366, 1225, 1159 cm^{-1}; ^{1}H NMR (400 MHz, $CDCl_3$) δ 7.61 (d, J = 8.0 Hz, 4H), 7.53-7.45 (br m, 4H), 7.40 (br dd, J = 7.4, 7.4 Hz, 1H), 7.30-7.20 (br m, 2H), 7.09 (br d, J = 4.2

---

[13] The *syn* diastereomer was inseparable with this assay.
[14] Contaminated with the parent aldehyde.

Hz, 1H), 6.73 (br d, *J* = 9.6 Hz, 1H), 5.79 (br d, *J* = 9.7 Hz, 1H), 3.35-3.20 (br m, 1H), 2.60-2.35 (br m, 2H), 1.97-1.82 (br m, 1H), 1.42 (s, 9H), 1.30 (br dd, *J* = 7.1, 7.1 Hz, 3H), 1.31-1.25 (br m, 3H), 1.18 (br d, *J* = 7.1 Hz, 3H), 0.96 (br d, *J* = 5.8 Hz, 3H), 0.80 (br d, *J* = 5.7 Hz, 3H); ^{13}C NMR (100 MHz, $CDCl_3$) ppm 165.6, 154.6, 144.7, 141.5, 140.1, 134.3, 130.2, 129.2, 128.9, 127.7, 127.4, 127.2, 127.0, 99.0, 80.3, 59.7, 29.9, 28.2, 27.2, 26.6, 24.2 (2C), 22.8, 21.6, 9.42; HRMS (ESI): Exact mass calcd for $C_{34}H_{42}N_2NaO_6$ $[M+Na]^+$ 597.2941, found 597.2944.

(br s, 2H), 5.99 (br d, *J* = 9.5 Hz, 1H), 5.18 (br d, *J* = 9.7 Hz, 1H), 3.19-2.77 (br m, 1H), 2.61 (br s, 1H), 1.66 (s, 3H), 1.44 (s, 9H), 1.21 (br s, 3H), 1.10 (br s, 3H), 0.98 (br s, 3H), 0.88 (br s, 3H) [N*H*$_2$ not observed]; ^{13}C NMR (100 MHz, $CDCl_3$) ppm 173.9, 155.5, 145.2, 140.0, 139.1, 128.5, 128.3, 128.1, 127.7, 126.6, 123.8, 79.4, 62.3, 59.1, 28.3, 28.0, 26.9, 25.6, 23.9, 22.5, 22.0; HRMS (ESI): Exact mass calcd for $C_{27}H_{39}N_2O_4$ $[M+H]^+$ 455.2910, found 455.2927.

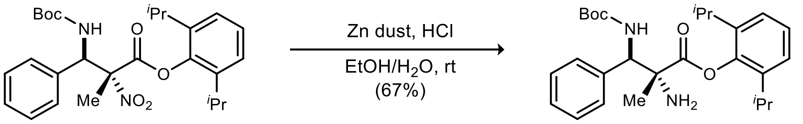

2,6-Diisopropylphenyl (2*R*,3*R*)-2-amino-3-((*tert*-butoxycarbonyl)amino)-2-methyl-3-phenylpropanoate (14). To a solution of nitroester **SI-1** (3.2 g, 6.60 mmol) in ethanol (66 mL) was added aq 2 M HCl (132 mL) followed by freshly purified zinc dust[15] (17.3 g, 264 mmol). The resulting suspension was stirred at room temperature for 6 h. Satd aq $NaHCO_3$ was added until the reaction mixture was basic, and the ethanol was removed *in vacuo*. The residue was redissolved in EtOAc, and washed with satd aq $NaHCO_3$, and then dried and concentrated. Flash column chromatography (SiO_2, 10%-20% ethyl acetate in hexanes) afforded the desired amine as a colorless solid (2.0 g, 67% yield). Mp = 129-131 °C; $[\alpha]_D^{20}$ -11 (*c* 0.80, $CHCl_3$); R_f = 0.10 (10% EtOAc/hexanes); IR (film) 3391, 1748, 1715, 1162 cm^{-1}; ^{1}H NMR[16] (400 MHz, $CDCl_3$) δ 7.50-7.41 (br m, 3H), 7.36-7.28 (series of br m, 3H), 7.09 (br s, 2H), 5.99 (br d, *J* = 9.5 Hz, 1H), 5.18 (br d, *J* = 9.7 Hz, 1H), 3.19-2.77 (br m, 1H), 2.61 (br s, 1H), 1.66 (s, 3H), 1.44 (s, 9H), 1.21 (br s, 3H), 1.10 (br s, 3H), 0.98 (br s, 3H), 0.88 (br s, 3H) [N*H*$_2$ not observed]; ^{13}C NMR (100 MHz, $CDCl_3$) ppm 173.9, 155.5, 145.2, 140.0, 139.1, 128.5, 128.3, 128.1, 127.7, 126.6, 123.8, 79.4, 62.3, 59.1, 28.3, 28.0, 26.9, 25.6, 23.9, 22.5, 22.0; HRMS (ESI): Exact mass calcd for $C_{27}H_{39}N_2O_4$ $[M+H]^+$ 455.2910, found 455.2927.

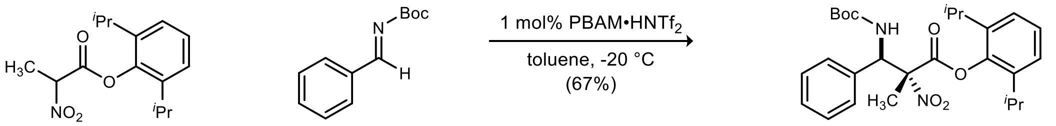

2,6-Diisopropylphenyl (2*R*,3*R*)-3-((*tert*-butoxycarbonyl)amino)-2-methyl-2-nitro-3-phenylpropanoate (SI-1). In a flame-dried, round bottom flask the imine (3.00 g, 14.6 mmol) and catalyst (115 mg, 146 µmol), were dissolved in toluene and chilled to -20 °C. The nitroester (5.00 g, 17.5 mmol) was added and the resulting mixture was allowed to stir at -20 °C for 3 days. To remove the catalyst, the solution was filtered cold through a small pad of silica using ethyl acetate and concentrated to a brown oil. Flash column chromatography (SiO_2, 1.5%-5% ethyl

[15] Purified according to: Armarego, W.L.F, and Perrin, D.D., *Purification of Laboratory Chemicals*, 4th ed.; Butterworth-Heinemann: Boston, 1998.
[16] Hindered rotation is confirmed by variable temperature ^{1}H NMR. Data is reported for major rotamer.

acetate in hexanes) afforded the product as a white foam (4.72 g, 67% yield) in >20:1 dr and 99% ee (Chiralcel IA, 3% ^{i}PrOH/hexanes, 1 mL/min) t_r(*anti*, minor) = 7.8 min, t_r(*syn*, major) = 10.0 min t_r(*anti*, major) = 12. 7 min, t_r(*syn*, minor) = 17.5 min; mp = 48.0-50.0 °C; $[\alpha]_D^{25}$ +3.1 (*c* 0.55, $CHCl_3$); R_f = 0.15 (3% EtOAc/hexanes); IR (film) 3444, 1753, 1723, 1560, 1163 cm^{-1}; ^{1}H NMR (500 MHz, DMSO-d_6) δ 8.01 (br d, *J* = 10.5 Hz, 1H), 7.44-7.33 (series of br m, 5H), 7.31-7.20 (series of m, 3H), 5.93 (br d, *J* = 10.0 Hz, 1H), 2.85-2.68 (br m, 2H), 1.97 (s, 3H), 1.34 (s, 9H), 1.12 (br d, *J* = 7.0 Hz, 6H), 1.10 (br d, *J* = 7.5 Hz, 6H); ^{13}C NMR (125 MHz, DMSO-d_6) ppm 164.2, 154.8, 144.5, 139.8, 135.8, 128.6, 128.5, 128.4, 127.4, 124.2, 95.7, 79.0, 57.8, 28.1, 26.4, 23.5, 22.5, 17.3; HRMS (ESI): Exact mass calcd for $C_{27}H_{36}N_2NaO_6$ $[M+Na]^+$ 507.2471, found 507.2464.
